# Assessing cross-national inequalities and predictive trends in gout burden: a global perspective (1990–2021)

**DOI:** 10.3389/fmed.2025.1527716

**Published:** 2025-03-27

**Authors:** Mingyang Li, Qilong Nie, Qilin Xia, Zeping Jiang

**Affiliations:** ^1^The Eighth Clinical Medical College, Guangzhou University of Chinese Medicine, Foshan, Guangdong, China; ^2^Foshan Hospital of Traditional Chinese Medicine, Guangzhou University of Chinese Medicine, Foshan, Guangdong, China

**Keywords:** gout, Global Burden of Disease, decomposition analysis, advanced analysis, prediction analysis, cross-national inequalities

## Abstract

**Background:**

Gout, caused by hyperuricemia and the deposition of monosodium urate crystals in joints, remains a major global health issue. Despite progress in treatment, its prevalence continues to rise, contributing to comorbidities like cardiovascular and chronic kidney diseases. Understanding global trends and sociodemographic disparities is crucial for developing targeted interventions.

**Methods:**

We analyzed gout prevalence, incidence, and disability-adjusted life years (DALYs) from 1990 to 2021, stratified by age, sex, and economic development. Decomposition analysis quantified the impact of demographic factors, while advanced analysis assessed the relationship between gout burden and socioeconomic development. Prediction models forecasted future trends, and cross-national inequalities were evaluated to highlight disparities across regions with different development levels.

**Results:**

Between 1990 and 2021, the global prevalence of gout increased from 22,264,515 (95% UI: 17,793,190–27,965,605) to 56,474,572 (95% UI: 45,161,987–70,288,316), with the age-standardized prevalence rate (ASPR) rising from 536.54 to 653.82 per 100,000 population [(Estimated annual percentage changes) EAPC: 0.87%, 95% CI: 0.80–0.95]. The incidence of gout cases increased by 136.1%, with the age-standardized incidence rate (ASIR) rising by 17.12% over this period. Similarly, the age-standardized death rate (ASDR) grew by 21.30%, accompanied by a substantial increase in DALYs. Decomposition analysis revealed that aging significantly contributed to increased gout prevalence in Middle SDI regions (36.79%), while population growth was the dominant factor in Low SDI regions (98.58%). Advanced analysis indicated substantial gaps between observed gout burden and optimal levels in high-SDI countries, such as the United States and Australia, highlighting unrealized opportunities for improving outcomes. Prediction analysis projected a stable global burden of gout from 2021 to 2045, with notable gender-specific and age-specific trends. Cross-national inequality analysis showed worsening disparities in gout prevalence, incidence, and DALYs between high- and low-SDI regions, reflected in increasing Slope Index of Inequality and Health Concentration Index values from 1990 to 2021.

**Conclusion:**

The global burden of gout has risen, with high-SDI regions facing risks from lifestyle changes and obesity, while low-SDI regions struggle with healthcare access. Public health strategies should focus on modifiable risk factors, healthcare infrastructure, and gender- and age-specific trends to address disparities.

## 1 Introduction

Gout is a treatable disease resulting from monosodium urate crystal deposition in joints and tissues, primarily driven by elevated serum urate levels (hyperuricaemia). When urate exceeds the saturation threshold, crystals form and deposit in tissues, triggering the innate immune system. This activation, particularly of the NOD-like receptor family pyrin domain-containing 3 (NLRP3) inflammasome, leads to Interleukin-1β (IL-1β) release, causing acute, painful inflammatory arthritis characteristic of gout flares (1, 2).

Recent reports indicate that the prevalence of gout varies widely depending on the population studied and the methods used, ranging from less than 1% to 6.8%, with an incidence rate between 0.58 and 2.89 per 1,000 person-years (3). Studies conducted across Asia, Europe, and North America have reported gout incidence rates ranging from 0.6 to 2.9 per 1,000 person-years, with prevalence rates between 0.68% and 3.90% in the adult population. Throughout the 20th century, the prevalence of gout showed a consistent upward trend (4–9).

As the global prevalence of gout continues to rise, it is essential to examine the key factors driving this trend, particularly the increasing rates of obesity, shifting dietary patterns, and the growing aging population. First, obesity stands out as one of the primary risk factors for gout. Obesity significantly contributes to hyperuricemia (high levels of uric acid in the blood), a key precursor to gout. Excessive adipose tissue not only increases the production of uric acid but also impairs its renal excretion (10, 11). Specifically, abdominal obesity is closely linked to elevated uric acid levels, as visceral fat cells release hormones that interfere with the kidneys’ ability to filter and excrete uric acid effectively (12, 13). Furthermore, obesity is associated with insulin resistance and low-grade chronic inflammation, both of which exacerbate uric acid production (14). As obesity rates rise globally, this increasingly prevalent condition has become a central driver of gout’s increasing incidence (15).

Second, the shift in dietary habits has played a significant role in the rise of gout. Modern dietary patterns, particularly those influenced by Western diets, often include high-purine foods, such as red meat, shellfish, and alcohol, which are major contributors to uric acid buildup. Purines, found abundantly in animal products, break down into uric acid during metabolism. Overconsumption of purine-rich foods leads to a higher likelihood of hyperuricemia, thereby increasing the risk of gout flare-ups (16, 17). Alcohol consumption, particularly beer and spirits, is another critical factor. Not only does alcohol elevate uric acid production, but it also impairs kidney function, reducing uric acid clearance and exacerbating hyperuricemia. Additionally, the increased consumption of sugar-sweetened beverages, particularly those high in fructose, has been linked to elevated uric acid levels. Fructose metabolism increases the synthesis of uric acid, further contributing to gout (18, 19). These dietary shifts, characterized by the overconsumption of high-purine foods and alcohol, are thus pivotal drivers of gout’s growing prevalence.

Finally, the aging population is another significant factor in the rising rates of gout. As life expectancy increases globally, gout is becoming more common among older adults. Aging leads to a gradual decline in renal function, including a reduced ability to excrete uric acid, which can cause its accumulation in the body. This is particularly evident in individuals over the age of 60, where the incidence of gout rises markedly (20). In women, the risk of gout also increases post-menopause due to a decline in estrogen, which plays a role in uric acid excretion. In addition to age-related changes in metabolism, older adults often have multiple chronic conditions, such as hypertension, diabetes, and obesity, which further increase the risk of hyperuricemia and gout (21–24). Furthermore, medications commonly prescribed to older individuals, such as diuretics, may exacerbate gout by elevating uric acid levels (25, 26). As such, the aging population represents both a direct and indirect driver of gout’s increasing prevalence, as age-related physiological changes and comorbid conditions contribute to higher rates of the disease.

Given the rising global burden of gout, a thorough analysis of its epidemiological trends is critical. For this study, we utilized data from the Global Burden of Disease (GBD) 2021 database, which provides comprehensive data on gout incidence, prevalence, and disability-adjusted life years (DALYs) across 204 countries and territories from 1990 to 2021. This allowed us to examine global and regional patterns of gout burden in detail.

We applied several advanced statistical methods. Decomposition analysis quantified the contributions of population aging, growth, and epidemiological changes to gout trends, while advanced analysis assessed the relationship between gout burden and sociodemographic development, identifying areas for potential improvement. Using the Nordpred model, we conducted prediction analysis to forecast future trends in gout burden from 2021 to 2045, providing valuable insights for public health strategies. Finally, cross-national inequalities analysis revealed significant disparities in gout outcomes between high and low SDI regions, highlighting global inequalities.

## 2 Materials and methods

### 2.1 Data source

The GBD project remains one of the largest and most systematic epidemiological research initiatives globally. The GBD 2021 study, released in 2024, provides a comprehensive analysis of 371 diseases and injuries, along with 88 risk factors worldwide. For our analysis, we extracted global data on prevalence, incidence, and DALYs, as well as population estimates for gout from the GBD 2021 database. These data were further stratified by age group, sex, year, country, and region. The GBD 2021 data can be accessed via web-based tools.^1^ Additionally, the study employed the Sociodemographic Index (SDI), which assesses a region’s development level by incorporating factors such as income, education, and fertility rates. The gout cases in our study were defined based on ICD-10/ICD-9 classification criteria in Supplementary Table 1.

### 2.2 Statistical analysis

The terms and definitions related to the GBD utilized in this study are provided in Supplementary Table 1. Crude prevalence, incidence, and DALY rates are fundamental measures for assessing the epidemiological trends of disease. However, differences in population age structure can lead to variations in the burden of gout. To ensure comparability of these indicators across populations, we adjusted the crude rates by applying weights based on age distribution, resulting in age-standardized rates (ASR). The detailed methodology for calculating ASR is outlined in the Supplementary Methods. Consequently, age-standardized prevalence rates (ASPR), age-standardized incidence rates (ASIR), and age-standardized death rates (ASDR) were used to assess the burden of gout in this study.

### 2.3 Decomposition analysis

We applied the decomposition analysis method originally proposed by Das Gupta (27), in combination with the enhanced approach developed by Cheng et al. (28) in 2020, to disaggregate changes in gout prevalence, incidence, and DALYs. The core strength of decomposition analysis lies in its ability to break down overall variations in an outcome into the contributions of distinct factors. In this study, we decomposed the observed changes in gout prevalence, incidence, and DALYs into three primary group-level determinants: population aging, population growth, and epidemiological change. This approach enabled us to quantify the specific contribution of each factor to the total change, offering a more nuanced understanding of how demographic and epidemiological dynamics influence mortality trends. By employing this method, we were able to evaluate both the magnitude and relative importance of each determinant in shaping the overall trend. Further details on the decomposition analysis methodology can be found in the Supplementary Methods.

### 2.4 Advanced analysis

To assess the relationship between the burden of gout and sociodemographic development, we utilized advanced analysis as a quantitative method. This approach allowed us to identify the lowest potentially achievable ASPR, ASIR, and ASDR based on a country’s SDI. The SDI is a composite measure of development, encompassing indicators such as income per capita, educational attainment, and fertility rates. A detailed description of the advanced analysis methodology is provided in the Supplementary Methods.

### 2.5 Prediction analysis

To project the future burden of gout in terms of prevalence, incidence, DALYs, and ASPR, ASIR, ASDR from 2021 to 2045, we utilized the Nordpred model, which employs an age-period-cohort (APC) regression framework. This model applies a Poisson log-link function to estimate future mortality trends based on historical data, accounting for the effects of age, time period, and birth cohort (29, 30). The underlying assumption is that the number of deaths adheres to a Poisson distribution, with non-linear trends incorporated through the log-link function. Using country-specific historical data on gout prevalence, incidence, DALYs, and ASPR, ASIR, and ASDR, we forecasted the future values of these metrics from 2021 to 2045. The model adjusts for variations in population size, age structure, and other demographic factors over time, thereby providing robust projections of the disease burden. Additionally, we calculated percentage changes in predicted deaths and ASDR to evaluate future trends, which will inform public health planning initiatives (29, 30).

### 2.6 Cross-national inequalities analysis

To quantify the sociodemographic inequality associated with gout prevalence, incidence, and DALYs, we employed the slope index of inequality (SII) and the concentration index, standard measures for absolute and relative inequality, respectively (31). The SII was calculated by regressing country-specific gout prevalence and incidence rates, as well as DALYs, against a relative positional scale based on the SDI, with relative position determined by the midpoint of each population’s cumulative range ranked by SDI. This method captures the absolute gradient of inequality by adjusting for population size and utilizing weighted regression models to address heteroscedasticity. The concentration index quantifies relative inequality by fitting a Lorenz concentration curve to the cumulative distribution of gout-related prevalence, incidence, and DALYs compared against the population ranked by SDI. The area under the Lorenz curve was numerically integrated to derive the concentration index, with higher values indicating greater inequality (32, 33). A concentration index value between 0.2 and 0.3 suggests a substantial degree of relative inequality, underscoring the uneven distribution of gout burden across various sociodemographic contexts. This analysis reveals disparities in gout-related health outcomes among countries with differing levels of sociodemographic development (34).

## 3 Results

### 3.1 Prevalence, incidence, DALYs of the global gout burden from 1990 to 2021

Globally, the prevalence of gout increased significantly from 22,264,515 (95% [Uncertainty Interval] UI: 17,793,190–27,965,605) in 1990 to 56,474,572 (95% UI: 45,161,987–70,288,316) in 2021. The ASPR rose from 536.54 per 100,000 (95% UI: 430.28–665.72) in 1990 to 653.82 per 100,000 (95% UI: 526.13–810.46) in 2021. The (Estimated annual percentage changes) EAPC in ASPR was 0.87% (95% [Confidence Interval] CI: 0.80–0.95) from 1990 to 2021 (Table 1).

**TABLE 1:** Prevalence and ASPR of gout in 1990 and 2021and the EAPC in ASPR from 1990 to 2021.

Prevalence	1990		2021		1990–2021
**Location**	**Prevalence case no. (95% UI)**	**ASPR per 100,000 no. (95% UI)**	**Prevalence case no. (95% UI)**	**ASPR per 100,000 no. (95% UI)**	**EAPC in ASPR no. (95% CI)**
Global	22,264,515 (17,793,190–27,965,605)	536.54 (430.28–665.72)	56,474,572 (45,161,987–70,288,316)	653.82 (526.13–810.46)	0.87 (0.80–0.95)
Andean Latin America	51,605 (41,516–64,630)	220.94 (177.02–278.8)	180,586 (144,673–224,051)	290.08 (233.67–362.3)	1.00 (0.96–1.04)
Australasia	248,608 (197,219–310,655)	1,073.11 (855.95–1,338.49)	698,923 (542,306–895,103)	1,423.86 (1,129.55–1,802.46)	0.97 (0.92–1.03)
Caribbean	53,978 (43,346–66,977)	196.94 (159.04–245.25)	131,010 (105,299–162,972)	247.08 (198.52–305.92)	0.77 (0.75–0.79)
Central Asia	184,788 (147,118–233,132)	377.31 (301.58–469.02)	389,491 (305,426–494,227)	444.78 (356.14–553.83)	0.57 (0.54–0.61)
Central Europe	464,502 (366,994–587,612)	317.16 (254.67–395.9)	724,981 (570,686–908,777)	366.08 (293.24–457.99)	0.47 (0.46–0.49)
Central Latin America	155,990 (123,740–194,321)	154.88 (123.38–192.98)	493,737 (392,477–621,425)	189.7 (151.62–237.45)	0.77 (0.72–0.82)
Central Sub-Saharan Africa	103,398 (80,988–132,364)	420.87 (335.74–536)	278,007 (218,338–353,254)	429.01 (342.16–536.92)	0.06 (−0.00–0.12)
East Asia	6,241,229 (4,957,874–7,851,316)	645.4 (515.92–802.01)	17,461,754 (13,696,363–22,109,338)	814.66 (649.39–1,014.04)	1.04 (0.94–1.15)
Eastern Europe	1,021,278 (809,637–1,291,814)	373.93 (300.05–468.59)	1,416,473 (1,116,639–1,778,197)	431.6 (343.52–537.5)	0.48 (0.47–0.49)
Eastern Sub-Saharan Africa	347,820 (275,229–439,220)	425.06 (340.06–528.4)	877,459 (699,294–1,103,726)	448.47 (357.77–560.37)	0.20 (0.17–0.22)
High-income Asia Pacific	1,311,301 (1,031,284–1,659,381)	642.14 (508.11–808.56)	2,705,291 (2,133,093–3,444,910)	726.97 (570.95–918.04)	0.38 (0.37–0.40)
High-income North America	3,230,237 (2,600,262–4,050,064)	971.4 (771.5–1216.38)	9,499,879 (7,724,481–11,694,564)	1,658.09 (1,349.93–2,024.88)	2.55 (2.27–2.83)
North Africa and Middle East	808,017 (639,611–1,022,399)	437.11 (349.69–545.19)	2,668,764 (2,108,892–3,388,607)	527.38 (422.94–658.59)	0.61 (0.59–0.63)
Oceania	22,345 (17,518–28,732)	650.4 (516.39–811.79)	61,700 (48,535–78,555)	703.37 (563.26–881.4)	0.22 (0.21–0.23)
South Asia	2,464,630 (1,959,464–3,114,029)	389.91 (313.86–486.66)	6,575,742 (5,226,387–8,293,296)	421.37 (338.49–527.44)	0.25 (0.24–0.26)
Southeast Asia	1,526,964 (1,215,090–1,919,977)	527.97 (423.55–654.59)	4,548,364 (3,581,211–5,773,700)	645.06 (513.55–802.49)	0.70 (0.68–0.73)
Southern Latin America	347,889 (275,337–435,397)	748.19 (591.85–936.53)	777,982 (617,449–981,209)	929.17 (733.86–1,167.05)	0.67 (0.62–0.71)
Southern Sub-Saharan Africa	146,696 (116,218–184,591)	501.55 (401.9–623.84)	344,340 (273,347–434,373)	548.26 (438.99–682.94)	0.33 (0.30–0.37)
Tropical Latin America	208,286 (165,324–259,759)	200.83 (161.22–250.71)	663,470 (531,870–833,973)	255.12 (205.52–317.91)	0.81 (0.78–0.83)
Western Europe	2,915,727 (2,307,820–3,692,387)	545.63 (432.06–685.98)	4,974,989 (3,928,418–6,293,713)	627.32 (497.13–792.56)	0.51 (0.46–0.56)
Western Sub-Saharan Africa	409,228 (326,319–516,117)	425.64 (340.39–532.69)	1,001,631 (794,864–1,259,804)	435.8 (349.28–543.4)	0.05 (0.01–0.09)
High SDI	7,480,327 (9,386,660–5,992,568)	703.84(874.69–562.31)	18,244,623 (22,825,205–14,737,706)	1,007.00(1,246.1–811.48)	1.55 (1.42, 1.67)
Low SDI	986,741 (1,253,516–783,116)	400.65 (500.30–321.41)	2,409,623 (3,037,971–1,919,554)	420.54 (525.41–338.25)	0.17 (0.15, 0.18)
Middle SDI	5,789,274 (7,320,043–4,591,291)	497.52 (620.21–399.36)	16,289,579 (20,576,152–12,895,686)	587.79 (729.98–471.04)	0.73 (0.65, 0.81)
High-middle SDI	5,352,002 (6,730,522–4,252,401)	525.25 (654.24–419.75)	12,827,157 (16,197,588–10,134,430)	674.92 (837.46–538.98)	0.97 (0.91, 1.03)
Low-middle SDI	2,640,087 (3,323,801–2,107,993)	394.71 (492.02–317.24)	6,671,652 (8,446,242–5,304,075)	433.57 (543.17–346.94)	0.30 (0.29, 0.32)

In 1990, the total incidence of gout cases was 3,983,109 (95% UI: 3,178,834–4,911,691), rising to 9,401,585 (95% UI: 7,438,817–11,731,815) by 2021, marking a 136.1% increase over the 31-year period. Additionally, the ASIR increased from 93.1 per 100,000 (95% UI: 74.4–115.48) in 1990 to 109.07 per 100,000 (95% UI: 86.38–135.76) in 2021. The EAPC in ASIR was 0.67% (95% CI: 0.61–0.73) over the same period (Table 2).

**TABLE 2:** Incidence and ASIR of gout in 1990 and 2021 and the EAPC in ASIR from 1990 to 2021.

Incidence	1990		2021		1990–2021
**Location**	**Incidence case no. (95% UI)**	**ASIR per 100,000 no. (95% UI)**	**Incidence case no. (95% UI)**	**ASIR per 100,000 no. (95% UI)**	**EAPC in ASIR no. (95% CI)**
Global	3,983,109 (3,178,834,4,911,691)	93.1 (74.4, 115.48)	9,401,585 (7,438,817,11,731,815)	109.07 (86.38,135.76)	0.67 (0.61–0.73)
Andean Latin America	10,436 (8,514,12,949)	42.99 (35.06, 53.68)	35,110 (28,111,43,518)	55.78 (44.83, 69.28)	0.93 (0.90–0.97)
Australasia	33,295 (26,658,41,208)	146.02 (116.6, 180.63)	81,519 (63,532,102,287)	179.39 (140.41, 221.92)	0.66 (0.61–0.70)
Caribbean	10,846 (8,865,13,407)	38.7 (31.42, 48.24)	25,341 (20,460,31,558)	48.07 (38.99,59.77)	0.73 (0.71–0.75)
Central Asia	36,518 (29,006,45,336)	72.55 (57.75, 91.06)	75,179 (59,084,93,396)	83.85 (66.63, 104.81)	0.51 (0.48–0.54)
Central Europe	90,905 (71,676,114,450)	62.39 (49.66, 77.99)	136,145 (108,888,172,034)	71.26 (56.69, 88.9)	0.44 (0.42–0.46)
Central Latin America	31,517 (25,308,39,402)	29.85 (24.15, 37.14)	95,849 (77,252,119,428)	36.75 (29.76, 45.89)	0.78 (0.73–0.83)
Central Sub-Saharan Africa	21,178 (16,663,26,277)	80.74 (64.37, 100.81)	57,576 (45,451,71,273)	82.88 (65.63, 102.95)	0.09 (0.04–0.15)
East Asia	1,233,027 (979,951,1,521,565)	123.14 (98.59, 153.48)	3,193,828 (2,518,307,4,031,445)	151.95 (121.57,189.49)	0.95 (0.85–1.05)
Eastern Europe	201,072 (158,941,251,351)	74.08 (58.92, 92.81)	269,935 (213,098,342,015)	84.4 (67.08,105.65)	0.44 (0.43–0.45)
Eastern Sub-Saharan Africa	71,575 (56,656,88,741)	82.55 (65.53, 103.8)	181,819 (145,160,225,424)	86.92 (69.3,109.17)	0.19 (0.16–0.21)
High-income Asia Pacific	214,005 (168,690,270,048)	105.13 (82.86, 132.29)	393,651 (309,045,498,177)	114.31 (90.28, 143)	0.22 (0.19–0.25)
High-income North America	439,972 (347,527,544,700)	134.06 (106.12,164.61)	1,011,555 (808,282,1,237,427)	190.54 (154.07, 230.1)	1.73 (1.51–1.96)
North Africa and Middle East	162,366 (127,796,201,160)	83.73 (66.18, 104.32)	521,581 (412,775,651,411)	99.48 (79.18, 124.59)	0.56 (0.54–0.58)
Oceania	4,488 (3,513,5,603)	122.45 (97.79,151.64)	12,133 (9,594,15,044)	130.85 (104.86, 163.73)	0.19 (0.18–0.20)
South Asia	506,795 (401,712,626,444)	76.61 (60.88,95.91)	1,319,686 (1,043,791,1,649,127)	82.57 (65.46, 103.6)	0.24 (0.23–0.25)
Southeast Asia	307,926 (245,312,377,305)	101.57 (81.12, 126.54)	871,578 (687,330,1,087,857)	121.8 (96.87,152.04)	0.64 (0.62–0.65)
Southern Latin America	50,928 (39,827,63,533)	108.93 (85.25, 135.55)	104,536 (81,637,129,948)	127.91 (99.76,158.83)	0.45 (0.41–0.49)
Southern Sub-Saharan Africa	29,555 (23,358,36,748)	96.69 (76.28, 120.99)	68,423 (53,678,85,256)	105.25 (83.93, 131.78)	0.32 (0.28–0.35)
Tropical Latin America	42,272 (33,979,52,496)	39.47 (31.92, 49.54)	129,210 (104,133,161,462)	49.9 (40.37, 61.95)	0.79 (0.76–0.82)
Western Europe	401,271 (317,056,501,412)	78.57 (61.84, 97.79)	611,641 (481,843,765,244)	84.9 (67.39, 105.09)	0.27 (0.23–0.31)
Western Sub-Saharan Africa	83,162 (65,606,102,943)	82.74 (65.58, 103.64)	205,291 (163,126,255,112)	83.97 (66.3, 105.34)	0.02 (−0.02 to 0.05)
High SDI	1,093,512 (1,357,559,866,455)	104.60 (129.75, 82.61)	2,254,681 (2,791,942,1,794,295)	133.71 (163.88, 106.70)	1.04 (0.95, 1.13)
Low SDI	202,198 (250,554,159,911)	78.17 (97.75, 62.01)	496,765 (616,494,397,663)	82.13 (102.96,65.27)	0.17 (0.16, 0.18)
Middle SDI	1,159,451 (1,430,441,923,706)	95.73 (119.52, 76.51)	3,072,463 (3,860,429,2,428,707)	110.76 (138.92, 88.00)	0.64 (0.57, 0.71)
Low-middle SDI	537,185 (663,785,425,089)	77.00 (96.43, 61.07)	2,244,858 (2,821,821,1,774,628)	84.05 (105.28, 66.80)	0.28 (0.27, 0.29)
High-middle SDI	987,724 (1,220,268,787,171)	95.46 (118.65, 76.38)	1,327,009 (1,652,520,1,047,210)	120.82 (149.90, 95.77)	0.91 (0.85, 0.97)

The global burden of gout, as measured by DALYs, increased significantly. In 1990, gout was responsible for 697,541 (95% UI: 470,078–1,000,259), which rose to 1,747,546 (95% UI: 1,186,175–2,484,547) by 2021, representing a substantial increase. The ASDR also grew from 16.67 per 100,000 (95% UI: 11.25–23.95) in 1990 to 20.22 per 100,000 (95% UI: 13.77–28.77) in 2021. Over this period, the EAPC in ASDR was 0.86% (95% CI: 0.78–0.93) (Table 3).

**TABLE 3:** DALYs and ASDR of gout in 1990 and 2021 and the EAPC in ASDR from 1990 to 2021.

DALYs	1990		2021		1990–2021
**Location**	**DALYs case no. (95% UI)**	**ASDR per 100,000 no. (95% UI)**	**DALYs case no. (95% UI)**	**ASDR per 100,000 no. (95% UI)**	**EAPC in ASDR no. (95% CI)**
Global	697,541 (470,078,1,000,259)	16.67 (11.25,23.95)	1,747,546 (1,186,175,2,484,547)	20.22 (13.77,28.77)	0.86 (0.78–0.93)
Andean Latin America	1,658 (1,088,2,418)	7 (4.67,10.2)	5,737 (3,846,8,305)	9.17 (6.18,13.18)	0.98 (0.94–1.02)
Australasia	7,612 (5,136,10,809)	32.93 (22.21, 47.34)	21,231 (14,434,31,005)	43.72 (29.63, 63.87)	0.97 (0.91–1.02)
Caribbean	1,731 (1,148,2,498)	6.27 (4.19, 9.05)	4,121 (2,754,5,915)	7.79 (5.21, 11.2)	0.74 (0.72–0.76)
Central Asia	5,820 (3,882,8,449)	11.79 (7.84, 17.07)	12,279 (8,145,17,854)	13.88 (9.28, 20)	0.58 (0.54–0.61)
Central Europe	14,330 (9,616,20,491)	9.78 (6.6, 14)	22,120 (14,758,32,062)	11.33 (7.53, 16.45)	0.49 (0.47–0.51)
Central Latin America	5,051 (3,303,7,377)	4.94 (3.28,7.19)	15,761 (10,221,22,879)	6.04 (3.93, 8.74)	0.75 (0.71–0.79)
Central Sub-Saharan Africa	3,218 (2,062,4,648)	12.78 (8.45, 18.09)	8,736 (5,737,12,762)	13.14 (8.81, 19.06)	0.11 (0.04–0.17)
East Asia	199,224 (131,831,288,423)	20.35 (13.57, 29.34)	547,132 (367,554,786,457)	25.57 (17.26, 36.41)	1.03 (0.93–1.13)
Eastern Europe	31,426 (20,873,44,441)	11.51 (7.68, 16.34)	43,189 (29,323,62,413)	13.27 (8.98, 19.09)	0.50 (0.48–0.52)
Eastern Sub-Saharan Africa	10,890 (7,194,15,685)	13.02 (8.66, 18.77)	27,668 (18,283,39,961)	13.81 (9.29, 19.7)	0.22 (0.20–0.25)
High-income Asia Pacific	41,109 (27,800,59,500)	20.09 (13.61, 28.83)	83,017 (56,168,118,872)	22.8 (15.38, 32.77)	0.39 (0.38–0.41)
High-income North America	99,215 (67,184,141,426)	29.99 (20.2, 42.53)	285,459 (200,620,398,091)	50.49 (35.15, 70.96)	2.48 (2.21–2.76)
North Africa and Middle East	25,468 (16,887,36,840)	13.54 (9.12, 19.63)	83,285 (55,201,120,966)	16.21 (10.86, 23.29)	0.59 (0.57–0.60)
Oceania	707 (469,1,021)	20.12 (13.47, 28.33)	1,946 (1,283,2,881)	21.71 (14.49, 31.61)	0.21 (0.20–0.23)
South Asia	76,704 (50,881,111,178)	11.87 (7.95, 17.06)	203,439 (135,171,289,270)	12.88 (8.61, 18.66)	0.27 (0.26–0.28)
Southeast Asia	48,495 (32,201,70,571)	16.49 (11.18, 23.88)	143,387 (94,672,205,551)	20.15 (13.43, 28.8)	0.72 (0.69–0.74)
Southern Latin America	10,850 (7,328,15,606)	23.3 (15.72, 33.42)	23,982 (16,370,34,700)	28.76 (19.58, 41.52)	0.66 (0.61–0.70)
Southern Sub-Saharan Africa	4,576 (3,097,6,617)	15.43 (10.45, 22.41)	10,592 (7,139,15,344)	16.65 (11.32, 24.15)	0.30 (0.26–0.34)
Tropical Latin America	6,630 (4,329,9,568)	6.29 (4.19, 9.09)	20,696 (13,755,29,598)	7.94 (5.28, 11.33)	0.80 (0.77–0.83)
Western Europe	90,003 (61,210,129,071)	16.98 (11.5, 24.38)	152,033 (102,794,218,570)	19.51 (13.28, 28.2)	0.51 (0.46–0.56)
Western Sub-Saharan Africa	12,824 (8,533,18,415)	13.12 (8.76, 18.76)	31,737 (20,999,46,005)	13.51 (9.1, 19.51)	0.08 (0.04–0.11)
Middle SDI	184,017 (266,841,122,631)	15.58 (22.49, 10.51)	207,973 (294,639,138,611)	13.36 (19.19, 8.95)	0.72 (0.64, 0.80)
Low-middle SDI	82,666 (119,512,54,625)	12.13 (17.52, 8.08)	399,193 (576,622,268,085)	21.09 (30.28, 14.16)	0.32 (0.30, 0.33)
High SDI	231,388 (329,564,158,322)	21.86 (31.16, 14.84)	509,745 (731,535,341,438)	18.32 (26.21, 12.36)	1.51 (1.39, 1.63)
High-middle SDI	168,200 (240,462,112,126)	16.42 (23.67, 11.01)	553,992 (780,528,383,750)	31.02 (43.85, 21.33)	0.97 (0.91, 1.03)
Low SDI	30,764 (44,178,20,447)	12.24 (17.41, 8.21)	75,650 (108,116,50,161)	12.93 (18.67, 8.63)	0.20 (0.18, 0.21)

### 3.2 Prevalence, incidence, DALYs of the gout burden in 21 GBD regions from 1990 to 2021

**FIGURE 1:**
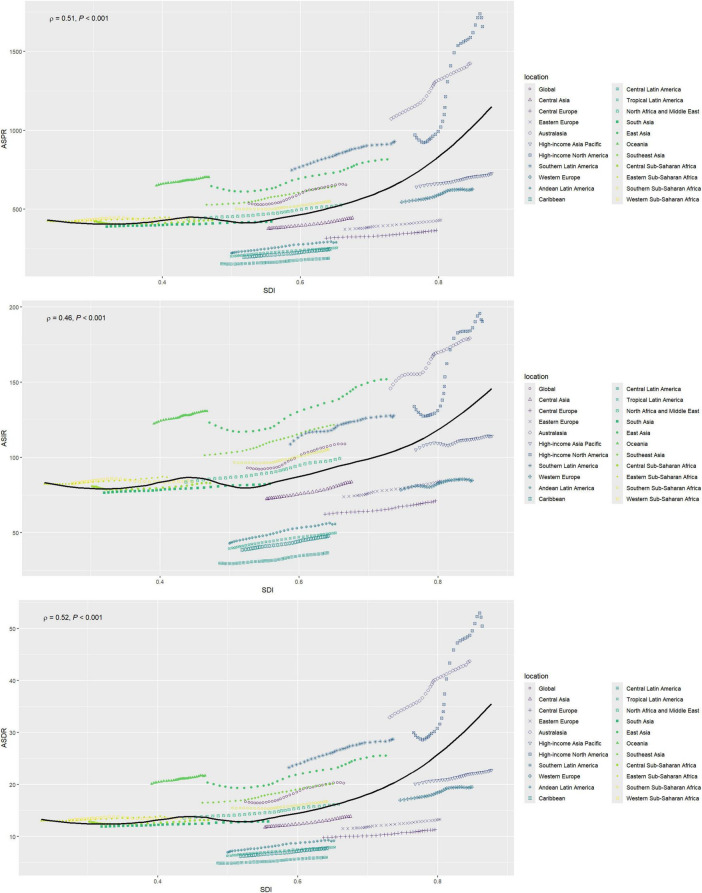
Changes in the ASPR, ASIR, and ASDR of gout globally and in different SDI regions. ASPR, age-standardized prevalence rate; ASIR, age-standardized incidence rate; ASDR, age-standardized DALY rate; SDI, sociodemographic index.

High-income North America consistently shows the highest rates across all metrics, with an ASPR of 1,658.09 per 100,000 (95% UI: 1,349.93–2,024.88), an ASIR of 190.54 per 100,000 (95% UI: 154.07–230.10), and an ASDR of 50.49 per 100,000 (95% UI: 35.15–70.96) (Figure 1 and Tables 1–3).

High-income North America consistently shows the highest rates of increase across all metrics, with an EAPC of 2.55 (95% CI: 2.27–2.83) for the ASPR, 1.73 (95% CI: 1.51–1.96) for the ASIR, and 2.48 (95% CI: 2.21–2.76) for the ASDR. In contrast, Western Sub-Saharan Africa consistently reports the lowest increases (Tables 1–3).

### 3.3 Prevalence, incidence, DALYs of the gout burden in 5 SDI regions from 1990 to 2021

In 2021, significant disparities in disease burden were observed across the five SDI regions, based on GBD data. The High SDI region recorded the highest ASPR at 1,007 per 100,000 (95% UI: 1,246.1–811.48) and the highest ASIR at 133.71 per 100,000 (95% UI: 106.70–163.88). In contrast, the Low SDI region had the lowest ASPR and ASIR, with an ASPR of 420.54 per 100,000 (95% UI: 525.41–338.25) and an ASIR of 82.13 per 100,000 (95% UI: 65.27–102.96).

Regarding ASDR, the High-middle SDI region reported the highest rate at 31.02 (95% UI: 21.33–43.85), while the Low SDI region had the lowest rate at 12.93 (95% UI: 8.63–18.67) (Tables 1–3).

From 1990 to 2021, the trends in EAPC for both ASPR, ASIRa nd ASDR also varied across SDI regions. The High SDI region showed the most pronounced increase in ASPR, with an EAPC of 1.55 (95% CI: 1.42–1.67), while the Low SDI region experienced the smallest rise at 0.17 (95% CI: 0.15–0.18). Similarly, the High SDI region recorded the largest increase in ASIR, with an EAPC of 1.04 (95% CI: 0.95–1.13), while the Low SDI region showed only a slight increase of 0.17 (95% CI: 0.16–0.18). In further analysis of ASDR trends, the High SDI region exhibited the highest EAPC at 1.51 (95% CI: 1.39–1.63), again surpassing all other regions, while the Low SDI region had the smallest increase at 0.20 (95% CI: 0.18–0.21) (Tables 1–3).

### 3.4 Prevalence, incidence, DALYs of the gout burden in 204 countries and territories from 1990 to 2021

**FIGURE 2:**
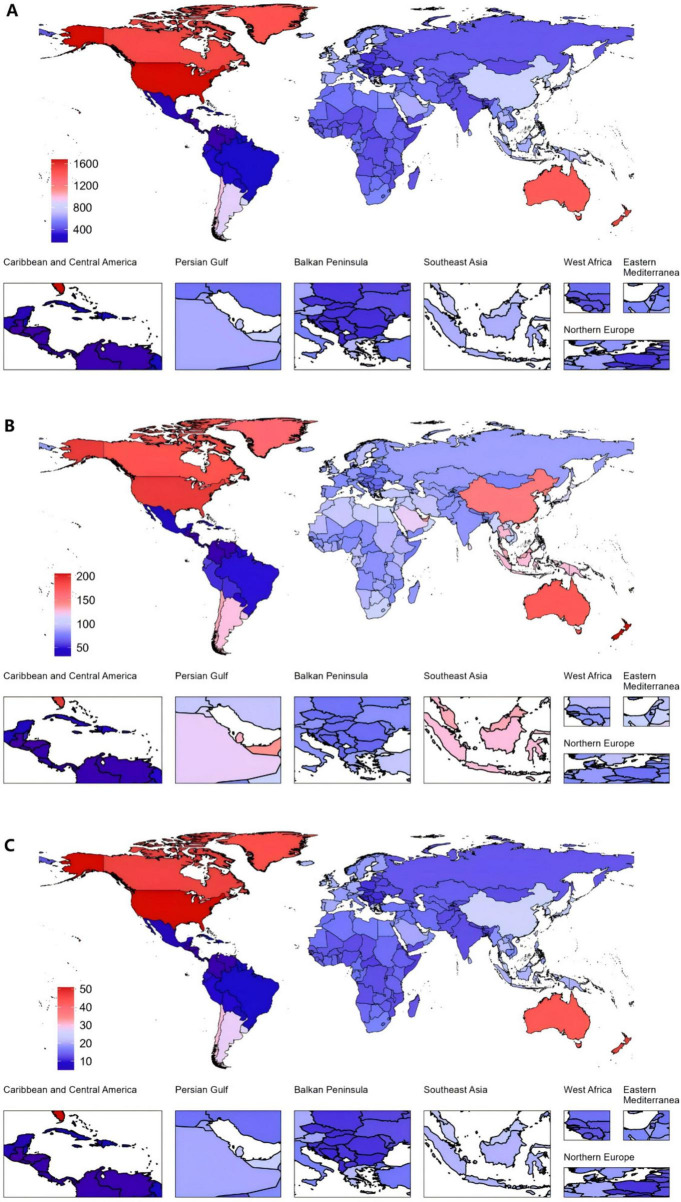
Global gout burden in 204 countries and territories. **(A)** ASPR, **(B)** ASIR, **(C)** ASDR in 2021. ASDR, age-standardized DALY rate; ASIR, age-standardized incidence rate; ASPR, age-standardized prevalence rate; DALYs, disability-adjusted life years.

In 2021, the United States of America had the highest ASPR for gout at 1677.1 per 100,000 (95% UI: 1369.5–2044.08), as well as the highest ASDR, with 50.95 per 100,000 people (95% UI: 35.36–71.75). New Zealand had the highest ASIR for gout, with 205.24 per 100,000 (95% UI: 161.12–256.24) (Figure 2 and Supplementary Tables 2–4).

**FIGURE 3:**
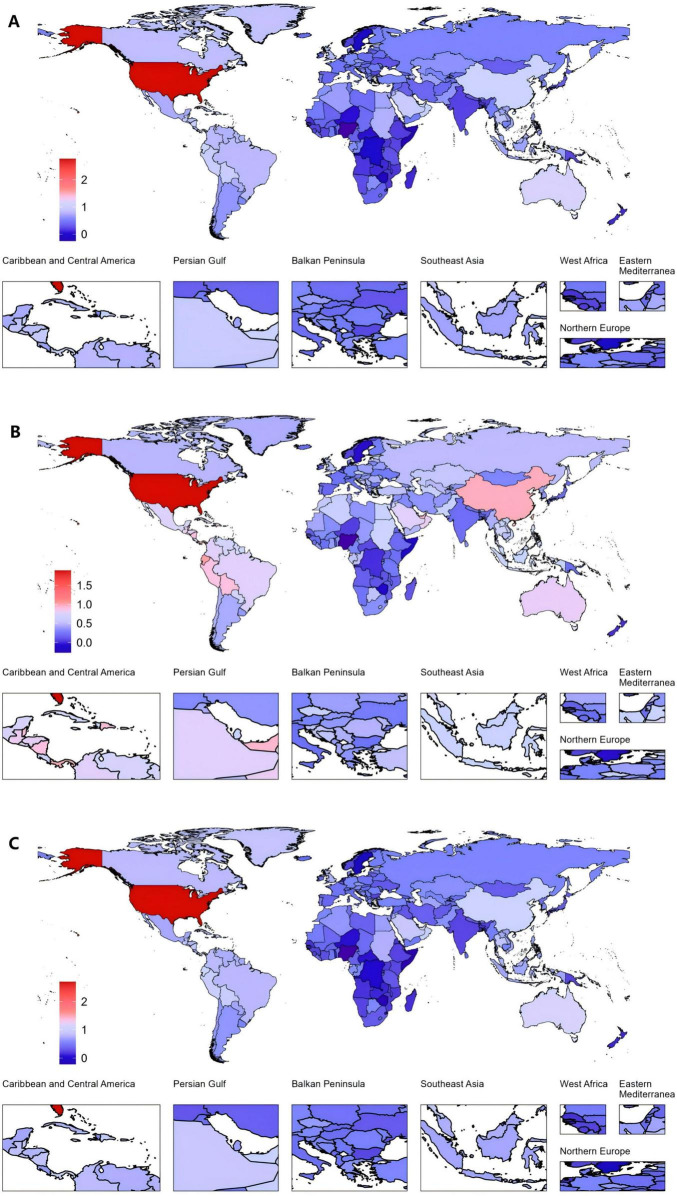
Global gout burden in 204 countries and territories. **(A)** EAPC in the ASPR from 1990 to 2021, **(B)** EAPC in the ASIR from 1990 to 2021, **(C)** EAPC in the ASDR from 1990 to 2021. ASDR, age-standardized DALY rate; ASIR, age-standardized incidence rate; ASPR, age-standardized prevalence rate; DALYs, disability-adjusted life years; EAPC, estimated annual percentage changes.

Among all countries, the United States of America exhibited the highest increase in EAPC from 1990 to 2021. The EAPC for the ASPR was 2.76 (95% CI: 2.44 to 3.09). The ASIR also increased markedly, with an EAPC of 1.89 (95% CI: 1.63 to 2.14). Furthermore, the ASDR saw a notable rise, with an EAPC of 2.70 (95% CI: 2.38 to 3.01). In contrast, Nigeria demonstrated the largest decreases across all three metrics (Figure 3 and Supplementary Tables 2–4).

### 3.5 Prevalence, incidence, DALYs of the global gout burden by age and gender from 1990 to 2021

**FIGURE 4:**
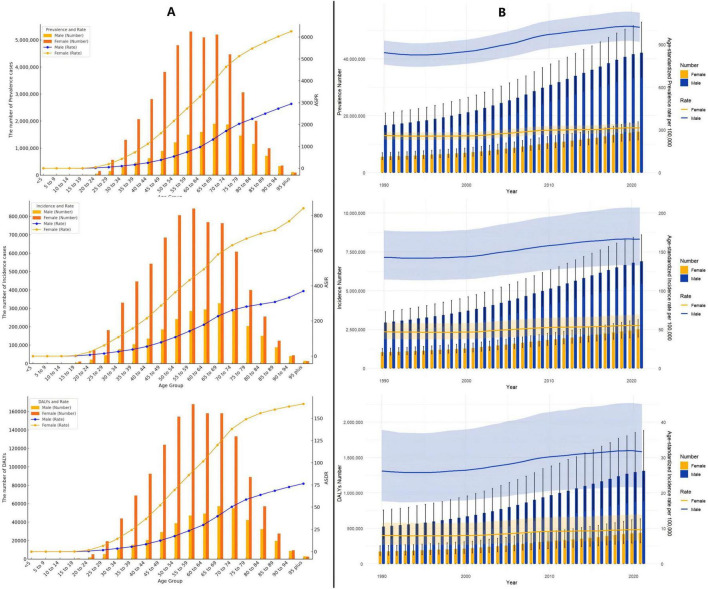
**(A)** Age-specific numbers and rates of prevalence, incidence, and DALYs of gout by age and gender in 2021; **(B)** age-specific numbers and rates of prevalence, incidence, and DALYs of gout by gender from 1990 to 2021.

In 2021, global data on gout revealed that males aged 55–59 exhibited the highest values across three key health indicators—DALYs, incidence, and prevalence—significantly exceeding those of females. However, while males consistently show higher values than females across most age groups, this trend reverses in the 95+ category, where females surpass males in all three indicators (Figure 4A and Supplementary Table 5).

From 1990 to 2021, both males and females exhibited a gradual increase in the ASPR, ASIR, and ASDR for gout, though the trends varied in magnitude and pace (Figure 4B and Supplementary Table 5).

### 3.6 Decomposition analysis of gout burden

**FIGURE 5:**
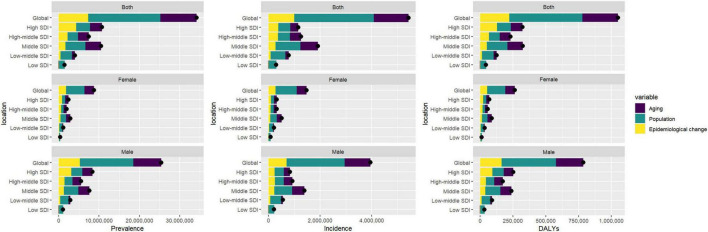
Gender-based decomposition analysis of gout indicators from 1990 to 2021. The black dot represents the overall change value of population growth, aging, and epidemiological change.

The global burden of gout is influenced by three major factors: population aging, population growth, and epidemiological change, with variations between men and women. For global prevalence, population aging contributes 26.39%, with 27.35% in females and 27.2% in males. Population growth has the most significant impact, contributing 52.3% globally, with 52.14% in females and 52.13% in males. Epidemiological change accounts for 21.32%, with 20.51% in females and 20.68% in males. Regarding global incidence, population aging contributes 24.76%, with 25.82% in females and 25.25% in males, while population growth again has the largest effect, contributing 56.71% globally, with 55.29% in females and 57.00% in males. Epidemiological change accounts for 18.53%, with 18.89% in females and 17.75% in males. Finally, for global DALYs, population aging contributes 25.90%, with 26.95% in females and 26.6% in males. Population growth remains the largest contributing factor, contributing 53.00% globally, with 53.03% in females and 52.76% in males, while epidemiological change accounts for 21.09%, with 20.02% in females and 20.64% in males (Figure 5 and Supplementary Tables 7–9).

### 3.7 Advanced analysis of gout burden

**FIGURE 6:**
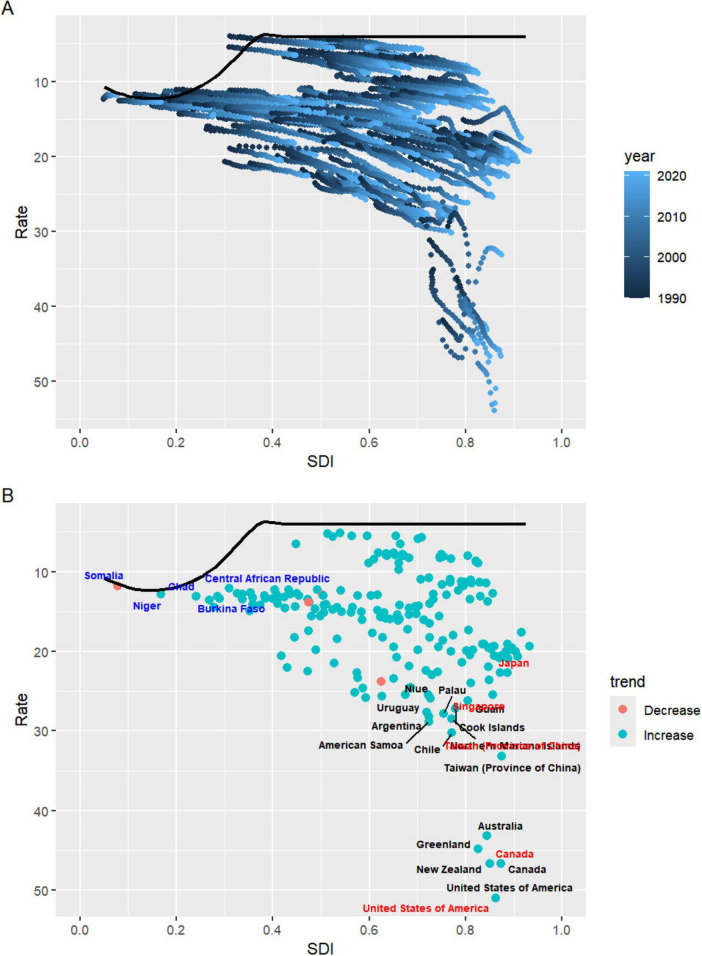
**(A)** Advanced analysis based on SDI and gout DALYs rate from 1990 to 2021. **(B)** Advanced analysis based on SDI and gout DALYs rate in 2021. DALYs, disability-adjusted life years; SDI, sociodemographic index.

Based on the 2021 GBD data, the Effective Difference quantifies the gap between a country’s observed disease burden, measured in DALYs, and the lowest disease burden that could potentially be achieved, referred to as the “frontier”. This frontier is defined by countries or regions with the highest SDI, which reflects the best possible health outcomes given favorable socioeconomic conditions. Countries with the largest Effective Difference, such as the United States (46.88), New Zealand (42.67), Canada (42.58), Greenland (40.71), and Australia (39.04), are significantly farther from the frontier, indicating that despite their relatively high SDI, there remains substantial room for improvement in reducing the burden of diseases. These high values suggest unrealized opportunities for better health outcomes through targeted interventions and policy reforms. On the other end of the spectrum, countries like Somalia (0.02), the Central African Republic (0.99), Guatemala (1.07), Niger (1.07), and Honduras (1.20) exhibit the smallest Effective Difference. These nations, despite facing varying levels of socioeconomic challenges, have managed to achieve disease burdens that are relatively close to the frontier. As a result, their potential for further reduction in disease burden is more limited compared to countries with larger disparities (Figure 6 and Supplementary Table 10).

### 3.8 Prediction analysis of gout burden

**FIGURE 7:**
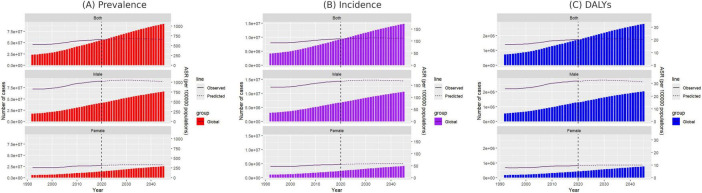
Projected number of new gout cases by gender in 2045 based on the SDI. **(A)** ASPR, **(B)** ASIR, and **(C)** ASDR. ASPR, age-standardized prevalence rate; ASIR, age-standardized incidence rate; ASDR, age-standardized DALY rate; SDI, sociodemographic index.

According to predictions, the global ASPR, ASIR, and ASDR for gout are expected to remain relatively stable over time, with projected rates by 2045 of 661.14, 111.72, and 20.36, respectively. However, when compared to 2021, notable differences are expected in ASPR trends between males and females. The ASPR for males is projected to decrease by 0.45%, while for females it is expected to increase by 4.30% (Figure 7 and Supplementary Table 11).

For females aged 15–29, the ASPR, ASIR, and ASDR are anticipated to follow a continuous upward trend by 2045, while females aged 30–59 are expected to experience a decline, with the most significant decrease in ASPR observed in the 50–54 age group. In contrast, for females over 60, ASPR is projected to rise progressively with age, with the largest increase of 19.21% occurring in those aged 95 and above (Supplementary Tables 12–14). Similarly, for males, only those aged 30–64 are expected to experience a continuous decline in ASPR, ASIR, and ASDR, while all other age groups are predicted to see an increase. The most significant decrease in ASPR is projected in males aged 50–54, whereas males aged 65 and above will likely see progressive increases in ASPR, with the 95-plus age group expected to experience the most notable rise at 21.72% (Supplementary Tables 15–17).

### 3.9 Cross-national inequalities in the burden of gout

**FIGURE 8:**
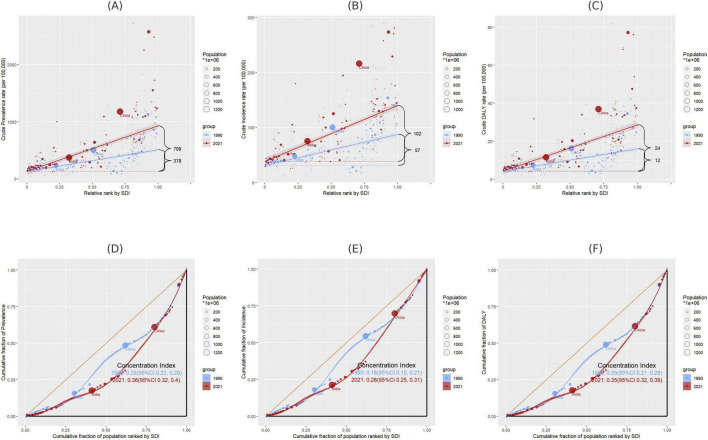
**(A–C)** Disparities in gout prevalence, incidence, and DALYs by SDI in 1990 and 2021, as measured by SII; **(D–F)** disparities in gout prevalence, incidence, and DALYs by SDI in 1990 and 2021, as measured by HCI. SII, Slope Index of Inequality; HCI, Health Concentration Index.

Within the analyzed 204 countries, profound and relative inequalities associated with the SDI in relation to gout can be observed, whereby high SDI nations bear a disproportionately higher burden. As indicated by the Slope Index of Inequality (SII), the disparity in prevalence between the highest and lowest SDI countries in 1990 and 2021 amounted to 378 (95% CI: 304 to 452) and 799 (95% CI: 698 to 908), respectively. The discrepancy in incidence was 57 (95% CI: 47 to 67) and 102 (95% CI: 88 to 116), while the DALY disparity was 12 (95% CI: 10 to 14) and 24 (95% CI: 21 to 28) (Figures 8A–C and Supplementary Table 18). In contrast, the relative inequality analysis showed that the Health Concentration Index (HCI) of prevalence was 0.25 (95% CI, 0.22 to 0.29) in 1990 and 0.36 (95% CI, 0.32 to 0.40) in 2021. The healthy concentration index of incidence was 0.18 (95% CI, 0.15 to 0.21) in 1990 and 0.28 (95% CI, 0.25 to 0.31) in 2021. The healthy concentration index for DALY was 0.25 (95% CI, 0.21 to 0.28) in 1990 and 0.35 (95% CI, 0.32 to 0.39) in 2021 (Figures 8D–F and Supplementary Table 18).

### 3.10 Gout-related DALYs attributable risk factors

**FIGURE 9:**
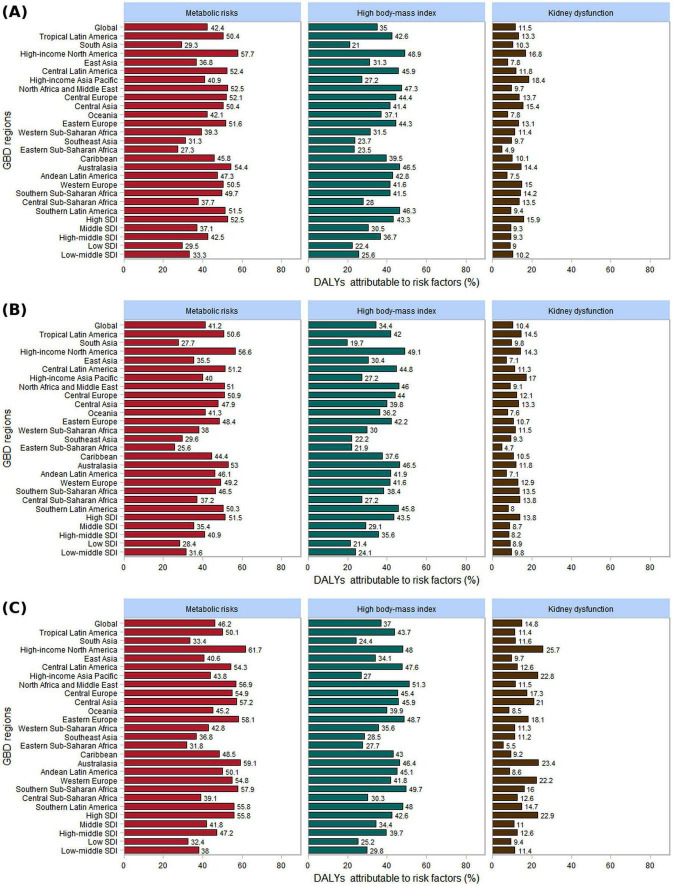
The three risk factors contributing to gout-related DALYs by gender in 2021, globally, across 21 GBD regions, and within 5 SDI regions. **(A)** Both; **(B)** male; **(C)** female.

The GBD 2021 database categorizes risk factors into four levels: Level 1 (3 factors), Level 2 (20 factors), Level 3 (42 factors), and Level 4 (22 factors) (Supplementary Table 19). The proportion of gout-related DALYs attributable to specific risk factors in 2021 varied across different Global Burden of Disease regions. Globally, in 2021, metabolic risk factors accounted for the largest share (42.4%), followed by high BMI (35.0%) and kidney dysfunction (11.5%) (Figure 9). Notably, the contribution of these three risk factors to gout-related DALYs was higher in females.

In 21 GBD regions, the highest burden of gout-related high BMI in 2021 was observed in High-income North America (48.9%). For men, the region with the high BMI burden related to gout was High-income North America (49.1%), while for women, it was North Africa and the Middle East (51.3%). Similarly, the highest burden of gout-related kidney dysfunction was found in High-income Asia Pacific (18.4%). For men, High-income Asia Pacific had the highest burden (17.0%), whereas for women, the greatest burden was in High-income North America (25.7%). In the 5 SDI regions, the burden of gout related to high BMI and kidney dysfunction was highest in the high SDI regions for both men and women. As the SDI level increased, the proportion of gout burden attributable to high BMI also rose (Figure 9).

## 4 Discussion

From a global perspective, between 1990 and 2021, the burden of gout among men has consistently been significantly higher than that of women, except in the age group of 95 years and above. Men’s lifestyle choices tend to increase the risk of developing gout. First, men generally consume more purine-rich foods, such as red meat and seafood, which can elevate uric acid levels in the body (35). Additionally, men typically consume more alcohol than women, particularly beer, which contains purines, and alcohol metabolism produces lactic acid, both of which inhibit uric acid excretion (36). The mechanisms by which alcohol leads to hyperuricemia and gout primarily involve reduced uric acid excretion and increased uric acid production. Alcohol metabolism generates lactic acid, which competitively inhibits the renal tubules’ ability to secrete uric acid, thus reducing its excretion (37, 38). Furthermore, alcohol consumption promotes the breakdown of adenosine triphosphate (ATP) into adenosine monophosphate (AMP), increasing the production of uric acid precursors, a process linked to the conversion of acetate into acetyl-CoA during ethanol metabolism (39, 40). These mechanisms work in tandem to explain how alcohol contributes to hyperuricemia and the development of gout. In addition, men exhibit lower adherence to preventive healthcare measures and tend to pay less attention to their health compared to women, a phenomenon supported by multiple studies. Men often display lower levels of medical adherence and health awareness, partly due to gender role expectations in society and culture. Many men perceive seeking medical help as a sign of weakness or a challenge to traditional notions of masculinity, leading them to neglect health issues and delay the prevention and treatment of diseases (41, 42). This behavior often results in the under-treatment of early symptoms of hyperuricemia and gout, exacerbating the disease burden. In males, uric acid production is generally higher compared to females, largely due to the protective role estrogen plays in females, particularly before menopause. Estrogen enhances renal uric acid excretion, thereby lowering serum uric acid levels and reducing the risk of developing hyperuricemia and subsequent gout in premenopausal women. Conversely, in men, the absence of estrogen leads to higher uric acid levels, increasing the likelihood of urate crystal formation, which can trigger gout. Estrogen’s protective effect may thus be attributed to its role in promoting the renal clearance of uric acid, mitigating the risk of hyperuricemia and gout (43–45).

Gout burden shows distinct patterns across different life stages and between genders. In adolescents and young adults, the incidence and prevalence of gout gradually increase, with men experiencing a more significant rise. This trend continues until the peak age of 55–59 years in men, where gout prevalence is most pronounced. In contrast, women generally exhibit a lower risk of gout before menopause, largely due to the protective effects of estrogen, which helps lower uric acid levels. However, after menopause, the decline in estrogen results in a loss of this protective effect, leading to a gradual increase in gout burden among women. Despite this postmenopausal rise, the overall incidence and prevalence of gout in women remain lower than in men of the same age. In elderly women, particularly those aged 95 and above, the prevalence of gout increases further, driven by longer life expectancy and a heightened burden of metabolic and chronic conditions, such as hypertension, diabetes, and chronic kidney disease (CKD). The postmenopausal reduction in estrogen exacerbates hyperuricemia, increasing the risk of gout. This condition not only causes joint inflammation but also worsens renal dysfunction and cardiovascular comorbidities. Studies underscore the independent role of gout in contributing to these chronic diseases, even after adjusting for common risk factors. The formation of monosodium urate (MSU) crystals triggers inflammatory responses that further damage renal function, highlighting the need for comprehensive management strategies that address both metabolic and inflammatory pathways in the treatment of gout in elderly women (46, 47).

The pathogenesis of gout is closely linked to dietary habits, as diet plays a significant role in influencing uric acid levels in the body. Dietary factors contribute to both the onset and exacerbation of gout, particularly through the consumption of purine-rich foods, alcohol, and specific micronutrients. Purines are naturally occurring substances found in many foods, especially animal-based products. When purines are metabolized, they break down into uric acid. Elevated uric acid levels can lead to the formation of urate crystals in the joints, which triggers the inflammation characteristic of gout. Therefore, a diet rich in purines is a major risk factor for the development of gout (48, 49). Foods that are high in purines include red meat, organ meats (such as liver and kidney), seafood (especially shellfish, sardines, and anchovies), and certain alcoholic beverages like beer. These foods contribute to an increase in uric acid levels, making them key dietary considerations in gout management (50, 51). For example, studies have shown that excessive consumption of red meat and organ meats significantly increases the risk of gout attacks, particularly in individuals who have pre-existing hyperuricemia (elevated uric acid levels)(52, 53). On the other hand, diets that are low in purines have been associated with a reduced risk of gout and its exacerbations. A plant-based diet, rich in fruits, vegetables, whole grains, and legumes, provides a lower purine load while offering a variety of beneficial nutrients such as antioxidants, fiber, and essential vitamins (54, 55). For instance, the Mediterranean diet, known for its high intake of vegetables, fruits, legumes, nuts, and olive oil, is typically low in purines and has been shown to offer protective effects against gout and rheumatoid arthritis disease (56, 57). The consumption of plant-based foods not only reduces purine intake but also provides anti-inflammatory benefits that can help mitigate the severity of gout attacks (58, 59). Several studies have demonstrated that dietary patterns emphasizing plant-based foods lead to a significant reduction in uric acid levels and the frequency of gout flares (55, 60). Furthermore, the Mediterranean diet has been shown to have additional benefits in terms of cardiovascular health, which is particularly important as gout patients are often at increased risk of cardiovascular diseases (61, 62).

Micronutrients, particularly vitamins, play an important role in the prevention and management of gout. Several studies suggest that vitamin C intake is inversely related to serum uric acid levels, meaning that higher vitamin C intake is associated with lower uric acid levels and a reduced risk of gout. Vitamin C is thought to reduce uric acid levels by increasing the excretion of uric acid through the kidneys (63–65). The study found that higher serum 25-hydroxyvitamin D (25(OH)D) levels are associated with a lower risk of mortality in gout patients. Specifically, patients with 25(OH)D levels ≥45 nmol/L had a significantly reduced likelihood of all-cause mortality (HR 0.72; 95% CI: 0.61–0.86) compared to those with levels <45 nmol/L (66). This study confirmed a positive correlation between hyperuricemia, gout, and increased risk of vitamin D deficiency. The analysis showed a dose-response relationship between uric acid levels and vitamin D deficiency risk, with a threshold at 307.5 μmol/L. Mendelian randomization analysis supported a causal association between gout and vitamin D deficiency. These findings highlight the strong link between gout and vitamin D deficiency (67). This study found negative associations between serum vitamin B12 and urate levels, as well as between serum folate and the risk of gout. Specifically, higher levels of vitamin B12 were associated with lower urate levels, and higher folate levels were associated with a reduced risk of gout. Conversely, positive associations were observed between serum calcium levels and both urate levels and the risk of gout. These findings suggest that vitamin B12, folate, and calcium may play important roles in regulating urate levels and influencing gout risk (68).

Metabolic risk factors such as obesity, hypertension, and diabetes are the primary drivers of gout burden, accounting for 42.4% of gout-related DALYs globally (69). This association highlights the central role of metabolic abnormalities in the pathogenesis of gout, indicating that targeted interventions aimed at addressing metabolic syndrome could substantially reduce the disease burden. The impact of high BMI is particularly pronounced in high-SDI regions, suggesting that the prevalence of gout may further worsen with socioeconomic development and lifestyle changes. Despite relatively abundant healthcare resources in these high-SDI regions, the prevalence of obesity and metabolic disorders poses a significant challenge.

Metabolic factors play a pivotal role in the onset and progression of gout, particularly through their contributions to high BMI, hyperglycemia, hypertension, and lipid metabolism disorders. The interaction of these factors creates a state of “metabolic imbalance”, which significantly increases the risk of gout (70). Obesity is closely linked to hyperuricemia and gout, as it induces a range of metabolic abnormalities, including insulin resistance, lipid metabolism disorders, and a chronic inflammatory state. These abnormalities increase uric acid production while reducing its excretion, thereby elevating serum uric acid levels. In individuals with obesity, the release of free fatty acids from adipose tissue during metabolism further impairs renal uric acid excretion (11, 71). The relationship between insulin resistance and gout is particularly complex. Insulin resistance, a hallmark of metabolic syndrome, has a bidirectional relationship with gout. On one hand, insulin resistance can promote hyperuricemia through various pathways. Increased insulin levels in the state of insulin resistance not only enhance uric acid production but also inhibit its excretion by renal tubules, leading to elevated serum uric acid levels. Conversely, hyperuricemia can exacerbate insulin resistance, creating a vicious cycle (72). Therefore, controlling insulin resistance is crucial in reducing uric acid levels and preventing gout flares (73). The link between hypertension and gout is reflected in two key aspects. First, patients with hypertension often have concurrent renal dysfunction, which affects uric acid excretion. Second, certain antihypertensive medications, such as thiazide diuretics, can increase uric acid reabsorption, leading to hyperuricemia (74, 75). Consequently, blood pressure management is an essential component of comprehensive gout treatment, especially for patients with coexisting metabolic syndrome (76). Hypercholesterolemia and hypertriglyceridemia, as components of metabolic syndrome, are not only risk factors for cardiovascular diseases but also play a significant role in the occurrence of gout (77). Studies have shown a significant association between elevated triglyceride levels and gout incidence. This relationship may be mediated by the inflammatory state of adipose tissue and increased oxidative stress, which indirectly affect uric acid metabolism (78).

The contribution of renal dysfunction to the burden of gout is also noteworthy, with its impact being particularly significant in high-income regions, likely due to the higher prevalence of chronic kidney disease in these areas. This highlights the critical importance of kidney health management in patients with gout, as renal dysfunction not only exacerbates hyperuricemia but can also limit the effectiveness of conventional gout treatments. Renal dysfunction plays a critical role in the development and progression of gout due to its impact on uric acid excretion. The kidneys are responsible for eliminating approximately two-thirds of the body’s uric acid load. When renal function declines, the kidney’ ability to excrete uric acid diminishes, resulting in an accumulation of uric acid in the bloodstream and subsequently increasing the risk of hyperuricemia and gout. Studies have shown that CKD is closely associated with elevated serum uric acid levels, which can not only trigger gout flares but also exacerbate renal impairment, forming a vicious cycle (79).

Moreover, renal dysfunction can affect the choice of medications used to manage gout, as certain drugs may have nephrotoxic effects or require dose adjustments in patients with compromised kidney function (80). Therefore, the management of gout in patients with renal dysfunction requires a careful balance to protect renal health while effectively controlling uric acid levels and preventing flares (81). In this context, monitoring and addressing kidney function is crucial to comprehensive gout treatment, particularly in patients with coexisting metabolic syndrome or cardiovascular comorbidities.

This study presents a novel approach to understanding the global burden of gout by utilizing decomposition analysis, advanced analysis, and cross-national inequality analysis—methodologies that are rarely employed together in gout research. By applying these tools, the study offers deeper insights into the key drivers of gout burden and highlights the potential for targeted interventions. Decomposition analysis uniquely dissects the contributions of population aging, growth, and epidemiological changes to the global burden of gout, providing a clear view of their relative importance. This is especially significant as it allows for a nuanced understanding of how these factors differ by gender and country, which is critical for designing region-specific health policies. The identification of population growth as the primary driver across all regions, with differential effects by gender, opens up opportunities for targeted interventions, particularly in countries with aging populations. Advanced analysis adds a new layer to this study by assessing the efficiency of gout burden reduction in different countries. By comparing each country’ssing the efficiency of gout burden reduction in differeanalysis reveals significant inefficiencies, even in high-SDI nations like the United States and New Zealand. These findings suggest that despite having advanced healthcare systems, these countries still have substantial room for improvement in reducing gout burden, challenging the assumption that higher socio-economic status automatically leads to better health outcomes. This insight underscores the need for healthcare reforms that focus on improving the efficiency and accessibility of gout care. Finally, cross-national inequality analysis highlights the growing disparities in gout burden between countries of varying socio-economic status. The increasing burden in high-SDI countries contrasts with the relatively stable burden in low-SDI nations, emphasizing that health outcomes are not solely determined by resources but also by the effectiveness of interventions and public health strategies. This finding calls for a deeper exploration of the social determinants of health and the development of tailored approaches to address regional disparities.

This study highlights the global burden of gout from 1990 to 2021, emphasizing cross-country disparities driven by socioeconomic factors. High-SDI regions face increasing challenges due to lifestyle-related risks, while low- and middle-SDI regions may have an underestimated burden due to healthcare limitations. The study calls for tailored interventions to address regional and demographic differences, including gender-sensitive strategies for postmenopausal women. It stresses the need for global health strategies to reduce disparities, improve healthcare access, and calls for further research on the interactions between socioeconomic factors and healthcare systems.

## Data Availability

The original contributions presented in this study are included in this article/Supplementary material, further inquiries can be directed to the corresponding author.

## Footnotes

1
https://vizhub.healthdata.org/gbd-results/
