# Supplementary material for: Assessing cross-national inequalities and predictive trends in gout burden: a global perspective (1990–2021)
Source: Front Med (Lausanne). 2025 Mar 27;12:1527716. doi: 10.3389/fmed.2025.1527716 (PMC11983511; doi:10.3389/fmed.2025.1527716)
Supplement: Supplementary file 1 [file Data_Sheet_1.pdf]

Supplementary methods

Calculation of ASR

$$\mathrm{ASR} = \frac{\left(\sum_{i=1}^{A} \mathrm{a}_i \mathrm{w}_i\right)}{\left(\sum_{i=1}^{A} w_i\right)} / \times 100{,}000$$

In this formula, i represents the i-th age group. The term a_i denotes the crude rate of the specific disease within the i-th age group, while w_i refers to the weight assigned to the i-th age group based on a reference standard population. The weight w_i reflects the proportion of individuals in that age group within the reference population, ensuring that the age distribution is standardized across different populations.

Decomposition analysis

We first used the decomposition methodology of Das Gupta to decompose NASH-associated liver cancer deaths by population age structure, population growth, and epidemiologic changes[1-3]. The number of deaths at each location was obtained from the following formula:

$$\mathrm{DALY}_{ay,\,py,\,ey} = \sum_{i=1}^{17} (a_{i,\,y} * p_{y} * e_{i,\,y})$$

Where $\mathrm{DALY}_{ay,\,py,\,ey}$ represented DALYs based on the factors of age structure, population, and DALYs rate for specific year y; $a_{i\ y}$ represents the proportion of population for the age category i of the 17 age categories in given year y; p_y represents the total population in given year y; and $e_{i,\,y}$ represents DALYs rate given age category i in year y. The contribution of each factor to the change in DALYs from 1990 to 2021 was defined by the effect of one factor changing while the other factors were held constant. For example, the effect of age structure was calculated as:

$(\mathrm{DALY}_{a2021,\,p1990,\,e1990} + \mathrm{DALY}_{a2021,\,p2021,\,e2021})/3 + (\mathrm{DALY}_{a2021,\,p1990,\,e2019} + \mathrm{DALY}_{a2021,\,p2021,\,e1990})/6] - [(\mathrm{DALY}_{a1990,\,p2021,\,e2021} + \mathrm{DALY}_{a1990,\,p1990,\,e1990})/3 + (\mathrm{DALY}_{a1990,\,p2021b,\,e1990} + \mathrm{DALY}_{a1990,\,p1990,\,e2021})/6$

Frontier analysis

In order to evaluate the relationship between the burden of NASH-associated liver cancer and socio-demographic development, we applied a frontier analysis as a quantitative methodology to identify the lowest potentially achievable

age-standardized death rate based on development status, as measured by the SDI. The death rate frontier pinpoints the minimum number of deaths that could be attained for every country or territory given its SDI. Distance from the frontier is termed the effective difference; a large effective difference from the frontier suggests there may be unrealized opportunities for gains or improvement (reduction in NASH-associated liver cancer deaths) that should be possible based on the country or territory's place on the development spectrum. A data envelopment analysis, which allows for the delineation of non-linear frontiers, utilizing the free disposal hull method, was developed to produce a frontier for age-adjusted NASH-associated liver cancer deaths by SDI, using data from 1990-2021. In order to account for uncertainty, we used 1,000 bootstrapped samples of the data, randomly sampling with replacement from all countries and territories across all years. The mean death rate for NASH-associated liver cancer at each SDI value from the bootstrapped samples was computed. A LOESS regression with local polynomial degree of 1 and span of 0.2 was then developed to generate a smoothed frontier. To exclude the influence of outliers, super-efficient countries were excluded from the generation of the frontier. To understand the relationship between age-standardized NASH-associated liver cancer death rates relative to the frontier in 2021, we calculated the effective difference (the absolute distance from the frontier) using 2021 SDI and age-standardized death rate data points for each country or territory. Countries or territories with lower death rates than the frontier were assigned a zero distance.

1. Das Gupta P: **Standardization and decomposition of rates from cross-classified data**. *Genus* 1994, **50**(3-4):171-196.
2. Chevan A, Sutherland M: **Revisiting Das Gupta: refinement and extension of standardization and decomposition**. *Demography* 2009, **46**(3):429-449.
3. Gupta PD: **Standardization and decomposition of rates: a user's manual**; 1993.

Table S1 Terms and definitions

The Global Burden of Disease (GBD): GBD is a comprehensive epidemiological database spearheaded by the Institute for Health Metrics and Evaluation (IHME) at the University of Washington. It covers 288 causes of death, 371 diseases and injuries, and 88 risk factors across 204 countries and territories, including subnational estimates for 21 regions, spanning the years 1990 to 2021. The GBD provides detailed insights into global health trends and emerging challenges. With its vast array of data sources and advanced statistical modeling methods, the GBD database offers the most comprehensive estimates of disease burden, including the burden of nonalcoholic steatohepatitis (NASH)-associated liver cancer. This study utilized the most recent GBD dataset to evaluate the global burden of NASH-associated liver cancer, providing updated and in-depth assessments of this pressing public health issue.

Age-standardized rate: Rate per 100,000 population following standardization to the global age structure. The difference between age-standardized rates across geographies and over time is independent of population size and age structure.

The Sociodemographic Index (SDI): SDI is a composite measure that quantifies the level of sociodemographic development of a country or region. It is comparable across geographies and over time. The SDI is expressed on a scale from 0 to 1 and is calculated as the composite average of rankings for per capita income, average educational attainment, and total fertility rates across all areas included in the GBD study. A value of 0 represents the lowest income per capita, lowest educational attainment, and highest fertility rate observed across all GBD geographies from 1970 to 2021, while a value of 1 represents the highest income per capita, highest educational attainment, and lowest fertility rate.

Decomposition analysis: An analysis approach invented by Das Gupta, which summarize the contribution of various factors to the observed changes by algebraic isolation of the standardized impact of each contribution multiplication factor. Decomposing the epidemiological indicators of disease according to age structure, population growth and epidemiologic changes can quantify the contribution of each of these factors to the overall effect.

Frontier analysis: frontier analysis is an analytic approach used to identify the lowest potentially achievable burden of NASH-associated liver cancer based on a country or region's level of development, as measured by the SDI. The frontier represents those countries or territories that are at the forefront of performance, achieving the lowest burden of NASH-associated liver cancer for their respective SDI. The distance from this frontier, termed the “effective difference,” reflects the gap between the observed burden and the theoretically achievable burden of disease for a country or region, given its SDI. This gap suggests an opportunity to reduce or eliminate the excess burden of disease based on the country or region’s sociodemographic resources. For instance, if a country or territory falls significantly below the frontier for its SDI, this indicates an unrealized potential for reducing NASH-associated liver cancer deaths, which could be addressed through better utilization of available resources aligned with its development status.

Confidence interval (CI) and uncertainty intervals (UIs): Because the mean value of the real

population is unknown, the range with upper and lower limits is calculated according to the samples to describe the possible value of the mean value, which is the mean CI. We used the 25th and 975th ordinal 1000 draw values of the posterior distribution to generate UIs.

Table S2. Gout Prevalence in 204 countries and territories between 1990 and 2021.

Location	Prevalence case No. (95% UI)		ASPR per 100,000 No. (95% UI)		1990-2021EAPC in ASPR No. (95% CI)
	1990	2021	1990	2021	
Afghanistan	26617 (20449,34171)	52184 (40277,66211)	389.03 (308.6,491.7)	427.33 (337.99,532.82)	0.39 (0.30, 0.49)
Albania	6920 (5482,8811)	14269 (11255,17998)	306.73 (245.37,389.09)	351.02 (280.26,439.38)	0.47 (0.45, 0.49)
Algeria	56774 (44748,72172)	201008 (159289,250888)	433.3 (344.03,544.1)	523.92 (420.59,644.16)	0.67 (0.65, 0.69)
American Samoa	226 (177,288)	471 (368,607)	850.69 (669.37,1069.9)	942.54 (750.82,1183.7)	0.25 (0.22, 0.28)
Andorra	370 (287,471)	986 (776,1267)	623.02 (483.97,785.22)	668.05 (531.37,853.45)	0.21 (0.18, 0.23)
Angola	18116 (14208,23517)	59581 (46704,76251)	407.75 (326.18,512.8)	426.63 (341.09,532.5)	0.17 (0.14, 0.21)
Antigua and Barbuda	100 (81,125)	271 (215,341)	189.24 (150.69,236.44)	250.37 (200.43,311.02)	0.93 (0.90, 0.96)
Argentina	237493 (187356,297416)	491574 (389793,620055)	737.34 (583.16,918.48)	909.13 (720.15,1141.34)	0.64 (0.60, 0.68)
Armenia	10646 (8443,13724)	18921 (14801,23858)	372.66 (297.01,469.99)	456.81 (362.42,574.13)	0.77 (0.72, 0.82)
Australia	196110 (155517,246428)	581486 (449730,748493)	1014.31 (812.06,1270.66)	1405.38 (1106.19,1783.71)	1.17 (1.06, 1.28)
Austria	57247 (45260,72810)	100313 (78899,128072)	529.7 (420.12,672.8)	633.67 (501.5,800.64)	0.58 (0.55, 0.60)
Azerbaijan	19703 (15490,25086)	50823 (39780,65008)	371.32 (295.62,464.88)	459.49 (364.21,578.15)	0.80 (0.74, 0.85)
Bahamas	369 (295,460)	1088 (857,1399)	203.67 (163.21,253.43)	253.85 (204.27,320.87)	0.73 (0.69, 0.76)
Bahrain	1345 (1045,1714)	8142 (6259,10510)	509.04 (404.34,636.08)	609.47 (481.45,766.46)	0.63 (0.60, 0.66)
Bangladesh	194806 (156462,242621)	572674 (456649,725675)	376.26 (300.72,470.28)	397.87 (318.68,511.61)	0.20 (0.19, 0.21)
Barbados	542 (440,677)	1162 (923,1467)	201.95 (162.52,252.49)	256.34 (204.08,319.84)	0.78 (0.75, 0.80)
Belarus	44580 (35451,56296)	60147 (47565,76630)	353.45 (281.16,442.78)	401.35 (318.76,504.15)	0.43 (0.40, 0.46)
Belgium	77457 (61330,97913)	125332 (98847,156252)	546.56 (434.93,688.51)	627.11 (491.52,791.94)	0.45 (0.43, 0.47)
Belize	207 (168,258)	926 (727,1164)	200.09 (161.95,249.77)	270.45 (214.99,338.51)	0.96 (0.89, 1.04)
Benin	8746 (6973,10907)	27749 (21980,34809)	406.18	458.89	0.41 (0.39,

			(322.89,508.56)	(361.82,576.6)	0.42)
Bermuda	141 (112,179)	307 (245,387)	215.66 (171.39,273.05)	273.24 (218.16,341.67)	0.80 (0.78, 0.82)
Bhutan	1058 (835,1345)	2821 (2233,3529)	373.8 (297.79,469.13)	434.84 (346.42,545.29)	0.52 (0.49, 0.54)
Bolivia (Plurinational State of)	7829 (6203,9873)	28275 (22372,35062)	211.69 (169.52,267.74)	282.66 (226.13,348.93)	0.95 (0.91, 0.98)
Bosnia and Herzegovina	12619 (9884,15978)	19875 (15475,25566)	292.84 (233.65,365.95)	352.04 (277.78,448.1)	0.61 (0.59, 0.62)
Botswana	2659 (2091,3316)	8962 (7022,11172)	433.41 (343.37,541.38)	523.09 (414.96,658.88)	0.61 (0.56, 0.66)
Brazil	203435 (161476,253747)	648012 (519467,814679)	201.02 (161.4,251.02)	255.36 (205.69,318.19)	0.81 (0.78, 0.84)
Brunei Darussalam	909 (713,1148)	3202 (2478,4067)	684.92 (535.57,862.7)	773.78 (608.07,970.1)	0.41 (0.38, 0.44)
Bulgaria	39012 (30544,49550)	44608 (34931,56286)	330 (265.14,411.49)	355.25 (283.07,444.37)	0.24 (0.21, 0.28)
Burkina Faso	17570 (13837,22281)	44394 (34950,56844)	382.62 (304.19,479.79)	421.38 (334.2,528.95)	0.34 (0.33, 0.35)
Burundi	10034 (8010,12678)	24758 (19550,30935)	397.85 (316.67,496.32)	431.44 (341.05,540.67)	0.31 (0.30, 0.33)
Cabo Verde	849 (681,1066)	2249 (1790,2827)	381.38 (302.68,477.75)	463.63 (369.15,581.02)	0.69 (0.65, 0.72)
Cambodia	24606 (19365,31176)	77505 (60487,99976)	478.35 (377.94,604.25)	559 (442.07,703.18)	0.54 (0.50, 0.57)
Cameroon	22285 (17601,27998)	75414 (59839,94918)	454.67 (361.94,567.77)	504.42 (398.35,631.25)	0.35 (0.33, 0.37)
Canada	367368 (296808,461592)	929457 (740788,1193830)	1157.36 (928.56,1453.89)	1499.53 (1198.53,1906.37)	0.83 (0.78, 0.88)
Central African Republic	4985 (3845,6473)	10520 (8213,13524)	392.71 (306.61,496.29)	398.16 (315.63,497.54)	0.07 (0.05, 0.08)
Chad	11315 (8892,14194)	28275 (22314,35456)	380.76 (302.06,477.24)	425.81 (337.41,531.75)	0.41 (0.40, 0.42)
Chile	83048 (65839,104717)	242741 (191720,306995)	785.33 (624.86,994.49)	981.62 (771.67,1236.74)	0.67 (0.61, 0.74)
China	5971855 (4746980,7526175)	16788147 (13144119,21277610)	640.68 (512.12,796.74)	810.36 (644.76,1009.05)	1.08 (0.95, 1.20)
Colombia	28676 (22697,36044)	94553 (75810,118794)	132.23 (104.98,165.12)	171.74 (138.34,216.87)	0.81 (0.79, 0.84)
Comoros	911 (712,1164)	2378 (1885,2991)	425.92 (337.86,535.65)	446.58 (355.71,558.17)	0.18 (0.16, 0.19)
Congo	4776 (3724,6148)	15601 (12219,19997)	415.95 (326.42,526.81)	490.21 (383.73,611.83)	0.57 (0.54, 0.59)

Cook Islands	105 (83,133)	216 (170,275)	783.17 (620.08,983.81)	884.27 (700.46,1111.59)	0.32 (0.29, 0.35)
Costa Rica	3038 (2424,3810)	10071 (8012,12602)	146.8 (116.86,182.72)	184.02 (146.98,229.11)	0.70 (0.68, 0.72)
Croatia	18707 (14790,23878)	28376 (22331,35744)	308.8 (247.65,391.72)	367.94 (288.45,462.56)	0.24 (0.21, 0.27)
Cuba	20956 (16873,26205)	44315 (35287,55893)	201.66 (163.21,251.97)	254.09 (203.96,316.2)	0.60 (0.58, 0.63)
Cyprus	4448 (3506,5667)	12134 (9570,15503)	539.77 (427.83,683.18)	608.07 (477.36,771.84)	0.79 (0.76, 0.82)
Czechia	40886 (32637,51443)	69389 (54509,88149)	310.44 (248.68,388.95)	367.98 (292.13,465.27)	0.36 (0.32, 0.41)
Côte d'Ivoire	21168 (16610,26867)	64201 (50239,81287)	440.17 (346.25,547.66)	472.06 (374.87,593.02)	0.68 (0.48, 0.88)
Democratic People's Republic of Korea	122865 (95672,155616)	264438 (209097,332478)	690.83 (549.73,862.77)	792.98 (631,982.69)	0.46 (0.43, 0.50)
Democratic Republic of the Congo	72191 (56559,93264)	183016 (144613,233962)	427.16 (339.5,544.32)	423.41 (337.18,534.02)	-0.04 (-0.12, 0.04)
Denmark	39803 (31725,50158)	64113 (50107,82604)	539.46 (430.43,676.36)	626.61 (496.29,793.35)	0.52 (0.49, 0.55)
Djibouti	701 (550,886)	3640 (2842,4690)	415.7 (329.17,518.56)	470.49 (372.32,596.55)	0.46 (0.43, 0.48)
Dominica	114 (91,143)	214 (170,271)	197.53 (157.32,247.67)	264.34 (210.52,331.85)	0.94 (0.90, 0.97)
Dominican Republic	7907 (6320,9852)	25682 (20703,31913)	184.63 (148.21,230.12)	244.44 (197.08,304.31)	0.98 (0.94, 1.02)
Ecuador	14583 (11570,18195)	52727 (42174,66110)	239.71 (192.22,299.52)	310.76 (249.77,390.18)	1.11 (1.01, 1.21)
Egypt	136781 (107421,173987)	403829 (314380,514803)	453.66 (361.02,566.33)	575.01 (454.36,718.32)	0.71 (0.69, 0.73)
El Salvador	4437 (3549,5506)	10754 (8647,13423)	132.33 (106,164.92)	173.09 (138.78,215.56)	0.84 (0.80, 0.87)
Equatorial Guinea	800 (631,1032)	3227 (2542,4101)	385.98 (309.04,489.05)	502.41 (390.76,628.12)	1.07 (0.98, 1.15)
Eritrea	5128 (3965,6617)	13592 (10686,17053)	375.48 (296.21,466.58)	400.77 (319.59,503.13)	0.20 (0.17, 0.23)
Estonia	7245 (5771,9114)	10221 (8018,12984)	365.18 (290.06,458.43)	443.87 (347.57,564.42)	0.68 (0.66, 0.70)
Eswatini	1621 (1276,2092)	3476 (2715,4400)	492.96 (390.06,631.59)	530.04 (418.01,660.98)	0.18 (0.15, 0.21)
Ethiopia	93410 (73675,118346)	227702 (182150,283934)	430.8 (344.71,537.76)	458.12 (365.45,568.12)	0.20 (0.19, 0.21)
Fiji	3100 (2463,3899)	6960 (5424,8766)	715.62 (572.73,893.09)	830.74 (660.17,1035.92)	0.41 (0.38, 0.43)

Finland	35714 (28126,45202)	65275 (50610,83540)	528.33 (412.63,666.81)	625.26 (490.98,788.11)	0.53 (0.50, 0.56)
France	406938 (324636,512102)	720168 (567783,922679)	532.38 (419.31,671.07)	614.87 (482.62,778.85)	0.46 (0.44, 0.49)
Gabon	2531 (1990,3182)	6061 (4790,7866)	429.79 (342.42,538.97)	518.65 (410.36,653.42)	0.61 (0.60, 0.63)
Gambia	1713 (1353,2156)	5123 (4100,6459)	422.58 (335.9,524.49)	452.69 (356.75,561.64)	0.22 (0.21, 0.23)
Georgia	23951 (18702,30593)	23618 (18660,29819)	387.77 (307.88,487.22)	432.43 (343.33,537.68)	0.38 (0.35, 0.42)
Germany	638645 (504773,805748)	1051530 (824550,1333181)	544.61 (430.65,684.68)	644.83 (509.61,816.52)	0.58 (0.56, 0.59)
Ghana	28027 (22077,35784)	90326 (71527,114016)	398.63 (318.61,495.11)	463.85 (368.91,582.4)	0.48 (0.46, 0.51)
Greece	89721 (70613,114398)	136427 (107378,171860)	622.95 (492.85,789.43)	687.37 (541.42,866.54)	0.64 (0.16, 1.12)
Greenland	482 (374,616)	1026 (798,1312)	1131.02 (895.01,1423.4)	1448.5 (1140.93,1816.6)	0.86 (0.81, 0.92)
Grenada	125 (103,156)	291 (225,368)	183.89 (148.16,230.39)	249.97 (197.2,313.38)	0.93 (0.89, 0.96)
Guam	691 (547,885)	1692 (1327,2141)	753.43 (600.35,956.74)	836.48 (658.38,1040.06)	0.34 (0.33, 0.36)
Guatemala	5353 (4176,6775)	20241 (16052,25523)	124.57 (98.72,157.38)	163.62 (130.14,207.54)	0.87 (0.85, 0.89)
Guinea	13966 (11052,17774)	27260 (21614,34218)	405.97 (323.52,512.51)	434.2 (340.82,550.35)	0.23 (0.21, 0.26)
Guinea-Bissau	1710 (1347,2195)	3601 (2818,4654)	393.53 (311.69,495.5)	407.94 (323.43,516.77)	0.12 (0.11, 0.13)
Guyana	859 (679,1086)	1684 (1335,2146)	189.1 (150.44,238.17)	245.5 (196.31,310.49)	0.80 (0.78, 0.82)
Haiti	6393 (5064,8017)	18238 (14292,22847)	172.41 (138.65,216.03)	206.95 (165.99,255.39)	0.62 (0.60, 0.63)
Honduras	3151 (2479,3933)	12384 (9732,15645)	126.7 (100.88,158.13)	165.13 (131.48,206.08)	0.84 (0.80, 0.88)
Hungary	44645 (35261,56000)	62435 (49284,78007)	318.59 (254.02,398.02)	363.53 (289.66,452.22)	0.41 (0.40, 0.41)
Iceland	1595 (1269,2034)	3461 (2735,4388)	584.69 (459.63,745.43)	661.15 (523.1,830.28)	0.41 (0.39, 0.44)
India	2012110 (1591668,2549676)	5315284 (4217470,6672708)	394.93 (317.02,493.19)	424.26 (340.6,530.02)	0.22 (0.19, 0.24)
Indonesia	603072 (477201,755944)	1789335 (1406173,2268336)	533.53 (429,664.02)	663.17 (529.97,827.12)	0.70 (0.68, 0.72)
Iran (Islamic Republic of)	126746 (98732,162130)	422052 (334241,533080)	442.07 (352.56,556.7)	500.09 (401.44,626.58)	0.37 (0.30, 0.44)

Iraq	41446 (33428,52096)	145241 (114092,183732)	473.09 (376.53,591.07)	525.6 (417.6,654.41)	0.34 (0.31, 0.38)
Ireland	22660 (17986,28593)	48314 (38153,61209)	581.39 (459.47,728.88)	657.94 (520.73,830.84)	0.41 (0.38, 0.43)
Israel	27164 (21596,34557)	74510 (58884,94114)	576.62 (455.76,729.22)	653.19 (515.37,825.34)	0.40 (0.38, 0.42)
Italy	413550 (327133,527594)	672246 (526847,856049)	500.8 (397.01,636.13)	556.2 (437.97,700.07)	0.42 (0.29, 0.55)
Jamaica	3536 (2848,4386)	8120 (6544,10164)	198.96 (159.24,247.81)	260.31 (208.69,325.79)	0.89 (0.83, 0.94)
Japan	1087073 (852172,1385885)	1985731 (1565687,2526078)	648.12 (510.95,818.37)	722.89 (568.23,917.04)	0.33 (0.32, 0.35)
Jordan	7533 (5922,9536)	52226 (41042,66425)	479.58 (379.2,607.43)	593.49 (474.99,745.12)	0.74 (0.71, 0.77)
Kazakhstan	50382 (39514,63123)	85431 (66754,107576)	380.02 (302.19,474.23)	453.42 (359,563.41)	0.62 (0.58, 0.66)
Kenya	41091 (33031,51259)	127193 (100987,161387)	450.89 (361.22,561.52)	477.93 (382,597.62)	0.23 (0.18, 0.28)
Kiribati	272 (215,343)	581 (452,742)	648.4 (518.32,814.02)	696.63 (554.66,876.19)	0.15 (0.13, 0.18)
Kuwait	5079 (3928,6478)	25806 (19961,33094)	568.67 (449.71,713.34)	638.31 (502.56,808.49)	0.52 (0.45, 0.58)
Kyrgyzstan	11234 (8940,14299)	22080 (17225,28102)	364.65 (289.01,458.39)	416.22 (330.09,519.67)	0.42 (0.41, 0.44)
Lao People's Democratic Republic	11799 (9220,14942)	33912 (26782,42627)	513.33 (406.37,634.17)	630.68 (502.97,787.68)	0.72 (0.69, 0.75)
Latvia	12590 (10010,15791)	14407 (11437,18326)	364.63 (290.92,455.09)	422.11 (339.61,532.03)	0.50 (0.48, 0.52)
Lebanon	9888 (7771,12576)	30785 (24654,38642)	435.21 (345.78,550.56)	511.98 (410.16,647.64)	0.54 (0.50, 0.58)
Lesotho	3541 (2799,4500)	5578 (4382,7148)	396.55 (314.49,501.47)	466.32 (367.55,589.15)	0.55 (0.52, 0.58)
Liberia	5538 (4416,6946)	12891 (10068,16299)	451.15 (357.69,565.62)	489.3 (388.19,612.67)	0.44 (0.35, 0.52)
Libya	10056 (8010,12667)	32922 (26194,41308)	478.93 (381.54,602.6)	539.46 (426.8,669.2)	0.41 (0.37, 0.46)
Lithuania	16281 (13024,20377)	20467 (16263,25748)	369.53 (294.27,460.55)	410.74 (325.95,517.58)	0.35 (0.33, 0.37)
Luxembourg	2888 (2284,3708)	6371 (5031,8062)	554.68 (437.57,708.84)	642.91 (504.19,810.76)	0.48 (0.47, 0.50)
Madagascar	22944 (18063,28859)	57392 (44704,72803)	420.47 (335.82,524.81)	431.73 (345.45,542.44)	0.13 (0.09, 0.16)
Malawi	17315 (13655,22035)	37317 (29528,48048)	409.22 (323.26,509.33)	431.05 (342.32,545.69)	0.19 (0.18, 0.20)

Malaysia	62497 (49544,78195)	220750 (173381,278300)	577.12 (458.76,716.8)	726.06 (574.11,908.46)	0.75 (0.74, 0.76)
Maldives	605 (469,771)	3491 (2723,4462)	596.09 (474.47,748.31)	743.79 (591.78,931.04)	0.74 (0.66, 0.81)
Mali	16958 (13449,21646)	43785 (34615,56249)	395.61 (316.58,494.74)	438.67 (351.07,555.1)	0.37 (0.34, 0.39)
Malta	2297 (1812,2908)	5251 (4085,6714)	539.05 (424.51,677.47)	634.75 (498.8,800.38)	0.49 (0.45, 0.53)
Marshall Islands	122 (97,155)	300 (233,388)	636.8 (509.97,789.38)	739.23 (586.98,930.99)	0.46 (0.44, 0.48)
Mauritania	4598 (3628,5806)	11882 (9429,15072)	429.97 (341.15,538.02)	502.47 (398.56,638.89)	0.50 (0.47, 0.52)
Mauritius	4771 (3775,5969)	12973 (10066,16480)	581.9 (463.13,724.53)	724.31 (570.99,906.66)	0.71 (0.70, 0.72)
Mexico	90031 (71055,113235)	275294 (216711,350018)	176.19 (140.05,221.36)	206.81 (164.21,260.63)	0.74 (0.63, 0.84)
Micronesia (Federated States of)	377 (303,469)	655 (511,833)	703.51 (563.38,881.4)	795.1 (629.06,992.03)	0.33 (0.31, 0.34)
Monaco	348 (272,447)	536 (412,688)	587.07 (461.11,736.63)	662.61 (524.35,833.4)	0.39 (0.36, 0.42)
Mongolia	4313 (3435,5405)	11035 (8640,13853)	380.6 (304.46,477.05)	415.67 (331.04,514.52)	0.34 (0.31, 0.37)
Montenegro	2142 (1685,2715)	3488 (2754,4441)	338.17 (268.5,424.38)	378.96 (302.9,473.68)	0.44 (0.40, 0.48)
Morocco	62248 (49053,78783)	177558 (139527,220621)	409.94 (324.4,510.6)	494.95 (393.66,613.47)	0.62 (0.59, 0.65)
Mozambique	26119 (20473,33272)	54254 (42723,67969)	400.18 (319.72,504.18)	416.84 (328.62,518.95)	0.18 (0.16, 0.20)
Myanmar	130020 (102206,167693)	308600 (243888,389420)	510.11 (404.65,643.42)	591.36 (473.77,734.5)	0.51 (0.47, 0.55)
Namibia	3139 (2458,3954)	7516 (5894,9582)	446.67 (353.99,554.76)	482.89 (379.42,599.14)	0.26 (0.23, 0.29)
Nauru	42 (33,52)	52 (41,67)	779.37 (618.55,986.13)	772.05 (608.04,968.32)	-0.09 (-0.19, 0.02)
Nepal	37720 (29721,47908)	97736 (77315,123330)	365.4 (290.53,456.72)	400.39 (319.75,503.32)	0.32 (0.31, 0.33)
Netherlands	110024 (87536,138405)	205534 (162522,263129)	580.22 (460.51,731.4)	670.21 (530.23,844.65)	0.20 (0.10, 0.30)
New Zealand	52498 (41324,66818)	117437 (92025,150595)	1370.05 (1087.11,1728.37)	1517.25 (1193.67,1925.54)	0.14 (-0.06, 0.34)
Nicaragua	2550 (2004,3235)	9901 (7867,12452)	130.82 (104.55,165.84)	175.24 (139.36,219.76)	0.92 (0.89, 0.96)
Niger	12965 (10223,16507)	38523 (30613,48325)	404.57 (321.13,508.12)	410.73 (329.31,510.73)	0.05 (0.04, 0.07)

Nigeria	212406 (168867,268729)	449640 (354849,565615)	440.84 (352.02,552.65)	416.99 (334.91,517.61)	-0.25 (-0.34, -0.15)
Niue	15 (12,19)	18 (14,22)	703.16 (559.5,879.14)	840.96 (662.87,1052.35)	0.55 (0.52, 0.58)
North Macedonia	6172 (4892,7777)	12081 (9395,15478)	322.04 (256.56,402.43)	374.89 (296.4,471.09)	0.52 (0.50, 0.53)
Northern Mariana Islands	240 (184,309)	514 (397,664)	910.38 (714.62,1144.84)	917.05 (724.75,1146.15)	-0.07 (-0.11, -0.04)
Norway	29472 (23276,37291)	49588 (38933,63023)	490.44 (388.75,618.52)	562.4 (442.45,710.37)	0.21 (-0.06, 0.48)
Oman	4022 (3130,5080)	18637 (14398,23597)	431.37 (344.33,544.48)	585.51 (462.4,730.68)	0.97 (0.92, 1.02)
Pakistan	218935 (173672,277220)	587226 (460336,754733)	364.25 (289.37,456.81)	425.42 (335.33,538.71)	0.65 (0.57, 0.73)
Palau	82 (65,104)	215 (167,278)	756.23 (598.16,948.23)	903.41 (719.04,1126.6)	0.46 (0.39, 0.53)
Palestine	3886 (3079,4856)	14860 (11679,19197)	415.56 (330.88,521.45)	494.91 (394,621.95)	0.49 (0.46, 0.52)
Panama	2212 (1761,2804)	7877 (6253,9871)	129.14 (103.03,163.39)	178.09 (141.43,223.52)	1.01 (0.99, 1.02)
Papua New Guinea	12958 (10018,16615)	40876 (32187,52020)	615.25 (486.1,771.58)	666.4 (533,839.3)	0.24 (0.23, 0.26)
Paraguay	4851 (3875,6036)	15458 (12414,19289)	193.54 (155.05,242.87)	244.1 (197.02,307.02)	0.75 (0.73, 0.76)
Peru	29192 (23412,36689)	99584 (79651,125059)	214.92 (171.9,271.01)	282.13 (225.87,353.9)	0.94 (0.92, 0.97)
Philippines	174771 (137538,221594)	570921 (446792,729953)	508.93 (409.46,638.03)	626.01 (499.18,784.43)	0.66 (0.60, 0.72)
Poland	139102 (110786,175691)	239411 (189365,300732)	323.26 (258.9,404.21)	371.4 (298.13,462.04)	0.43 (0.42, 0.44)
Portugal	72225 (56715,91517)	129852 (101327,164142)	543.62 (430.22,682.3)	624.97 (494.28,784.19)	0.54 (0.45, 0.63)
Puerto Rico	7871 (6315,9763)	16231 (13171,20035)	219.6 (176.73,273.08)	283.71 (226.98,350.36)	0.84 (0.81, 0.87)
Qatar	1394 (1042,1810)	15517 (11628,20452)	596.15 (468.57,757.7)	716.78 (571.79,898.81)	0.70 (0.65, 0.75)
Republic of Korea	207066 (160821,262199)	646718 (504289,826689)	607.71 (480.15,763.99)	728.59 (571.95,919.16)	0.58 (0.56, 0.59)
Republic of Moldova	15743 (12325,20179)	23251 (18371,29479)	356.28 (284.03,448.51)	411.37 (326.03,518.73)	0.54 (0.49, 0.58)
Romania	85001 (66755,107815)	120019 (94923,149926)	311.76 (249.39,390.6)	362.91 (287.31,453.22)	0.52 (0.50, 0.54)
Russian Federation	661947 (521024,836506)	987087 (775580,1246321)	372.33 (297.53,465.69)	439.04 (349.75,548.14)	0.55 (0.54, 0.56)

Rwanda	12312 (9686,15741)	30196 (23766,38048)	396.95 (314.27,502.11)	414.21 (327.36,513.88)	0.17 (0.11, 0.22)
Saint Kitts and Nevis	69 (55,86)	189 (148,244)	201.07 (161.03,252.5)	259.9 (208.03,326.9)	0.81 (0.77, 0.85)
Saint Lucia	175 (140,218)	599 (479,762)	191.06 (152.84,240.66)	255.15 (204.39,321.84)	0.91 (0.86, 0.96)
Saint Vincent and the Grenadines	136 (108,169)	351 (280,441)	184.91 (147.72,230.89)	253.98 (203.75,317.72)	1.06 (1.02, 1.10)
Samoa	702 (551,891)	1289 (1024,1622)	760.72 (598.77,951.54)	835.95 (670.35,1043.34)	0.21 (0.18, 0.24)
San Marino	190 (150,242)	408 (323,521)	585.09 (461.25,743.14)	639.21 (506.23,807.1)	0.28 (0.24, 0.32)
Sao Tome and Principe	273 (215,350)	633 (502,794)	411.5 (324.59,524.71)	486.53 (387.03,608.68)	0.57 (0.56, 0.59)
Saudi Arabia	35741 (28058,45599)	182224 (139129,233849)	488.62 (386.52,613.04)	654.21 (516.89,821.91)	0.92 (0.90, 0.95)
Senegal	14626 (11574,18593)	38018 (30101,47523)	418.46 (336.3,528.42)	440.37 (350.88,549.85)	0.17 (0.16, 0.18)
Serbia	36062 (27897,46393)	54882 (43513,69623)	319.77 (254.26,403.03)	370.5 (296.87,469.79)	0.50 (0.48, 0.53)
Seychelles	335 (268,420)	909 (709,1170)	588.21 (467.93,739.23)	736.68 (580.23,933.26)	0.71 (0.70, 0.73)
Sierra Leone	8878 (7142,11106)	18727 (14981,23764)	405 (323.75,506.27)	429.51 (341.9,536.05)	0.21 (0.17, 0.25)
Singapore	16253 (12961,20576)	69640 (54316,89478)	641.47 (509.68,803.58)	805.67 (634.66,1024.8)	0.77 (0.72, 0.82)
Slovakia	18313 (14707,23123)	31438 (24653,39568)	312.79 (251.45,394.63)	357.42 (284.6,446.39)	0.39 (0.38, 0.41)
Slovenia	7491 (5932,9438)	14157 (11206,17782)	311.16 (247.69,390.56)	370.53 (297.59,465.83)	0.58 (0.57, 0.59)
Solomon Islands	1025 (807,1288)	2872 (2231,3652)	663.23 (525.52,821.93)	708.91 (563.44,882.7)	0.15 (0.13, 0.17)
Somalia	11739 (9147,15001)	29485 (23194,37847)	401.18 (318.2,500.3)	379.88 (302.81,477.27)	-0.15 (-0.17, -0.13)
South Africa	115000 (91679,144253)	282940 (223685,357508)	516.9 (413.66,641.63)	568.28 (454.94,709.03)	0.37 (0.32, 0.41)
South Sudan	12881 (10118,16189)	21057 (16360,26729)	469.36 (372.06,590.71)	475.62 (377.21,594.34)	0.08 (0.06, 0.10)
Spain	288908 (228531,368759)	540193 (423897,683959)	565.18 (447.93,710.92)	640.05 (506.16,805.26)	0.37 (0.32, 0.43)
Sri Lanka	68739 (54631,87886)	172336 (133802,218518)	566.09 (454.39,715.75)	643.22 (507.14,805.82)	0.41 (0.38, 0.45)
Sudan	39410 (31156,49230)	110250 (87392,139426)	392.88 (313.26,490.64)	488.2 (388.41,616.83)	0.76 (0.74, 0.78)

Suriname	550 (438,696)	1577 (1238,1978)	195.97 (157.78,248.04)	247.22 (197.56,306.92)	0.79 (0.77, 0.80)
Sweden	75180 (58566,96011)	111499 (85843,145464)	564.9 (441.2,718.91)	613.14 (473.58,793.05)	-0.03 (-0.15, 0.08)
Switzerland	53054 (42160,66997)	97603 (77226,123683)	552.65 (430.4,693.38)	622.22 (493.67,786.47)	0.40 (0.39, 0.42)
Syrian Arab Republic	25875 (20514,32696)	70530 (54426,90158)	450.34 (360.34,566.57)	509.33 (406.37,640.59)	0.41 (0.34, 0.47)
Taiwan (Province of China)	146508 (117486,179758)	409169 (332940,502585)	842.33 (681.72,1026.4)	1054.11 (865.35,1295.57)	0.36 (-0.36, 1.09)
Tajikistan	10066 (8050,12697)	26305 (20676,33697)	346.82 (276.22,430.77)	395.59 (314.09,495.9)	0.48 (0.44, 0.52)
Thailand	236370 (189782,293237)	729075 (571691,924526)	577.32 (465,709.56)	699.58 (555.19,873.59)	0.88 (0.79, 0.97)
Timor-Leste	1953 (1519,2465)	5374 (4275,6829)	531.55 (422.01,672.62)	593.94 (473.08,749)	0.38 (0.35, 0.40)
Togo	5624 (4429,7098)	18927 (14790,23680)	394.58 (313.37,495.63)	414.51 (327.76,517.1)	0.14 (0.12, 0.16)
Tokelau	9 (7,11)	12 (9,15)	648.81 (521.97,804.37)	795.09 (634.2,999.42)	0.63 (0.61, 0.65)
Tonga	423 (333,527)	683 (543,868)	722.21 (574.41,898.81)	825.27 (654.22,1047.88)	0.32 (0.28, 0.36)
Trinidad and Tobago	1899 (1514,2348)	4913 (3920,6194)	207.34 (166.34,259.58)	267.01 (213.87,336.01)	0.86 (0.84, 0.89)
Tunisia	23313 (18241,29350)	68199 (53802,85676)	437.11 (342.64,547.73)	501.64 (397.61,626.88)	0.47 (0.46, 0.49)
Turkey	164114 (129029,207007)	496371 (389149,627794)	435.49 (343.42,546.34)	518.83 (411.41,651.44)	0.58 (0.54, 0.61)
Turkmenistan	7843 (6204,9900)	20318 (15900,26052)	381.16 (304.37,479.72)	465.57 (371.16,584.51)	0.69 (0.65, 0.74)
Tuvalu	42 (33,54)	81 (64,102)	596.59 (471.82,748.8)	748.71 (595.57,942.64)	0.67 (0.64, 0.70)
Uganda	28888 (22516,36477)	74125 (58605,92943)	409.2 (322.7,505.52)	425.84 (338.95,530.51)	0.19 (0.14, 0.24)
Ukraine	262892 (208500,335398)	300892 (236467,380231)	383.23 (307.68,479.76)	417.87 (332.53,523.45)	0.30 (0.28, 0.31)
United Arab Emirates	5162 (3912,6735)	69163 (50295,92065)	566.53 (448.42,707.41)	771.03 (608.78,975.53)	1.07 (1.03, 1.10)
United Kingdom	463434 (364925,588664)	748964 (590475,947107)	570.61 (450.05,719.56)	660.9 (525,832.54)	0.80 (0.62, 0.98)
United Republic of Tanzania	50253 (39718,63438)	134767 (107163,171366)	429.17 (341.48,537.01)	464.59 (367.81,587.92)	0.28 (0.24, 0.32)
United States of America	2862314 (2303631,3584518)	8569247 (6984855,10498488)	951.37 (754.69,1194.66)	1677.1 (1369.5,2044.08)	2.76 (2.44, 3.09)

United States Virgin Islands	201 (156,256)	416 (328,523)	211.6 (169.54,267.19)	279.57 (222.88,350.5)	0.92 (0.87, 0.97)
Uruguay	27331 (21535,34524)	43623 (34522,55427)	739.12 (582.18,927.42)	887.77 (705.03,1123.72)	0.57 (0.54, 0.60)
Uzbekistan	46649 (37465,58587)	130959 (102329,166909)	381.45 (303.76,480.07)	451.98 (358.33,566.89)	0.56 (0.54, 0.58)
Vanuatu	488 (386,612)	1443 (1133,1821)	670.95 (530.49,843.79)	726.16 (582.32,899.99)	0.25 (0.24, 0.26)
Venezuela (Bolivarian Republic of)	16543 (13231,20881)	52660 (41905,66755)	137.24 (109.52,171.35)	173.39 (139.27,217.51)	0.77 (0.74, 0.80)
Viet Nam	205216 (163606,258315)	616838 (480289,786054)	481.09 (383.37,598)	576.93 (457.83,719.4)	0.71 (0.65, 0.77)
Yemen	20146 (15953,25938)	68769 (55048,87616)	359.79 (289.33,458.45)	412.07 (330.69,513.43)	0.51 (0.48, 0.54)
Zambia	13845 (10926,17435)	38840 (30582,49362)	442.6 (351.07,558.12)	461.98 (364.67,584.59)	0.13 (0.08, 0.18)
Zimbabwe	20737 (16203,26521)	35868 (28225,45238)	460.79 (362.78,579.13)	451.27 (356.36,567.74)	-0.12 (-0.16, -0.08)

Table S3. Gout Incidence in 204 countries and territories between 1990 and 2021.

**Location**	**Incidence case No. (95% UI)**		**ASIR per 100,000 No. (95% UI)**		**1990-2021EAPC in ASIR No. (95% CI)**
	**1990**	**2021**	**1990**	**2021**	
Afghanistan	5221 (4098,6655)	11078 (8636,14020)	75.46 (59.96,95.17)	82.99 (65.83,104.34)	0.38 (0.30, 0.46)
Albania	1414 (1122,1755)	2700 (2130,3416)	60.55 (48.49,75.77)	68.22 (54.05,85.22)	0.42 (0.40, 0.44)
Algeria	11346 (9050,14057)	38672 (30863,48273)	82.73 (66.17,103.5)	98.13 (79.05,122.15)	0.60 (0.58, 0.62)
American Samoa	43 (34,54)	83 (64,104)	151.47 (119.92,191.04)	164.86 (131.27,204.78)	0.21 (0.18, 0.23)
Andorra	51 (40,64)	121 (95,152)	84.31 (66.81,105.77)	86.25 (68.04,107.7)	0.03 (0.01, 0.06)
Angola	3782 (2968,4694)	12362 (9710,15447)	79.19 (63.3,99.22)	82.2 (64.67,103.64)	0.15 (0.12, 0.18)
Antigua and Barbuda	20 (16,25)	52 (42,66)	37.2 (30.25,46.42)	48.81 (39.69,60.78)	0.88 (0.86, 0.91)
Argentina	34667 (27072,43295)	66833 (52138,83735)	107.8 (84.11,133.93)	126.05 (98.83,157.37)	0.44 (0.40, 0.47)
Armenia	2099 (1673,2647)	3480 (2722,4434)	71.73 (57.51,90.09)	85.52 (67.77,108.6)	0.66 (0.62, 0.71)
Australia	26154	66556	137.34	174.38	0.82 (0.73,

	(20856,32440)	(51822,83497)	(109.68,170.38)	(136.4,215.46)	0.90)
Austria	7718 (6165,9608)	12083 (9612,15179)	74.91 (59.45,93.25)	83.33 (66.11,104.11)	0.31 (0.28, 0.33)
Azerbaijan	3906 (3104,4855)	9731 (7567,12250)	71.33 (56.66,89.57)	86.11 (68.23,108.48)	0.70 (0.66, 0.75)
Bahamas	75 (61,93)	210 (168,263)	39.73 (31.91,49.45)	49.2 (39.78,60.95)	0.71 (0.68, 0.74)
Bahrain	283 (221,356)	1624 (1256,2069)	95.77 (75.75,119.39)	113.52 (89.79,141.87)	0.60 (0.57, 0.63)
Bangladesh	39950 (32376,49579)	113151 (90066,142364)	73.33 (58.73,92.2)	77.19 (61.94,97.39)	0.18 (0.17, 0.19)
Barbados	106 (87,132)	219 (176,275)	39.36 (32.13,49.02)	49.69 (40.15,61.61)	0.76 (0.73, 0.78)
Belarus	8636 (6786,10788)	11288 (8899,14437)	69.01 (54.42,86.37)	77.46 (61.67,97.4)	0.39 (0.37, 0.42)
Belgium	10294 (8203,13097)	14984 (11743,18767)	76.34 (60,97.31)	82.38 (64.6,101.45)	0.21 (0.19, 0.24)
Belize	42 (34,53)	183 (146,228)	39.3 (31.7,49.32)	52.15 (42.27,64.96)	0.91 (0.84, 0.97)
Benin	1755 (1414,2159)	5632 (4513,7090)	77.87 (61.16,97.66)	87.18 (69.31,111.69)	0.37 (0.35, 0.38)
Bermuda	27 (22,34)	57 (46,72)	41.49 (33.79,52.38)	52.36 (42,65.81)	0.77 (0.75, 0.79)
Bhutan	221 (176,273)	556 (444,695)	72.86 (57.37,91.51)	83.25 (65.66,105.07)	0.47 (0.44, 0.49)
Bolivia (Plurinational State of)	1593 (1281,1972)	5572 (4476,6849)	41.42 (33.61,51.73)	54.64 (44.08,67.37)	0.91 (0.88, 0.94)
Bosnia and Herzegovina	2551 (2030,3230)	3735 (2966,4768)	58.06 (47.14,73.1)	68.4 (53.91,85.44)	0.54 (0.52, 0.55)
Botswana	540 (425,670)	1779 (1402,2224)	83.29 (66.11,104.1)	98.09 (77.75,122.67)	0.52 (0.48, 0.57)
Brazil	41287 (33187,51297)	126134 (101588,157616)	39.51 (31.95,49.6)	49.95 (40.4,62.01)	0.79 (0.76, 0.82)
Brunei Darussalam	156 (125,194)	499 (388,631)	103.23 (81.85,127.91)	112.32 (88.74,140.99)	0.23 (0.20, 0.27)
Bulgaria	7493 (5890,9438)	8303 (6511,10590)	64.25 (51.28,79.65)	69.11 (54.93,86.39)	0.25 (0.21, 0.28)
Burkina Faso	3537 (2837,4432)	9039 (7214,11191)	74.09 (60,93.17)	81.04 (63.87,101.4)	0.31 (0.30, 0.32)
Burundi	2051 (1628,2540)	5168 (4070,6501)	77.11 (61.09,95.84)	83.82 (66.93,106.22)	0.32 (0.30, 0.33)
Cabo Verde	164 (132,205)	439 (346,551)	73.47 (58.6,93.61)	87.8 (69.51,110.42)	0.64 (0.61, 0.68)

Cambodia	5017 (3994,6198)	15301 (12192,19060)	92.32 (73.08,114.57)	106.72 (85.29,131.94)	0.51 (0.47, 0.54)
Cameroon	4437 (3508,5499)	15113 (11957,18715)	85.69 (68,106.69)	94.12 (74.82,118.95)	0.32 (0.30, 0.34)
Canada	48762 (38389,60956)	101449 (79081,127224)	154.5 (121.38,193.6)	178.63 (141.01,222.44)	0.48 (0.42, 0.55)
Central African Republic	1037 (812,1286)	2212 (1732,2770)	76.55 (60.46,95.95)	77.84 (61.56,98.43)	0.09 (0.08, 0.10)
Chad	2274 (1818,2831)	5836 (4589,7251)	73.99 (59.02,92.63)	82.25 (65.08,103.5)	0.39 (0.38, 0.40)
Chile	12390 (9861,15445)	31965 (25046,39473)	112.67 (89.5,141.19)	132.88 (104.18,164.9)	0.47 (0.42, 0.51)
China	1182498 (940490,1461669)	3079836 (2425498,3891398)	122.52 (97.99,152.96)	151.61 (121.16,189.2)	0.98 (0.86, 1.09)
Colombia	5923 (4704,7427)	18571 (14993,23210)	26.17 (20.95,32.88)	33.77 (27.38,42.22)	0.79 (0.77, 0.82)
Comoros	184 (146,231)	474 (373,589)	81.72 (65.5,102.75)	85.67 (68.28,106.64)	0.17 (0.16, 0.19)
Congo	972 (759,1216)	3145 (2463,3976)	80.02 (63.79,100.61)	92.68 (73.96,117.33)	0.51 (0.49, 0.54)
Cook Islands	20 (16,25)	37 (29,47)	142.35 (112.56,177.76)	155.38 (122.76,194.38)	0.23 (0.20, 0.26)
Costa Rica	617 (495,776)	1947 (1563,2407)	28.61 (23.04,36.06)	35.75 (28.81,44.46)	0.69 (0.67, 0.71)
Croatia	3662 (2839,4632)	5235 (4111,6733)	60.62 (47.75,76.91)	71.02 (56.74,89.21)	0.22 (0.19, 0.26)
Cuba	4177 (3397,5212)	8371 (6643,10580)	39.63 (31.97,49.7)	49.39 (39.62,61.55)	0.54 (0.52, 0.56)
Cyprus	622 (486,769)	1540 (1213,1920)	75.5 (59.24,93.4)	80.1 (63.66,99.04)	0.75 (0.72, 0.78)
Czechia	7892 (6304,9943)	12877 (10206,16396)	60.78 (48.35,76.68)	71.17 (56.81,88.93)	0.14 (0.10, 0.17)
Côte d'Ivoire	4401 (3470,5430)	13136 (10357,16553)	84.32 (66.79,104.6)	89.93 (70.88,113.85)	0.63 (0.45, 0.81)
Democratic People's Republic of Korea	23302 (18195,29139)	47491 (37242,59113)	127.31 (101.64,159.41)	143.03 (112.62,176.84)	0.41 (0.37, 0.45)
Democratic Republic of the Congo	14724 (11616,18328)	38006 (30030,47042)	81.6 (64.91,102.02)	82.13 (65.17,101.86)	0.02 (-0.05, 0.08)
Denmark	5306 (4172,6621)	7630 (5948,9589)	75.74 (59.7,94.42)	82.19 (65.1,101.84)	0.27 (0.24, 0.29)
Djibouti	149 (117,186)	749 (583,950)	80.74 (63.7,102.01)	90.38 (71.33,114.74)	0.41 (0.39, 0.43)
Dominica	23 (18,28)	41 (32,51)	38.68 (31.13,48.69)	51.06 (40.73,63.45)	0.90 (0.86, 0.93)

Dominican Republic	1641 (1327,2062)	5075 (4127,6291)	36.69 (29.6,46.32)	47.86 (38.92,59.25)	0.92 (0.89, 0.95)
Ecuador	2922 (2369,3632)	10118 (8120,12552)	46.06 (36.94,57.83)	59.07 (47.31,73.36)	1.02 (0.94, 1.10)
Egypt	27478 (21333,34488)	78734 (62071,100019)	86.03 (68.25,107.97)	106.37 (84.63,132.96)	0.64 (0.63, 0.65)
El Salvador	912 (738,1139)	2124 (1730,2654)	26.19 (21.1,32.73)	33.85 (27.44,42.57)	0.80 (0.76, 0.84)
Equatorial Guinea	163 (128,202)	667 (530,827)	74.97 (59.48,93.24)	93.77 (74.55,116.99)	0.91 (0.84, 0.99)
Eritrea	1093 (854,1373)	2864 (2247,3559)	73.56 (57.81,92.24)	78.19 (62.74,97.78)	0.19 (0.17, 0.22)
Estonia	1384 (1111,1718)	1842 (1485,2326)	70.66 (56.81,87.35)	84.01 (66.03,105.54)	0.62 (0.60, 0.64)
Eswatini	325 (256,407)	694 (546,873)	92.26 (72.63,115.22)	98.71 (78,124.75)	0.18 (0.16, 0.20)
Ethiopia	19452 (15461,24349)	47667 (38151,59169)	84.89 (67.98,106.81)	90.1 (71.73,113.02)	0.19 (0.18, 0.20)
Fiji	613 (481,764)	1279 (1001,1580)	132.12 (104.82,165.2)	149.28 (117.35,183.57)	0.34 (0.32, 0.36)
Finland	4895 (3844,6056)	7679 (5871,9676)	74.68 (58.17,92.25)	82.64 (64.85,102.51)	0.28 (0.25, 0.30)
France	54489 (43259,69535)	85633 (68201,108088)	74.61 (58.66,94.91)	81.21 (64.35,102.07)	0.24 (0.21, 0.26)
Gabon	499 (400,623)	1183 (928,1479)	82.27 (65.88,102.97)	96.43 (76.1,120)	0.54 (0.52, 0.55)
Gambia	352 (278,439)	1042 (839,1293)	81.37 (63.58,102.5)	86.32 (69.5,108.21)	0.18 (0.17, 0.20)
Georgia	4565 (3618,5687)	4331 (3462,5480)	74.09 (58.65,93.31)	81.91 (65.16,102.79)	0.36 (0.33, 0.40)
Germany	85718 (67085,108088)	123253 (97912,155303)	76.67 (60.2,95.86)	84.51 (67.15,105.02)	0.31 (0.29, 0.33)
Ghana	5746 (4531,7102)	18102 (14130,22839)	77.07 (61.13,96.89)	87.45 (68.84,110.33)	0.39 (0.37, 0.41)
Greece	11474 (9017,14561)	15421 (12127,19422)	83.48 (66.18,106.34)	87.4 (69.81,108.27)	0.37 (0.04, 0.71)
Greenland	70 (54,88)	116 (91,145)	147.69 (116.62,181.81)	165.82 (132.13,203.56)	0.44 (0.38, 0.51)
Grenada	25 (20,31)	56 (45,70)	36.38 (29.39,45.03)	48.73 (39.13,60.26)	0.88 (0.85, 0.91)
Guam	135 (107,166)	299 (234,378)	138.44 (110.65,171.86)	150.43 (119.89,187.51)	0.28 (0.26, 0.29)
Guatemala	1118 (884,1413)	4127 (3304,5201)	24.91 (20.15,31.3)	32.44 (26.26,40.66)	0.84 (0.82, 0.86)

Guinea	2764 (2191,3458)	5532 (4380,6881)	78.15 (62.64,98.08)	83.4 (66.3,104.6)	0.22 (0.20, 0.24)
Guinea-Bissau	351 (279,439)	756 (595,944)	76.12 (60.55,95.66)	78.9 (62.17,98.63)	0.12 (0.10, 0.13)
Guyana	179 (144,223)	333 (263,418)	37.53 (30.38,46.18)	48.01 (38.82,60.09)	0.76 (0.74, 0.78)
Haiti	1326 (1049,1660)	3795 (2989,4725)	34.57 (27.97,43.12)	41.58 (33.85,51.48)	0.62 (0.60, 0.64)
Honduras	657 (523,819)	2533 (2048,3140)	25.25 (20.29,31.31)	32.66 (26.52,40.73)	0.82 (0.78, 0.86)
Hungary	8568 (6835,10928)	11553 (9183,14822)	62.18 (49.5,78.58)	70.13 (56.25,87.5)	0.38 (0.37, 0.39)
Iceland	215 (168,270)	420 (331,522)	80.04 (63.03,100.81)	85.71 (68.17,105.35)	0.20 (0.17, 0.23)
India	414246 (328672,512665)	1065035 (843417,1330198)	77.63 (61.6,97.09)	83.23 (66.05,104.36)	0.21 (0.19, 0.23)
Indonesia	122798 (96606,151380)	350045 (275421,436794)	103.35 (82.34,128.94)	126.75 (101.03,159.42)	0.66 (0.63, 0.68)
Iran (Islamic Republic of)	25817 (20321,32005)	83979 (66223,105369)	85.96 (68.12,107.38)	97.11 (76.89,122.26)	0.37 (0.30, 0.44)
Iraq	8214 (6554,10205)	28747 (22506,36118)	88.95 (70.56,112.21)	98.66 (77.65,124.07)	0.33 (0.30, 0.36)
Ireland	3025 (2376,3863)	5899 (4670,7444)	79.76 (62.96,100.85)	85.08 (67.8,107.23)	0.18 (0.16, 0.20)
Israel	3668 (2918,4598)	9246 (7241,11702)	78.72 (61.88,98.22)	84.8 (66.86,106.88)	0.21 (0.18, 0.23)
Italy	62070 (49047,78329)	91303 (71121,114815)	78.41 (61.53,98.23)	82.91 (65.36,103.28)	0.22 (0.11, 0.33)
Jamaica	709 (583,868)	1574 (1279,1958)	38.96 (31.6,48.21)	50.36 (40.97,62.71)	0.84 (0.79, 0.89)
Japan	176965 (138855,225139)	292600 (228340,374291)	107.32 (84.52,134.92)	116.69 (91.91,147.52)	0.22 (0.19, 0.24)
Jordan	1526 (1203,1886)	10159 (7984,12674)	90.04 (70.88,112.48)	109.52 (87.45,137.75)	0.67 (0.64, 0.70)
Kazakhstan	9916 (7898,12323)	16276 (12774,20537)	72.95 (58.29,91.41)	85.16 (67.61,107.46)	0.54 (0.51, 0.58)
Kenya	8537 (6826,10549)	26355 (20890,32721)	88.31 (70.53,111.03)	93.46 (74.35,117.24)	0.22 (0.17, 0.27)
Kiribati	54 (42,67)	113 (89,140)	121.43 (95.98,151.83)	128.68 (101.45,159.98)	0.13 (0.12, 0.15)
Kuwait	1043 (813,1318)	5028 (3879,6480)	105.01 (82.81,132.87)	115.84 (92.27,144.4)	0.43 (0.37, 0.49)
Kyrgyzstan	2228 (1768,2803)	4360 (3389,5421)	70.52 (56.32,88.87)	79.39 (62.67,98.2)	0.38 (0.37, 0.40)

Lao People's Democratic Republic	2369 (1892,2938)	6670 (5291,8223)	98.73 (78.27,123.13)	118.4 (94.47,147.09)	0.64 (0.61, 0.66)
Latvia	2400 (1924,3040)	2621 (2088,3339)	70.54 (56.35,88.74)	80.79 (64.19,101.32)	0.46 (0.44, 0.48)
Lebanon	1920 (1501,2432)	5767 (4624,7159)	82.55 (65.7,104.37)	95.86 (76.83,119.19)	0.49 (0.46, 0.53)
Lesotho	703 (549,877)	1119 (880,1391)	76.4 (60.31,95.63)	89.07 (70.48,112.24)	0.51 (0.49, 0.53)
Liberia	1097 (872,1371)	2626 (2089,3297)	85.67 (68.26,107.49)	92.3 (73.17,115.24)	0.38 (0.31, 0.45)
Libya	2001 (1590,2497)	6436 (5047,8162)	90.52 (72.24,113.9)	100.78 (79.84,127.03)	0.37 (0.34, 0.40)
Lithuania	3120 (2496,3913)	3742 (3000,4715)	71.44 (57.39,89.3)	78.86 (62.28,98.79)	0.33 (0.32, 0.35)
Luxembourg	391 (306,487)	794 (626,995)	77.58 (61.18,96.73)	84.01 (66.98,104.4)	0.23 (0.21, 0.26)
Madagascar	4676 (3740,5769)	11966 (9399,14939)	81.21 (64.47,101.05)	83.41 (66.42,103.41)	0.12 (0.09, 0.15)
Malawi	3560 (2785,4417)	7714 (6169,9670)	78.99 (63.08,99.08)	83.02 (66.39,104.98)	0.18 (0.16, 0.19)
Malaysia	12445 (9980,15521)	41250 (32780,50841)	109.2 (87.41,137.35)	132.74 (105.32,163.95)	0.65 (0.64, 0.66)
Maldives	121 (95,151)	692 (547,890)	112.38 (89.36,140.71)	138.45 (108.42,174.67)	0.70 (0.62, 0.77)
Mali	3426 (2709,4218)	8963 (7025,11132)	76.4 (61.14,94.78)	84.42 (67.13,105.01)	0.35 (0.32, 0.37)
Malta	320 (253,403)	622 (480,788)	75.26 (59.36,94.4)	83.54 (65.92,105.18)	0.28 (0.25, 0.32)
Marshall Islands	25 (20,31)	59 (46,74)	120.7 (94.51,149.74)	137.05 (109.13,171.32)	0.39 (0.38, 0.41)
Mauritania	911 (721,1127)	2323 (1846,2867)	81.82 (64.94,102.1)	93.86 (74.37,117.48)	0.42 (0.41, 0.44)
Mauritius	934 (743,1164)	2324 (1817,2927)	109.24 (86.45,135.97)	132.04 (104.87,165.14)	0.62 (0.61, 0.63)
Mexico	17892 (14333,22285)	52823 (42139,66135)	33.32 (26.85,41.53)	39.61 (31.87,49.4)	0.78 (0.67, 0.89)
Micronesia (Federated States of)	73 (57,92)	123 (96,155)	130.54 (103.3,164.66)	144.39 (113.36,178.75)	0.27 (0.26, 0.29)
Monaco	43 (34,54)	61 (48,77)	80.07 (63.24,101.74)	85.32 (67.2,106.57)	0.18 (0.15, 0.21)
Mongolia	872 (693,1082)	2198 (1718,2779)	73.45 (58.35,92.46)	79.42 (63.48,100.17)	0.31 (0.28, 0.34)
Montenegro	417 (330,524)	653 (512,835)	65.39 (51.83,82.31)	72.63 (57.72,91.3)	0.40 (0.37, 0.43)

Morocco	12476 (10009,15506)	34166 (27139,42641)	79.1 (62.77,99.97)	93.67 (75.12,117.51)	0.56 (0.53, 0.58)
Mozambique	5325 (4165,6631)	11284 (8884,13970)	77.58 (61.22,97.91)	80.78 (63.85,102.04)	0.17 (0.15, 0.19)
Myanmar	26194 (20415,32449)	59330 (46390,73479)	98.31 (77.8,122.45)	111.73 (87.64,137.93)	0.45 (0.42, 0.48)
Namibia	633 (501,790)	1505 (1197,1863)	85.59 (67.32,108.62)	91.7 (72.62,114.75)	0.23 (0.21, 0.26)
Nauru	8 (6,10)	10 (8,13)	142.6 (112.57,176.81)	140.45 (111.13,176.1)	-0.09 (-0.18, -0.00)
Nepal	7720 (6060,9584)	19320 (15128,24145)	71.23 (56.92,89.72)	77.12 (60.76,96.85)	0.28 (0.27, 0.29)
Netherlands	14808 (11690,18537)	24029 (18639,30508)	80.16 (62.6,100.36)	86.33 (68.51,108.77)	0.02 (-0.06, 0.10)
New Zealand	7142 (5663,8964)	14963 (11646,18916)	189.98 (151.13,239.42)	205.24 (161.12,256.24)	0.02 (-0.15, 0.19)
Nicaragua	533 (423,674)	1991 (1614,2488)	25.85 (20.94,32.69)	34.44 (28.05,43.1)	0.90 (0.87, 0.93)
Niger	2679 (2131,3292)	7988 (6364,9998)	78.31 (61.72,97.38)	79.73 (64.42,100.75)	0.06 (0.05, 0.07)
Nigeria	43325 (34116,53618)	93266 (74023,115624)	86.56 (68.54,108.45)	81.47 (64.75,102.26)	-0.26 (-0.35, -0.18)
Niue	3 (2,3)	3 (2,4)	130.03 (101.34,162.74)	150.73 (118.68,190.17)	0.46 (0.43, 0.49)
North Macedonia	1221 (955,1525)	2299 (1809,2900)	62.87 (50.02,78.59)	72.32 (58.09,90.41)	0.48 (0.46, 0.49)
Northern Mariana Islands	48 (37,61)	91 (71,118)	162.35 (125.46,200.81)	161.78 (127.81,204.61)	-0.07 (-0.09, -0.04)
Norway	4421 (3494,5563)	6921 (5454,8688)	77.41 (60.96,97.26)	83.65 (66.23,104.4)	0.08 (-0.13, 0.28)
Oman	856 (674,1079)	3856 (2981,4964)	84.06 (66.4,105.92)	109.78 (86.21,136.95)	0.83 (0.80, 0.87)
Pakistan	44659 (35699,55462)	121625 (95189,151398)	71.78 (57.03,89.43)	83.7 (66.03,105.14)	0.64 (0.56, 0.72)
Palau	16 (12,20)	38 (30,49)	138.3 (109.92,173.27)	161.83 (128.7,201.96)	0.40 (0.34, 0.47)
Palestine	780 (624,970)	2980 (2366,3683)	79.46 (63.17,100.39)	93.63 (74.28,115.96)	0.46 (0.44, 0.49)
Panama	454 (364,568)	1543 (1251,1909)	25.66 (20.75,31.98)	34.88 (28.3,43.02)	0.96 (0.95, 0.98)
Papua New Guinea	2636 (2056,3318)	8227 (6489,10261)	117.3 (93.09,145.45)	125.99 (101.12,158.08)	0.22 (0.21, 0.23)
Paraguay	985 (796,1206)	3076 (2497,3815)	38.01 (30.97,47.01)	47.79 (38.77,59.81)	0.74 (0.72, 0.75)

Peru	5921 (4810,7388)	19420 (15535,24108)	42.01 (34.16,52.57)	54.5 (43.79,68.14)	0.89 (0.87, 0.91)
Philippines	35857 (28442,44463)	113259 (89297,140810)	98.86 (78.98,123.59)	120.55 (95.58,151.27)	0.63 (0.58, 0.69)
Poland	27491 (21899,34446)	45698 (36567,57774)	64.14 (51.21,80.31)	73.14 (58.48,91.5)	0.40 (0.39, 0.41)
Portugal	9641 (7510,12063)	15243 (11941,19203)	75.54 (58.73,94.3)	81.93 (64.45,102.34)	0.29 (0.23, 0.35)
Puerto Rico	1520 (1239,1896)	2986 (2423,3758)	42.32 (34.38,52.7)	53.98 (43.66,67.95)	0.79 (0.76, 0.81)
Qatar	297 (227,391)	3208 (2429,4167)	110.7 (88.01,137.25)	130.41 (102.73,162.7)	0.64 (0.59, 0.69)
Republic of Korea	34248 (26593,42613)	90755 (70548,115114)	93.99 (74.01,118.73)	107.16 (84.03,133.97)	0.38 (0.35, 0.41)
Republic of Moldova	3093 (2457,3870)	4375 (3437,5593)	69.34 (55.24,85.56)	79.09 (62.23,100.18)	0.49 (0.45, 0.52)
Romania	16640 (13124,21020)	22333 (17854,28287)	61.32 (48.65,76.87)	70.26 (56.48,87.73)	0.46 (0.45, 0.48)
Russian Federation	131013 (103569,163912)	188569 (148775,239234)	73.97 (58.49,92.81)	85.91 (68.37,107.88)	0.50 (0.49, 0.51)
Rwanda	2540 (2014,3150)	6205 (4889,7714)	76.83 (61.12,96.18)	80.07 (63.05,100.14)	0.16 (0.11, 0.21)
Saint Kitts and Nevis	14 (11,17)	36 (29,46)	39.24 (31.78,48.63)	50.39 (41.36,63.37)	0.79 (0.75, 0.82)
Saint Lucia	35 (29,44)	115 (92,142)	37.58 (30.46,47.36)	49.59 (40.13,60.91)	0.88 (0.83, 0.93)
Saint Vincent and the Grenadines	28 (23,34)	68 (54,85)	36.63 (29.71,45.55)	49.66 (40.07,61.45)	1.00 (0.97, 1.04)
Samoa	134 (104,166)	238 (187,300)	138.75 (109.19,172.98)	150.59 (118.94,189)	0.20 (0.18, 0.22)
San Marino	25 (20,31)	47 (37,60)	80.02 (62.87,99.6)	82.72 (66.02,102.43)	0.06 (0.04, 0.09)
Sao Tome and Principe	54 (43,67)	126 (100,156)	78.79 (63,99.31)	91.54 (73.09,114.18)	0.51 (0.49, 0.53)
Saudi Arabia	7363 (5790,9248)	36532 (28254,46722)	92.97 (73.69,117.6)	119.29 (95.06,151.76)	0.79 (0.76, 0.81)
Senegal	2940 (2341,3664)	7639 (6027,9608)	80.17 (63.55,100.24)	84.23 (66.17,105.86)	0.17 (0.16, 0.18)
Serbia	7076 (5523,8937)	10234 (8174,12967)	62.58 (49.83,78.32)	71.54 (57.3,89.2)	0.45 (0.43, 0.48)
Seychelles	64 (51,79)	167 (131,211)	110.44 (87.45,137.76)	135.08 (107.06,168.82)	0.65 (0.63, 0.66)
Sierra Leone	1783 (1433,2227)	3850 (3077,4826)	78.17 (62.08,98.39)	83 (66.07,105.24)	0.21 (0.17, 0.25)

Singapore	2636 (2074,3303)	9797 (7634,12370)	97.1 (76.55,121.95)	115.05 (90.34,144.09)	0.54 (0.49, 0.59)
Slovakia	3570 (2865,4460)	5930 (4734,7503)	61.24 (49.33,76.49)	69.31 (55.51,86.35)	0.37 (0.36, 0.38)
Slovenia	1458 (1165,1825)	2613 (2085,3351)	61.01 (48.7,76.01)	71.49 (56.21,91.02)	0.53 (0.51, 0.54)
Solomon Islands	206 (162,256)	566 (441,707)	125.11 (99.63,156.61)	131.94 (104.36,164.38)	0.13 (0.11, 0.15)
Somalia	2491 (1958,3119)	6375 (4991,7956)	77.76 (61.55,97.15)	74.74 (59.45,93.24)	-0.11 (-0.13, -0.09)
South Africa	23150 (18303,28801)	55943 (43993,69656)	99.84 (78.95,124.99)	109.29 (86.8,136.8)	0.35 (0.31, 0.39)
South Sudan	2580 (2046,3167)	4263 (3304,5424)	89.64 (71.01,111.38)	90.25 (70.75,114.9)	0.05 (0.04, 0.07)
Spain	38118 (30032,47984)	64437 (50967,80907)	78.04 (61.71,98.49)	83.79 (66.54,104.18)	0.18 (0.14, 0.23)
Sri Lanka	13566 (10792,17039)	31668 (25141,40033)	107.21 (84.8,134.36)	119.54 (95.58,149.81)	0.35 (0.32, 0.38)
Sudan	8038 (6445,9985)	22364 (17963,27805)	76.68 (61.14,95.97)	92.96 (74.18,116.83)	0.68 (0.65, 0.70)
Suriname	111 (90,137)	305 (247,382)	38.62 (31.42,47.79)	48.2 (39.07,60.16)	0.74 (0.72, 0.76)
Sweden	11703 (9191,14751)	15980 (12311,20363)	93.01 (72.39,118.45)	95.25 (74.36,119.82)	-0.05 (-0.10, 0.00)
Switzerland	7072 (5640,8847)	11808 (9349,14849)	76.82 (60.81,95.64)	82.02 (64.98,102.11)	0.19 (0.16, 0.22)
Syrian Arab Republic	5181 (4103,6421)	13406 (10552,16895)	85.41 (68.26,106.03)	95.56 (76.3,119.8)	0.37 (0.31, 0.43)
Taiwan (Province of China)	27227 (21887,33014)	66502 (52975,83803)	151.39 (121.8,184.58)	177.28 (142.32,218.37)	0.33 (-0.16, 0.82)
Tajikistan	2049 (1630,2545)	5352 (4230,6732)	67.96 (53.87,85.63)	76.57 (60.9,95.36)	0.44 (0.40, 0.47)
Thailand	46545 (37793,57190)	129901 (101340,165078)	108.39 (87.11,132.71)	127.91 (100.57,159.83)	0.75 (0.68, 0.82)
Timor-Leste	407 (320,518)	1052 (830,1312)	102.14 (81.61,128.93)	112.98 (89.37,141.58)	0.34 (0.32, 0.36)
Togo	1165 (923,1438)	3880 (3045,4848)	76.12 (60.05,94.99)	79.89 (62.53,100.55)	0.13 (0.11, 0.15)
Tokelau	2 (1,2)	2 (2,3)	121.48 (96.54,150.91)	144.2 (114.75,179.82)	0.54 (0.53, 0.56)
Tonga	80 (63,99)	124 (98,155)	132.3 (104.83,165.35)	147.59 (116.51,185.24)	0.27 (0.24, 0.30)
Trinidad and Tobago	380 (310,471)	930 (747,1169)	40.46 (32.74,50.58)	51.51 (41.38,63.96)	0.82 (0.79, 0.84)

Tunisia	4592 (3620,5787)	12914 (10093,16433)	83.21 (65.96,104.87)	94.71 (74.46,119.87)	0.42 (0.41, 0.44)
Turkey	32525 (25773,40043)	93211 (73077,117785)	82.95 (65.18,102.36)	97.2 (76.19,122.52)	0.52 (0.49, 0.55)
Turkmenistan	1582 (1262,1962)	3942 (3044,4949)	73.34 (58.03,92.33)	87.55 (68.58,109.75)	0.62 (0.57, 0.66)
Tuvalu	8 (6,10)	15 (12,19)	112.95 (89.99,141.28)	138.05 (109.8,172.59)	0.61 (0.58, 0.63)
Uganda	5931 (4681,7370)	15388 (12336,19154)	79.34 (63.57,99.36)	82.02 (65.52,102.3)	0.16 (0.11, 0.20)
Ukraine	51426 (40914,64911)	57499 (45252,72876)	75.95 (60.57,95.16)	82.14 (64.95,102.41)	0.28 (0.26, 0.29)
United Arab Emirates	1112 (854,1447)	13866 (10073,18535)	105.85 (84.78,132.56)	139.39 (109.88,178.47)	0.94 (0.91, 0.97)
United Kingdom	64856 (50928,81060)	95947 (75179,119798)	84.06 (66.25,105.31)	91.63 (72.24,114.25)	0.50 (0.37, 0.63)
United Republic of Tanzania	10125 (7951,12574)	27151 (21582,33941)	82.18 (64.31,103.91)	88.16 (70.86,110.87)	0.26 (0.22, 0.30)
United States of America	391130 (309495,485362)	909975 (729320,1112770)	131.81 (104.33,161.82)	191.95 (155.54,231.03)	1.89 (1.63, 2.14)
United States Virgin Islands	39 (31,49)	76 (61,97)	40.91 (32.97,50.79)	53.34 (42.96,66.03)	0.87 (0.83, 0.91)
Uruguay	3869 (3043,4914)	5733 (4523,7174)	107.87 (84.87,136.5)	123.58 (97.42,152.99)	0.38 (0.35, 0.42)
Uzbekistan	9303 (7449,11472)	25509 (20030,31607)	73.36 (57.81,92.31)	85.02 (67.71,105.71)	0.49 (0.47, 0.51)
Vanuatu	98 (77,122)	280 (220,350)	126.35 (100.81,159.61)	134.29 (106.93,167.78)	0.21 (0.20, 0.22)
Venezuela (Bolivarian Republic of)	3411 (2717,4274)	10190 (8170,12731)	27.04 (21.97,33.76)	34 (27.54,42.29)	0.75 (0.72, 0.77)
Viet Nam	41163 (33205,50659)	118704 (92858,148049)	93.09 (74.5,116.37)	109.77 (86.64,137.5)	0.64 (0.59, 0.69)
Yemen	4209 (3339,5253)	14369 (11464,17776)	71.01 (56.14,88.95)	80.71 (63.68,101.32)	0.47 (0.44, 0.50)
Zambia	2830 (2235,3545)	8038 (6351,10054)	84.66 (67.08,107.78)	88.11 (69.6,111.07)	0.12 (0.08, 0.17)
Zimbabwe	4204 (3294,5218)	7382 (5762,9296)	88.33 (68.8,111.03)	86.93 (68.88,109.66)	-0.09 (-0.13, -0.06)

Table S4. Gout DALYs in 204 countries and territories between 1990 and 2021.

Location	DALYs case No. (95% UI)		ASDR per 100,000 No. (95% UI)		1990-2021EA PC in ASDR No. (95% CI)
	1990	2021	1990	2021	
Afghanistan	818 (525,1225)	1612 (1018,2348)	11.85 (7.77,17.61)	12.77 (8.5,18.18)	0.33 (0.23, 0.43)
Albania	220 (143,321)	441 (298,648)	9.62 (6.31,14.08)	10.94 (7.33,15.83)	0.48 (0.45, 0.50)
Algeria	1786 (1204,2620)	6261 (4138,9069)	13.41 (9.07,19.43)	16.1 (10.8,23.42)	0.64 (0.62, 0.66)
American Samoa	7 (5,11)	15 (10,21)	26.38 (17.6,39.04)	28.82 (19.65,40.71)	0.21 (0.18, 0.24)
Andorra	12 (8,17)	31 (20,45)	19.5 (13.11,29.12)	20.82 (13.88,30.37)	0.20 (0.17, 0.22)
Angola	570 (364,844)	1864 (1195,2726)	12.48 (8.19,18.34)	13 (8.51,18.75)	0.18 (0.14, 0.21)
Antigua and Barbuda	3 (2,5)	9 (5,12)	6.05 (3.79,8.8)	7.86 (5.12,11.31)	0.89 (0.85, 0.92)
Argentina	7411 (4918,10747)	15194 (10352,22080)	22.99 (15.29,33.25)	28.2 (19.23,41.04)	0.64 (0.60, 0.68)
Armenia	335 (217,490)	588 (390,862)	11.61 (7.53,17.16)	14.26 (9.41,20.85)	0.77 (0.72, 0.82)
Australia	6012 (4018,8628)	17652 (11972,25810)	31.16 (20.87,44.97)	43.14 (29.17,63.38)	1.16 (1.05, 1.27)
Austria	1766 (1193,2567)	3078 (2045,4498)	16.49 (11.19,23.87)	19.73 (13.12,28.76)	0.59 (0.56, 0.61)
Azerbaijan	625 (409,903)	1605 (1060,2391)	11.68 (7.78,17)	14.35 (9.57,21.03)	0.79 (0.73, 0.85)
Bahamas	12 (8,18)	35 (22,50)	6.51 (4.29,9.58)	8.01 (5.06,11.59)	0.69 (0.66, 0.72)
Bahrain	43 (27,64)	257 (165,388)	15.75 (10.44,22.72)	18.63 (12.55,27.11)	0.61 (0.58, 0.65)
Bangladesh	6101 (3924,8866)	17810 (11545,25881)	11.57 (7.4,16.66)	12.28 (8.07,17.88)	0.21 (0.20, 0.22)
Barbados	17 (11,25)	36 (24,53)	6.46 (4.21,9.2)	8.09 (5.35,11.77)	0.75 (0.72, 0.78)
Belarus	1381 (914,1976)	1840 (1210,2669)	10.96 (7.26,15.66)	12.37 (8.19,17.88)	0.44 (0.40, 0.47)
Belgium	2399 (1576,3503)	3830 (2553,5670)	17.06 (11.22,24.85)	19.48 (12.96,28.6)	0.43 (0.41, 0.46)
Belize	7 (4,10)	29 (19,43)	6.38 (4.17,9.31)	8.48 (5.54,12.31)	0.91 (0.84, 0.99)
Benin	273 (177,394)	876 (564,1275)	12.49 (8.09,18.06)	14.18 (9.39,20.77)	0.43 (0.41, 0.45)

Bermuda	5 (3,7)	10 (6,14)	6.92 (4.46,10.18)	8.68 (5.66,12.74)	0.76 (0.74, 0.78)
Bhutan	34 (22,50)	88 (58,131)	11.6 (7.64,16.85)	13.47 (8.85,19.85)	0.52 (0.49, 0.56)
Bolivia (Plurinational State of)	251 (163,374)	899 (576,1329)	6.68 (4.33,9.79)	8.9 (5.82,12.95)	0.93 (0.90, 0.97)
Bosnia and Herzegovina	396 (258,569)	604 (389,892)	9.08 (5.96,12.87)	10.82 (7.13,15.81)	0.59 (0.57, 0.62)
Botswana	84 (54,123)	278 (180,404)	13.42 (8.78,19.54)	15.92 (10.65,22.84)	0.58 (0.52, 0.64)
Brazil	6474 (4229,9338)	20204 (13437,28951)	6.29 (4.18,9.09)	7.95 (5.28,11.32)	0.80 (0.77, 0.83)
Brunei Darussalam	29 (18,44)	101 (66,149)	21.29 (13.97,30.72)	23.94 (15.69,34.98)	0.41 (0.37, 0.44)
Bulgaria	1210 (804,1755)	1358 (887,1965)	10.25 (6.82,14.94)	10.99 (7.22,16.14)	0.25 (0.22, 0.29)
Burkina Faso	549 (373,798)	1409 (925,2082)	11.78 (7.99,17.26)	13.13 (8.76,19.16)	0.39 (0.37, 0.41)
Burundi	315 (205,469)	786 (510,1131)	12.27 (8.11,18.12)	13.38 (8.82,19.49)	0.33 (0.31, 0.35)
Cabo Verde	26 (17,38)	71 (45,101)	11.9 (7.81,17.44)	14.44 (9.3,20.8)	0.68 (0.65, 0.72)
Cambodia	780 (512,1108)	2455 (1621,3587)	14.87 (9.88,21.5)	17.47 (11.56,24.95)	0.55 (0.51, 0.59)
Cameroon	700 (460,1023)	2382 (1520,3470)	14.01 (9.35,19.95)	15.58 (10.22,22.55)	0.37 (0.35, 0.39)
Canada	11472 (7780,16534)	28511 (19245,40146)	36.21 (24.57,52.64)	46.64 (31.77,67.31)	0.81 (0.76, 0.86)
Central African Republic	154 (101,222)	330 (209,487)	11.84 (7.79,16.98)	12.11 (7.94,17.18)	0.09 (0.08, 0.11)
Chad	353 (233,506)	889 (561,1297)	11.75 (7.79,16.83)	13.09 (8.51,19.18)	0.41 (0.39, 0.42)
Chile	2592 (1727,3740)	7444 (4992,10709)	24.34 (16.38,35.01)	30.23 (20.23,43.4)	0.66 (0.60, 0.73)
China	190614 (126028,276352)	525967 (353075,758229)	20.2 (13.46,29.14)	25.43 (17.16,36.31)	1.06 (0.94, 1.19)
Colombia	933 (590,1407)	3028 (1978,4474)	4.24 (2.71,6.18)	5.51 (3.59,8.14)	0.82 (0.78, 0.85)
Comoros	29 (18,44)	75 (49,109)	13.23 (8.65,19.72)	13.8 (8.95,20.14)	0.18 (0.17, 0.20)
Congo	149 (96,217)	492 (318,735)	12.75 (8.37,18.12)	15.07 (10.01,22.57)	0.58 (0.55, 0.61)
Cook Islands	3 (2,5)	7 (4,10)	24.42 (16.23,35.37)	27.19 (18,39.71)	0.29 (0.26, 0.31)

Costa Rica	99 (62,151)	320 (210,478)	4.74 (2.97,7.25)	5.86 (3.85,8.8)	0.67 (0.64, 0.69)
Croatia	583 (380,876)	860 (570,1246)	9.59 (6.31,14.14)	11.35 (7.39,16.4)	0.28 (0.25, 0.32)
Cuba	671 (438,995)	1389 (922,2020)	6.43 (4.21,9.56)	8.06 (5.36,11.82)	0.60 (0.58, 0.62)
Cyprus	139 (92,201)	378 (253,551)	16.83 (11.17,24.15)	19.06 (12.75,27.57)	0.76 (0.73, 0.79)
Czechia	1255 (826,1840)	2103 (1373,3102)	9.57 (6.26,14.06)	11.34 (7.44,16.71)	0.37 (0.33, 0.41)
Côte d'Ivoire	666 (415,983)	2033 (1320,2949)	13.45 (8.76,19.63)	14.58 (9.62,20.67)	0.67 (0.47, 0.87)
Democratic People's Republic of Korea	3939 (2587,5813)	8398 (5602,12295)	21.88 (14.44,31.98)	25.14 (16.75,36.19)	0.48 (0.44, 0.52)
Democratic Republic of the Congo	2241 (1414,3268)	5760 (3784,8364)	12.93 (8.49,18.51)	12.99 (8.57,18.73)	0.02 (-0.06, 0.10)
Denmark	1226 (813,1772)	1974 (1311,2946)	16.78 (11.12,24.42)	19.57 (13.06,28.86)	0.53 (0.50, 0.56)
Djibouti	22 (14,34)	116 (75,173)	12.91 (8.37,18.74)	14.63 (9.66,21.4)	0.46 (0.43, 0.49)
Dominica	4 (2,5)	7 (4,10)	6.32 (4.24,9.27)	8.3 (5.33,12.21)	0.90 (0.86, 0.94)
Dominican Republic	256 (164,387)	810 (534,1187)	5.89 (3.78,8.67)	7.67 (5.07,11.17)	0.94 (0.90, 0.98)
Ecuador	468 (297,681)	1661 (1105,2408)	7.59 (4.92,10.75)	9.75 (6.5,14.16)	1.08 (0.98, 1.18)
Egypt	4344 (2781,6229)	12679 (8144,18655)	14.09 (9.24,20.26)	17.67 (11.52,25.72)	0.69 (0.67, 0.71)
El Salvador	144 (93,212)	343 (225,502)	4.25 (2.77,6.22)	5.52 (3.63,8.09)	0.81 (0.78, 0.85)
Equatorial Guinea	25 (16,36)	102 (66,151)	11.69 (7.77,16.79)	15.38 (10.31,22.44)	1.09 (1.00, 1.18)
Eritrea	163 (105,251)	427 (280,623)	11.57 (7.54,17.06)	12.28 (8.22,17.93)	0.19 (0.16, 0.21)
Estonia	223 (152,328)	312 (209,459)	11.25 (7.73,16.54)	13.77 (9.35,20.54)	0.71 (0.68, 0.73)
Eswatini	51 (33,74)	107 (71,153)	15.26 (10.15,21.9)	16.05 (10.74,22.94)	0.10 (0.08, 0.13)
Ethiopia	2917 (1899,4261)	7153 (4736,10410)	13.11 (8.68,18.94)	14.07 (9.32,20.46)	0.23 (0.22, 0.24)
Fiji	99 (64,145)	216 (142,312)	22.23 (15.17,32.28)	25.49 (17,36.84)	0.39 (0.37, 0.41)
Finland	1100 (735,1605)	1979 (1313,2882)	16.37 (10.9,24.06)	19.36 (12.55,28.02)	0.53 (0.50, 0.55)

France	12483 (8269,18345)	21879 (14743,32027)	16.48 (10.9,24.15)	19.05 (12.7,27.84)	0.47 (0.44, 0.50)
Gabon	79 (52,113)	189 (123,277)	13.21 (8.85,18.93)	15.86 (10.59,23.09)	0.61 (0.59, 0.63)
Gambia	54 (35,81)	162 (107,237)	13.17 (8.64,19.21)	14.05 (9.43,20.64)	0.21 (0.20, 0.22)
Georgia	751 (500,1097)	726 (472,1065)	12.16 (8.14,17.7)	13.43 (8.75,19.65)	0.36 (0.32, 0.39)
Germany	19753 (13110,28634)	32022 (21323,46466)	16.97 (11.14,24.75)	20.04 (13.48,29.1)	0.57 (0.55, 0.58)
Ghana	888 (566,1300)	2880 (1878,4256)	12.38 (8.08,18.06)	14.48 (9.66,21.45)	0.49 (0.47, 0.51)
Greece	2785 (1835,4084)	4170 (2781,6034)	19.44 (12.83,28.64)	21.4 (14.18,30.77)	0.63 (0.16, 1.10)
Greenland	15 (10,22)	32 (21,46)	35.09 (23.98,50.58)	44.76 (30.25,63.67)	0.86 (0.80, 0.92)
Grenada	4 (3,6)	9 (6,14)	5.88 (3.76,8.62)	7.87 (5.18,11.66)	0.88 (0.84, 0.92)
Guam	22 (15,32)	53 (36,78)	23.76 (15.73,33.98)	26.22 (17.61,38.66)	0.34 (0.32, 0.35)
Guatemala	175 (106,262)	647 (406,942)	4 (2.51,5.91)	5.18 (3.28,7.5)	0.84 (0.82, 0.86)
Guinea	437 (291,641)	861 (565,1266)	12.56 (8.46,18.51)	13.48 (8.98,19.62)	0.25 (0.22, 0.27)
Guinea-Bissau	54 (35,78)	115 (75,173)	12.13 (8.04,17.51)	12.65 (8.32,18.59)	0.13 (0.12, 0.15)
Guyana	28 (18,40)	53 (34,74)	5.96 (3.9,8.63)	7.62 (5.05,10.72)	0.77 (0.74, 0.79)
Haiti	205 (130,301)	589 (375,861)	5.43 (3.5,7.94)	6.54 (4.27,9.62)	0.62 (0.59, 0.64)
Honduras	104 (68,154)	401 (249,603)	4.1 (2.69,5.97)	5.27 (3.31,7.88)	0.79 (0.75, 0.83)
Hungary	1365 (884,1983)	1904 (1269,2769)	9.79 (6.41,14.18)	11.27 (7.42,16.4)	0.44 (0.43, 0.45)
Iceland	50 (34,72)	108 (72,158)	18.31 (12.29,26.47)	20.76 (13.83,30.39)	0.42 (0.40, 0.45)
India	62529 (41624,90358)	164153 (109106,232910)	11.99 (8.05,17.28)	12.96 (8.67,18.71)	0.24 (0.22, 0.26)
Indonesia	19183 (12575,28009)	56708 (37503,82310)	16.64 (11.21,24.07)	20.71 (13.9,29.78)	0.71 (0.69, 0.74)
Iran (Islamic Republic of)	3985 (2628,5871)	13105 (8652,18876)	13.64 (9.13,19.92)	15.34 (10.37,22.25)	0.35 (0.28, 0.42)
Iraq	1292 (856,1872)	4505 (2916,6828)	14.56 (9.63,21.15)	15.95 (10.88,23.63)	0.31 (0.28, 0.35)

Ireland	704 (477,1022)	1496 (1003,2192)	18.15 (12.4,26.6)	20.52 (13.67,30.4)	0.42 (0.39, 0.44)
Israel	849 (555,1219)	2319 (1549,3344)	18.05 (11.84,25.92)	20.49 (13.82,29.31)	0.40 (0.39, 0.42)
Italy	12690 (8594,18303)	20483 (13934,29662)	15.49 (10.46,22.48)	17.3 (11.79,25.24)	0.44 (0.31, 0.57)
Jamaica	113 (74,165)	257 (165,372)	6.38 (4.19,9.37)	8.26 (5.32,11.9)	0.86 (0.80, 0.91)
Japan	34025 (23048,48871)	60716 (40826,88089)	20.3 (13.76,29.13)	22.72 (15.37,32.65)	0.34 (0.33, 0.36)
Jordan	240 (154,356)	1643 (1073,2385)	14.91 (9.68,21.53)	18.25 (12.11,26.26)	0.71 (0.68, 0.74)
Kazakhstan	1576 (1058,2283)	2658 (1710,3914)	11.78 (7.96,17.04)	14 (9.14,20.45)	0.61 (0.57, 0.66)
Kenya	1290 (866,1854)	4007 (2670,5838)	13.87 (9.33,20.11)	14.72 (9.84,21.18)	0.26 (0.20, 0.32)
Kiribati	9 (6,12)	18 (12,27)	19.92 (13.17,29.09)	21.46 (14.52,31.12)	0.17 (0.14, 0.19)
Kuwait	164 (107,244)	815 (530,1199)	17.76 (11.83,25.66)	19.52 (13.2,28.08)	0.44 (0.38, 0.51)
Kyrgyzstan	354 (229,503)	700 (451,1040)	11.4 (7.39,16.26)	13.03 (8.52,19.12)	0.45 (0.43, 0.47)
Lao People's Democratic Republic	373 (243,542)	1082 (698,1585)	16.01 (10.61,23)	19.8 (12.96,28.74)	0.74 (0.71, 0.77)
Latvia	385 (249,563)	437 (301,636)	11.18 (7.26,16.38)	13.04 (8.82,19.1)	0.54 (0.51, 0.56)
Lebanon	308 (201,459)	936 (634,1362)	13.4 (8.85,20.02)	15.58 (10.49,22.79)	0.50 (0.46, 0.55)
Lesotho	111 (71,163)	172 (111,252)	12.31 (7.95,17.9)	14.13 (9.38,20.45)	0.46 (0.43, 0.49)
Liberia	171 (117,248)	403 (265,594)	13.78 (9.4,20.01)	14.9 (9.95,21.49)	0.44 (0.35, 0.53)
Libya	317 (209,459)	1029 (657,1518)	14.86 (9.81,21.69)	16.51 (10.85,23.94)	0.36 (0.31, 0.40)
Lithuania	499 (334,735)	622 (410,916)	11.35 (7.55,16.68)	12.7 (8.38,18.96)	0.37 (0.35, 0.39)
Luxembourg	89 (60,132)	197 (128,286)	17.23 (11.55,25.4)	20.04 (12.94,28.93)	0.50 (0.48, 0.52)
Madagascar	721 (468,1062)	1829 (1182,2693)	12.97 (8.51,18.84)	13.38 (8.83,19.3)	0.17 (0.12, 0.21)
Malawi	545 (358,792)	1185 (788,1728)	12.59 (8.36,18.36)	13.36 (8.91,19.68)	0.23 (0.21, 0.25)
Malaysia	1985 (1305,2900)	6928 (4504,10238)	18.05 (11.94,26.17)	22.57 (14.88,33)	0.75 (0.74, 0.77)

Maldives	19 (12,29)	112 (72,167)	18.67 (12.08,27.8)	23.43 (15.48,33.76)	0.77 (0.70, 0.85)
Mali	531 (352,764)	1384 (893,2046)	12.16 (8.24,17.53)	13.56 (9.02,19.89)	0.40 (0.37, 0.42)
Malta	72 (48,106)	162 (108,240)	16.89 (11.19,24.78)	19.88 (13.3,29.57)	0.48 (0.44, 0.53)
Marshall Islands	4 (3,6)	9 (6,14)	19.71 (13.23,29.17)	22.64 (15.01,33.96)	0.43 (0.41, 0.44)
Mauritania	145 (97,212)	374 (248,554)	13.41 (9.05,19.64)	15.61 (10.45,23.33)	0.51 (0.48, 0.54)
Mauritius	151 (97,218)	400 (264,585)	18.13 (11.83,26.03)	22.43 (14.89,32.55)	0.69 (0.68, 0.70)
Mexico	2899 (1869,4231)	8772 (5711,12708)	5.58 (3.7,8.13)	6.56 (4.3,9.53)	0.72 (0.62, 0.81)
Micronesia (Federated States of)	12 (8,17)	21 (14,30)	21.83 (14.64,32.13)	24.57 (16.61,35.41)	0.31 (0.29, 0.33)
Monaco	11 (7,16)	16 (11,24)	18.52 (12.41,26.82)	20.68 (13.79,30.39)	0.38 (0.34, 0.41)
Mongolia	136 (88,202)	348 (224,505)	11.89 (7.8,17.58)	12.88 (8.5,18.76)	0.34 (0.31, 0.37)
Montenegro	67 (43,95)	108 (71,160)	10.57 (6.83,15.04)	11.8 (7.8,17.34)	0.42 (0.38, 0.46)
Morocco	1955 (1276,2911)	5460 (3631,7900)	12.72 (8.29,18.79)	15.09 (10.13,21.65)	0.57 (0.54, 0.60)
Mozambique	809 (517,1202)	1695 (1101,2501)	12.14 (7.92,17.84)	12.7 (8.48,18.4)	0.20 (0.17, 0.23)
Myanmar	4124 (2688,6071)	9709 (6429,13980)	15.91 (10.57,23.3)	18.42 (12.43,26.43)	0.53 (0.48, 0.57)
Namibia	99 (64,146)	235 (154,348)	13.89 (9.15,20.21)	14.83 (9.67,21.72)	0.24 (0.21, 0.27)
Nauru	1 (1,2)	2 (1,2)	24.13 (16.07,35.68)	23.76 (15.83,34.37)	-0.10 (-0.21, 0.01)
Nepal	1175 (750,1718)	3029 (2001,4408)	11.14 (7.26,16.11)	12.28 (8.17,17.83)	0.35 (0.34, 0.36)
Netherlands	3430 (2269,5026)	6322 (4221,9406)	18.18 (12.01,26.58)	20.91 (13.94,30.98)	0.19 (0.09, 0.29)
New Zealand	1599 (1076,2291)	3580 (2411,5191)	41.85 (28.14,59.62)	46.67 (31.55,67.23)	0.14 (-0.06, 0.35)
Nicaragua	84 (53,127)	319 (205,469)	4.24 (2.73,6.45)	5.58 (3.66,8.16)	0.86 (0.83, 0.90)
Niger	412 (267,614)	1229 (811,1811)	12.56 (8.32,18.47)	12.81 (8.63,18.85)	0.08 (0.06, 0.09)
Nigeria	6639 (4402,9556)	14260 (9345,20563)	13.58 (8.99,19.39)	12.92 (8.66,18.68)	-0.21 (-0.30, -0.12)

Niue	0 (0,1)	1 (0,1)	22.02 (14.8,32.3)	25.94 (17.23,37.68)	0.52 (0.49, 0.54)
North Macedonia	193 (124,284)	373 (245,539)	9.99 (6.5,14.66)	11.58 (7.54,16.57)	0.52 (0.50, 0.54)
Northern Mariana Islands	8 (5,12)	16 (11,24)	28.44 (18.72,40.8)	28.53 (19.2,42.08)	-0.08 (-0.12, -0.05)
Norway	898 (610,1284)	1527 (1026,2199)	15.15 (10.28,21.8)	17.56 (11.81,25.37)	0.24 (-0.03, 0.51)
Oman	128 (82,191)	597 (372,867)	13.36 (8.77,19.13)	18.01 (11.75,26.28)	0.96 (0.92, 1.01)
Pakistan	6866 (4526,10116)	18359 (12066,27004)	11.26 (7.57,16.42)	13 (8.68,19.01)	0.62 (0.55, 0.70)
Palau	3 (2,4)	7 (4,10)	23.5 (15.88,34.06)	27.8 (18.39,38.99)	0.43 (0.36, 0.50)
Palestine	121 (79,178)	464 (301,668)	12.81 (8.4,18.48)	15.16 (10.07,22.07)	0.45 (0.42, 0.48)
Panama	72 (46,104)	252 (162,363)	4.18 (2.71,6.08)	5.7 (3.67,8.23)	0.97 (0.95, 0.99)
Papua New Guinea	409 (270,589)	1294 (852,1912)	18.97 (12.74,26.72)	20.58 (13.73,30.39)	0.24 (0.23, 0.26)
Paraguay	156 (100,231)	491 (318,722)	6.16 (4.07,9.08)	7.7 (5,11.4)	0.71 (0.69, 0.73)
Peru	939 (609,1389)	3176 (2104,4610)	6.83 (4.55,10.02)	8.96 (5.97,13.03)	0.93 (0.91, 0.96)
Philippines	5527 (3606,8043)	17995 (11794,26047)	15.76 (10.55,22.88)	19.47 (12.92,28.19)	0.68 (0.62, 0.74)
Poland	4263 (2855,6192)	7280 (4843,10542)	9.9 (6.62,14.42)	11.44 (7.62,16.51)	0.45 (0.44, 0.46)
Portugal	2234 (1477,3299)	3975 (2692,5863)	16.89 (11.23,25.02)	19.46 (13.19,28.56)	0.55 (0.46, 0.65)
Puerto Rico	250 (161,366)	498 (334,720)	6.98 (4.5,10.21)	8.92 (5.97,13.01)	0.81 (0.78, 0.84)
Qatar	45 (27,69)	500 (307,734)	18.46 (12.02,27.34)	21.94 (14.68,31.39)	0.68 (0.63, 0.74)
Republic of Korea	6538 (4252,9773)	20002 (13385,29031)	18.87 (12.41,27.82)	22.68 (15.2,33.04)	0.60 (0.58, 0.62)
Republic of Moldova	490 (329,709)	719 (481,1041)	11.03 (7.44,15.94)	12.8 (8.56,18.65)	0.56 (0.51, 0.60)
Romania	2629 (1741,3885)	3697 (2467,5312)	9.64 (6.4,14.04)	11.34 (7.5,16.2)	0.55 (0.52, 0.57)
Russian Federation	20366 (13575,28855)	30076 (20345,43578)	11.45 (7.62,16.39)	13.48 (9.01,19.43)	0.56 (0.54, 0.58)
Rwanda	388 (251,574)	954 (611,1411)	12.25 (8.14,17.79)	12.81 (8.5,18.62)	0.19 (0.13, 0.25)

Saint Kitts and Nevis	2 (1,3)	6 (4,9)	6.38 (4.16,9.48)	8.14 (5.34,11.94)	0.79 (0.76, 0.83)
Saint Lucia	6 (4,8)	19 (12,28)	6.08 (3.8,9.03)	8.03 (5.32,11.67)	0.88 (0.83, 0.93)
Saint Vincent and the Grenadines	4 (3,6)	11 (7,16)	5.89 (3.97,8.57)	7.99 (5.28,11.68)	1.01 (0.97, 1.05)
Samoa	22 (14,33)	40 (27,58)	23.59 (15.56,35.19)	25.77 (17.55,37.29)	0.19 (0.16, 0.22)
San Marino	6 (4,8)	12 (8,18)	18.31 (11.97,26.45)	19.9 (13.25,29.1)	0.27 (0.23, 0.31)
Sao Tome and Principe	9 (6,13)	20 (13,29)	12.91 (8.53,18.92)	15.16 (10.2,22.2)	0.56 (0.54, 0.58)
Saudi Arabia	1131 (741,1662)	5749 (3767,8647)	15.02 (9.92,21.89)	19.81 (13.55,28.49)	0.90 (0.87, 0.92)
Senegal	460 (295,658)	1196 (773,1792)	12.95 (8.55,18.66)	13.61 (8.94,20.66)	0.18 (0.17, 0.20)
Serbia	1130 (737,1671)	1680 (1099,2423)	9.97 (6.51,14.62)	11.5 (7.44,16.64)	0.50 (0.47, 0.52)
Seychelles	11 (7,15)	29 (19,41)	18.49 (12.33,26.76)	22.93 (15.2,32.64)	0.68 (0.66, 0.70)
Sierra Leone	278 (185,412)	589 (379,878)	12.51 (8.37,18.46)	13.24 (8.76,19.23)	0.23 (0.18, 0.29)
Singapore	518 (341,751)	2198 (1480,3247)	20.14 (13.52,29.56)	25.43 (17.11,37.62)	0.79 (0.74, 0.84)
Slovakia	562 (374,815)	961 (628,1436)	9.6 (6.37,13.98)	11.03 (7.14,16.63)	0.41 (0.39, 0.42)
Slovenia	230 (149,339)	430 (287,639)	9.55 (6.22,14.13)	11.47 (7.55,16.93)	0.59 (0.58, 0.60)
Solomon Islands	33 (22,48)	91 (61,135)	20.67 (13.97,30.48)	22.06 (14.95,32.43)	0.15 (0.12, 0.17)
Somalia	374 (241,548)	945 (621,1416)	12.36 (8.31,17.9)	11.76 (7.75,17.44)	-0.14 (-0.17, -0.12)
South Africa	3576 (2424,5196)	8673 (5830,12616)	15.87 (10.79,23.08)	17.23 (11.7,25.06)	0.33 (0.29, 0.37)
South Sudan	399 (267,582)	658 (427,956)	14.31 (9.52,20.77)	14.47 (9.83,20.98)	0.08 (0.06, 0.10)
Spain	8961 (6026,13031)	16611 (11083,24369)	17.65 (11.66,25.51)	20 (13.31,29.35)	0.38 (0.32, 0.43)
Sri Lanka	2183 (1452,3176)	5309 (3587,7753)	17.68 (11.94,25.72)	19.82 (13.32,28.72)	0.40 (0.36, 0.44)
Sudan	1235 (815,1822)	3471 (2308,5084)	12.12 (8.09,17.78)	15.05 (10.11,21.82)	0.75 (0.73, 0.78)
Suriname	18 (11,25)	50 (32,72)	6.25 (4.06,8.91)	7.79 (5.08,11.2)	0.74 (0.72, 0.75)

Sweden	2318 (1506,3366)	3406 (2288,4983)	17.66 (11.49,25.65)	19.1 (12.69,28.05)	-0.06 (-0.18, 0.06)
Switzerland	1628 (1090,2393)	2993 (2009,4335)	17.1 (11.45,25.32)	19.36 (12.84,27.93)	0.42 (0.41, 0.44)
Syrian Arab Republic	818 (540,1216)	2197 (1477,3207)	14 (9.42,20.83)	15.68 (10.4,22.4)	0.38 (0.31, 0.45)
Taiwan (Province of China)	4670 (3127,6696)	12766 (8712,18420)	26.54 (17.78,37.73)	33.16 (22.77,47.99)	0.36 (-0.36, 1.08)
Tajikistan	319 (207,469)	840 (545,1264)	10.89 (7.13,16.09)	12.39 (8.28,18.09)	0.48 (0.44, 0.51)
Thailand	7512 (5032,11271)	22662 (14868,33246)	18.07 (12.16,26.72)	21.85 (14.34,31.55)	0.89 (0.80, 0.98)
Timor-Leste	62 (40,90)	167 (111,242)	16.45 (10.76,23.81)	18.3 (12.27,26.38)	0.38 (0.34, 0.42)
Togo	178 (115,263)	604 (401,862)	12.22 (7.83,17.63)	12.94 (8.66,18.85)	0.16 (0.14, 0.19)
Tokelau	0 (0,0)	0 (0,1)	20.18 (13.3,29.52)	24.53 (16.64,35.81)	0.61 (0.59, 0.64)
Tonga	13 (9,19)	21 (14,31)	22.52 (15.07,32.45)	25.62 (17.33,37.22)	0.30 (0.26, 0.34)
Trinidad and Tobago	61 (39,90)	153 (101,227)	6.56 (4.28,9.64)	8.35 (5.54,12.38)	0.83 (0.80, 0.85)
Tunisia	735 (487,1072)	2105 (1370,3054)	13.6 (9.19,19.62)	15.42 (10.11,22.33)	0.44 (0.42, 0.45)
Turkey	5183 (3394,7447)	15434 (10325,22417)	13.57 (9.01,19.56)	16.08 (10.71,23.32)	0.56 (0.53, 0.60)
Turkmenistan	248 (162,363)	643 (416,961)	11.92 (7.85,17.26)	14.58 (9.52,21.29)	0.71 (0.66, 0.75)
Tuvalu	1 (1,2)	3 (2,4)	18.62 (12.19,27.06)	23.33 (15.62,33.62)	0.68 (0.66, 0.70)
Uganda	902 (592,1340)	2343 (1543,3449)	12.54 (8.28,18.73)	13.15 (8.81,19.16)	0.22 (0.16, 0.27)
Ukraine	8084 (5546,11852)	9183 (6167,13502)	11.82 (8.09,17.22)	12.87 (8.69,18.99)	0.33 (0.30, 0.35)
United Arab Emirates	168 (105,252)	2220 (1359,3406)	17.55 (11.7,25.74)	23.63 (16.12,34.41)	1.03 (0.99, 1.07)
United Kingdom	14327 (9669,20567)	22931 (15567,32884)	17.81 (12.06,25.97)	20.52 (13.89,29.58)	0.78 (0.60, 0.95)
United Republic of Tanzania	1571 (1032,2314)	4249 (2766,6224)	13.17 (9.02,19.2)	14.36 (9.52,20.7)	0.31 (0.27, 0.35)
United States of America	87726 (59389,125740)	256912 (180720,358545)	29.32 (19.77,41.68)	50.95 (35.36,71.75)	2.70 (2.38, 3.01)
United States Virgin Islands	7 (4,10)	13 (8,19)	6.78 (4.36,9.88)	8.76 (5.74,12.88)	0.86 (0.82, 0.91)

Uruguay	847 (568,1252)	1343 (890,1939)	23.01 (15.38,34.04)	27.64 (18.33,39.9)	0.56 (0.53, 0.59)
Uzbekistan	1475 (970,2123)	4170 (2717,6173)	11.95 (7.83,17.49)	14.19 (9.45,20.99)	0.56 (0.54, 0.59)
Vanuatu	16 (10,23)	46 (30,67)	20.88 (13.97,30.59)	22.53 (15,32.64)	0.26 (0.25, 0.27)
Venezuela (Bolivarian Republic of)	542 (343,813)	1681 (1074,2487)	4.44 (2.84,6.69)	5.53 (3.62,8.09)	0.75 (0.72, 0.78)
Viet Nam	6515 (4419,9410)	19631 (12883,28629)	15.14 (10.21,22.01)	18.21 (11.93,26.47)	0.72 (0.66, 0.77)
Yemen	636 (414,950)	2168 (1407,3209)	11.13 (7.34,16.27)	12.69 (8.36,18.53)	0.49 (0.46, 0.53)
Zambia	436 (276,633)	1223 (776,1822)	13.63 (8.85,19.99)	14.17 (9.43,20.94)	0.14 (0.09, 0.20)
Zimbabwe	654 (420,950)	1127 (737,1693)	14.28 (9.35,20.91)	13.85 (9.12,20.45)	-0.13 (-0.17, -0.09)

Table S5 Age-specific numbers of prevalence, incidence, and DALYs of gout by age and gender in 2021

age	Male- Prevalence 95% UI	Female-Prevalence number 95% UI	Male- Incidence number 95% UI	Female-Incidence number 95% UI	Male-DALY number 95% UI	Female-DALY number 95% UI
<5	0.00 (0.00 - 0.00)	0.00 (0.00 - 0.00)	0.00 (0.00 - 0.00)	0.00 (0.00 - 0.00)	0.00 (0.00 - 0.00)	0.00 (0.00 - 0.00)
5 to 9	0.00 (0.00 - 0.00)	0.00 (0.00 - 0.00)	0.00 (0.00 - 0.00)	0.00 (0.00 - 0.00)	0.00 (0.00 - 0.00)	0.00 (0.00 - 0.00)
10 to 14	0.00 (0.00 - 0.00)	0.00 (0.00 - 0.00)	0.00 (0.00 - 0.00)	0.00 (0.00 - 0.00)	0.00 (0.00 - 0.00)	0.00 (0.00 - 0.00)
15 to 19	15383.28 (4996.43 - 32876.33)	7,305.76 (1,527.21 - 15,881.95)	10720.58 (3486.48 - 21771.55)	5111.15 (1096.91 - 11192.76)	540.11 (164.73 - 1221.53)	256.27 (54.03 - 594.77)
20 to 24	147549.31 (86570.01 - 226815.33)	50,643.14 (20,171.27 - 87,981.14)	73394.49 (43083.98 - 105177.67)	21438.37 (10493.15 - 34539.87)	5149.69 (2628.84 - 8549.66)	1776.26 (689.91 - 3371.04)
25 to 29	566118.22 (341496.12 - 829665.66)	148,305.85 (74,733.67 - 234,678.48)	181861.87 (100732.91 - 270778.60)	44762.27 (21334.55 - 72453.29)	19253.04 (9662.74 - 30897.69)	5206.21 (2313.32 - 8962.55)
30 to 34	1303836.30 (822463.77 - 1883842.21)	304,241.02 (169,783.96 - 470,297.22)	330851.39 (224390.40 - 451865.07)	77878.48 (48317.90 - 114792.60)	43867.43 (22148.18 - 69591.83)	10479.67 (5049.79 - 17523.03)
35 to 39	2068803.06 (1375843.68 - 2934861.41)	465,011.55 (291,358.04 - 697,912.49)	445989.35 (247562.63 - 714798.65)	104975.45 (57189.57 - 179604.30)	68695.64 (40270.77 - 109776.80)	15375.24 (8411.54 - 25246.74)
40	2813207.60	625,823.95	542640.65	135541.66	92559.37	20566.79

to 44	(1766375.73 - 4153115.34)	(381,077.53 - 993,864.82)	(350197.90 - 811003.92)	(83257.53 - 205635.19)	(52360.85 - 151020.66)	(11098.05 - 35836.18)
45 to 49	3816075.10 (2496659.83 - 5333832.84)	896,344.54 (565,951.78 - 1,291,631.99)	684579.31 (392235.44 - 1063793.25)	185463.01 (101910.90 - 297432.27)	123936.16 (73464.35 - 199749.91)	29250.70 (15580.43 - 47581.62)
50 to 54	4806286.27 (3023202.57 - 6964930.13)	1,209,271.08 (733,054.43 - 1,815,147.30)	806426.86 (490972.98 - 1169621.20)	241258.56 (147901.68 - 349409.82)	154419.76 (87528.11 - 250885.81)	38767.22 (21491.54 - 62653.92)
55 to 59	5310687.26 (3698615.70 - 7214602.54)	1,493,679.77 (991,839.56 - 2,046,196.81)	842631.70 (467243.60 - 1297210.97)	285989.83 (156741.07 - 436455.51)	167837.49 (104362.36 - 273177.71)	47106.40 (27711.52 - 76623.74)
60 to 64	5097392.40 (3410691.17 - 7112655.92)	1,598,206.16 (1,024,699.04 - 2,282,609.34)	768200.51 (475153.80 - 1114022.59)	293698.72 (187941.94 - 423356.57)	158088.93 (91275.08 - 253840.78)	49232.85 (27598.95 - 80078.43)
65 to 69	5199507.70 (3481566.18 - 7294883.75)	1,899,022.01 (1,262,011.05 - 2,671,360.10)	763133.83 (438043.07 - 1239987.98)	327695.42 (187370.83 - 538635.22)	158102.12 (94907.61 - 236854.20)	57138.20 (33572.56 - 85592.38)
70 to 74	4471572.88 (2960321.24 - 6554332.66)	1,870,961.12 (1,219,689.93 - 2,758,545.57)	608207.31 (383708.52 - 935008.70)	286433.94 (187096.80 - 433640.42)	133058.19 (79969.48 - 206194.18)	55104.82 (32508.39 - 85379.95)
75 to 79	3064215.19 (2104057.69 - 4231672.36)	1,465,796.29 (984,317.09 - 2,061,149.19)	399828.62 (246721.40 - 614597.35)	203342.66 (125281.23 - 314280.96)	89074.07 (55111.52 - 134752.28)	42229.05 (25905.22 - 63510.56)
80 to 84	2008561.83 (1322369.43 - 2838177.81)	1,152,276.70 (748,163.71 - 1,646,636.05)	255458.80 (166423.37 - 384011.55)	150197.05 (97651.41 - 227535.71)	57085.00 (33431.09 - 89138.16)	32430.25 (19238.31 - 51103.96)
85 to 89	994859.68 (711655.55 - 1379186.84)	712,374.09 (504,572.59 - 985,938.51)	123757.14 (73288.72 - 192500.52)	87811.35 (51396.25 - 136504.35)	27581.67 (17577.69 - 42392.36)	19538.94 (12436.80 - 30069.21)
90 to 94	351170.04 (239285.11 - 516764.58)	329,303.12 (222,549.94 - 487,142.52)	44763.68 (25677.27 - 69274.54)	40245.59 (22827.12 - 62932.36)	9514.10 (5880.29 - 15146.11)	8793.53 (5434.98 - 14082.91)
95 plus	94715.92 (60185.15 - 146974.04)	116,064.29 (72,819.63 - 182,143.24)	12720.30 (5953.94 - 23875.49)	14575.59 (6792.82 - 27764.41)	2511.78 (1457.42 - 4068.88)	3019.32 (1751.74 - 5015.47)

Table S6 Age-specific rates of prevalence, incidence, and DALYs of gout by gender from 1990 to 2021

year	Male-ASPR 95% UI	Female-ASPR 95% UI	Male-ASIR 95% UI	Female-ASIR 95% UI	Male-ASDR 95% UI	Female-ASDR 95% UI
1990	844.63 (678.40 - 1049.92)	260.55 (209.31 - 324.61)	143.14 (114.58 - 177.70)	47.11 (37.84 - 58.91)	26.25 (17.72 - 37.84)	8.01 (5.41 - 11.66)
1991	837.82 (673.11 - 1040.92)	259.19 (208.31 - 322.88)	142.65 (114.20 - 177.06)	46.92 (37.68 - 58.62)	26.05 (17.52 - 37.52)	7.97 (5.37 - 11.58)

1992	832.33 (669.16 - 1033.58)	257.98 (207.42 - 321.31)	142.25 (113.89 - 176.53)	46.75 (37.52 - 58.35)	25.89 (17.42 - 37.09)	7.94 (5.35 - 11.51)
1993	828.53 (666.55 - 1028.32)	257.01 (206.73 - 319.91)	141.97 (113.62 - 176.24)	46.61 (37.42 - 58.13)	25.79 (17.37 - 37.01)	7.91 (5.35 - 11.51)
1994	826.79 (664.95 - 1025.64)	256.42 (206.32 - 319.00)	141.84 (113.46 - 176.15)	46.53 (37.36 - 57.99)	25.75 (17.38 - 36.95)	7.90 (5.32 - 11.49)
1995	827.55 (665.34 - 1025.91)	256.31 (206.30 - 318.69)	141.87 (113.42 - 176.21)	46.50 (37.34 - 57.95)	25.78 (17.47 - 36.88)	7.89 (5.32 - 11.44)
1996	830.36 (668.09 - 1029.00)	256.52 (206.56 - 318.66)	142.07 (113.57 - 176.38)	46.49 (37.36 - 57.96)	25.87 (17.47 - 36.98)	7.90 (5.34 - 11.56)
1997	834.66 (672.13 - 1034.04)	256.82 (206.90 - 318.99)	142.41 (113.83 - 176.72)	46.49 (37.37 - 57.98)	26.00 (17.49 - 37.27)	7.90 (5.37 - 11.55)
1998	839.83 (676.97 - 1040.01)	257.24 (207.33 - 319.44)	142.85 (114.17 - 177.19)	46.51 (37.41 - 58.03)	26.16 (17.75 - 37.63)	7.92 (5.39 - 11.46)
1999	845.28 (682.09 - 1046.18)	257.79 (207.73 - 320.02)	143.34 (114.58 - 177.73)	46.56 (37.46 - 58.13)	26.33 (17.82 - 37.78)	7.93 (5.35 - 11.56)
2000	850.52 (686.86 - 1052.07)	258.49 (208.14 - 320.80)	143.83 (114.97 - 178.27)	46.65 (37.56 - 58.28)	26.49 (17.88 - 37.97)	7.95 (5.38 - 11.56)
2001	857.44 (692.21 - 1059.47)	260.37 (209.75 - 323.09)	144.66 (115.67 - 179.33)	46.98 (37.85 - 58.61)	26.71 (18.09 - 38.23)	8.01 (5.41 - 11.58)
2002	867.64 (700.78 - 1070.27)	263.95 (212.76 - 327.48)	146.03 (116.79 - 181.10)	47.63 (38.39 - 59.30)	27.03 (18.34 - 38.74)	8.11 (5.49 - 11.77)
2003	879.56 (710.82 - 1083.15)	268.47 (216.54 - 333.17)	147.66 (118.14 - 183.12)	48.43 (39.06 - 60.17)	27.40 (18.67 - 39.25)	8.25 (5.59 - 11.99)
2004	891.70 (720.70 - 1096.18)	273.17 (220.47 - 338.79)	149.29 (119.50 - 185.09)	49.23 (39.71 - 61.06)	27.77 (18.82 - 39.70)	8.39 (5.71 - 12.15)
2005	902.65 (729.71 - 1107.56)	277.28 (223.96 - 343.72)	150.63 (120.64 - 186.69)	49.87 (40.24 - 61.82)	28.11 (19.09 - 40.29)	8.51 (5.78 - 12.29)
2006	915.06 (742.69 - 1120.68)	281.62 (228.08 - 348.67)	151.97 (121.75 - 188.28)	50.45 (40.76 - 62.51)	28.49 (19.45 - 40.64)	8.64 (5.86 - 12.54)
2007	931.12 (758.44 - 1138.03)	286.94 (233.13 - 354.81)	153.62 (123.13 - 190.22)	51.10 (41.35 - 63.30)	28.98 (19.68 - 41.17)	8.80 (6.02 - 12.73)
2008	947.86 (773.88 - 1156.19)	292.25 (238.19 - 360.79)	155.35 (124.55 - 192.23)	51.74 (41.93 - 64.10)	29.51 (20.12 - 41.84)	8.96 (6.09 - 12.95)
2009	962.23 (786.98 - 1171.83)	296.60 (242.35 - 365.42)	156.90 (125.86 - 194.06)	52.28 (42.43 - 64.78)	29.94 (20.35 - 42.59)	9.09 (6.18 - 13.11)
2010	971.63 (796.64 - 1181.93)	299.06 (244.78 - 367.99)	158.04 (126.90 - 195.34)	52.62 (42.77 - 65.23)	30.21 (20.62 - 42.81)	9.17 (6.23 - 13.16)
2011	977.99 (801.21 - 1189.78)	299.96 (245.67 - 369.05)	159.07 (127.62 - 196.67)	52.81 (42.92 - 65.49)	30.42 (20.83 - 43.23)	9.19 (6.25 - 13.21)
2012	985.31 (806.39 - 1199.25)	300.58 (246.29 - 369.69)	160.34 (128.52 - 198.20)	52.98 (43.07 - 65.73)	30.65 (20.90 - 43.38)	9.21 (6.28 - 13.19)
2013	992.82 (811.54 - 1208.96)	301.12 (246.81 - 369.99)	161.66 (129.44 - 199.78)	53.13 (43.16 - 65.97)	30.89 (21.01 - 43.72)	9.23 (6.29 - 13.27)

2014	999.69 (816.04 - 1217.34)	301.76 (247.35 - 370.38)	162.86 (130.25 - 201.30)	53.28 (43.26 - 66.21)	31.10 (21.19 - 44.22)	9.25 (6.30 - 13.31)
2015	1005.23 (819.44 - 1223.79)	302.73 (248.05 - 371.23)	163.72 (130.84 - 202.37)	53.45 (43.37 - 66.46)	31.27 (21.31 - 44.36)	9.28 (6.34 - 13.35)
2016	1011.15 (823.48 - 1233.26)	304.94 (249.35 - 374.13)	164.50 (131.42 - 203.58)	53.77 (43.63 - 66.86)	31.43 (21.41 - 44.58)	9.34 (6.34 - 13.49)
2017	1018.72 (828.36 - 1243.35)	308.54 (251.56 - 379.22)	165.38 (132.09 - 205.11)	54.27 (44.03 - 67.52)	31.66 (21.53 - 44.89)	9.44 (6.43 - 13.63)
2018	1025.51 (832.37 - 1253.03)	312.22 (253.52 - 384.81)	166.16 (132.66 - 206.23)	54.77 (44.40 - 68.19)	31.86 (21.68 - 45.26)	9.55 (6.48 - 13.73)
2019	1029.02 (833.97 - 1261.68)	314.59 (254.58 - 388.86)	166.62 (132.91 - 206.94)	55.08 (44.60 - 68.51)	31.95 (21.68 - 45.36)	9.61 (6.54 - 13.87)
2020	1028.77 (829.25 - 1260.11)	315.37 (254.48 - 389.96)	166.55 (132.38 - 206.94)	55.15 (44.49 - 68.86)	31.92 (21.64 - 45.40)	9.63 (6.52 - 13.95)
2021	1021.84 (819.96 - 1264.60)	315.05 (253.27 - 392.24)	166.22 (132.49 - 207.42)	55.58 (44.79 - 69.44)	31.68 (21.50 - 45.13)	9.61 (6.53 - 13.95)

Table S7 Gender-Based Decomposition Analysis of Gout Prevalence (1990–2021)

Prevalence	Overll difference			Aging			Population			Epidemiological change		
**Location**	**Both**	**Female**	**Male**	**Both**	**Female**	**Male**	**Both**	**Female**	**Male**	**Both**	**Female**	**Male**
Andean Latin America	128981.53	37357.78	91623.75	26247.61 (20.35%)	8099.03 (21.68%)	17281.73 (18.86%)	73756.08 (57.18%)	21348.06 (57.14%)	52802.12 (57.63%)	28977.84 (22.47%)	7910.69 (21.18%)	21539.9 (23.51%)
Australasia	450315.09	98588.22	351726.87	119346.43 (26.5%)	28093.16 (28.5%)	99512.56 (28.29%)	204971.19 (45.52%)	47098.78 (47.77%)	157468.92 (44.77%)	125997.47 (27.98%)	23396.28 (23.73%)	94745.39 (26.94%)
Caribbean	77031.98	26630.34	50401.64	21861.5 (28.38%)	8084.79 (30.36%)	13294.39 (26.38%)	35312.83 (45.84%)	12063.45 (45.3%)	23306.59 (46.24%)	19857.65 (25.78%)	6482.1 (24.34%)	13800.66 (27.38%)
Central Asia	204702.77	51045.84	153656.93	42483.16 (20.75%)	8387.78 (16.43%)	38491.72 (25.05%)	117203.08 (57.26%)	32254.45 (63.19%)	86880.2 (56.54%)	45016.54 (21.99%)	10403.61 (20.38%)	28285 (18.41%)
Central Europe	260479.29	84709.54	175769.75	164774.92 (63.26%)	55677.7 (65.73%)	118368.94 (67.34%)	11935.52 (4.58%)	4062.51 (4.8%)	7279.29 (4.14%)	83768.84 (32.16%)	24969.33 (29.48%)	50121.52 (28.52%)
Central Latin America	337746.51	131021.99	206724.52	91741.51 (27.16%)	37210.11 (28.4%)	52773.62 (25.53%)	184492.07 (54.62%)	71202.99 (54.34%)	112315.58 (54.33%)	61512.93 (18.21%)	22608.89 (17.26%)	41635.32 (20.14%)
Central Sub-Saharan Africa	174608.53	47813.26	126795.27	-4165.98 (-2.39%)	-530.92 (-1.11%)	-4871.43 (-3.84%)	170953.09 (97.91%)	46213.16 (96.65%)	125770.44 (99.19%)	7821.42 (4.48%)	2131.02 (4.46%)	5896.26 (4.65%)
East Asia	11220525.56	2978520.04	8242005.52	5327261.19 (47.48%)	1500876.03 (50.39%)	3840058.65 (46.59%)	3350827.97 (29.86%)	876149.56 (29.42%)	2412233.12 (29.27%)	2542436.4 (22.66%)	601494.45 (20.19%)	1989713.75 (24.14%)
Eastern Europe	395194.06	128709.75	266484.32	245054.28 (62.01%)	77396.46 (60.13%)	214147.98 (80.36%)	-26037.87 (-6.59%)	-8787.75 (-6.83%)	-17230.7 (-6.47%)	176177.65 (44.58%)	60101.03 (46.7%)	69567.04 (26.11%)
Eastern Sub-Saharan Africa	529639.32	149512	380127.31	-23174.5 (-4.38%)	-619.86 (-0.41%)	-32847.7 (-8.64%)	516940.15 (97.6%)	135700.49 (90.76%)	382657.02 (100.67%)	35873.66 (6.77%)	14431.37 (9.65%)	30317.99 (7.98%)
Global	34210057.02	8743692.1	25466364.92	9026645.97 (26.39%)	2391214.4 (27.35%)	6925800.38 (27.2%)	17891068.8 (52.3%)	4559084.88 (52.14%)	13274684.17 (52.13%)	7292342.26 (21.32%)	1793392.83 (20.51%)	5265880.37 (20.68%)

High SDI	10764295.5	2396946.3	8367349.21	3013936.96 (28%)	797373.98 (33.27%)	2519290.15 (30.11%)	3434052.56 (31.9%)	776076.62 (32.38%)	2770015.57 (33.11%)	4316305.98 (40.1%)	823495.7 (34.36%)	3078043.48 (36.79%)
High-income Asia Pacific	1393990.72	398232.78	995757.93	827679.03 (59.37%)	275766.55 (69.25%)	619122.78 (62.18%)	320935.14 (23.02%)	78640.95 (19.75%)	242810.07 (24.38%)	245376.54 (17.6%)	43825.27 (11%)	133825.09 (13.44%)
High-income North America	6269641.65	1294594.16	4975047.49	1273525.03 (20.31%)	298843.32 (23.08%)	1085637.33 (21.82%)	1910754.43 (30.48%)	439319.28 (33.93%)	1503021.12 (30.21%)	3085362.19 (49.21%)	556431.56 (42.98%)	2386389.04 (47.97%)
High-middle SDI	7475154.99	1940532.52	5534622.47	2690044.3 (35.99%)	717035.63 (36.95%)	2126801.09 (38.43%)	2616166.83 (35%)	657956.56 (33.91%)	1993905.85 (36.03%)	2168943.87 (29.02%)	565540.34 (29.14%)	1413915.53 (25.55%)
Low SDI	1422881.97	391998.45	1030883.52	-63157.71 (-4.44%)	-6688.2 (-1.71%)	-73402.96 (-7.12%)	1402606.67 (98.58%)	364887.04 (93.08%)	1031424.75 (100.05%)	83433.02 (5.86%)	33799.61 (8.62%)	72861.72 (7.07%)
Low-middle SDI	4031564.57	1150485.92	2881078.65	741097.01 (18.38%)	234632.61 (20.39%)	450216.97 (15.63%)	2862678.15 (71.01%)	769370.44 (66.87%)	2057385.06 (71.41%)	427789.41 (10.61%)	146482.87 (12.73%)	373476.61 (12.96%)
Middle SDI	10500304.48	2859398.4	7640906.08	3863000.61 (36.79%)	1079878.47 (37.77%)	2760679.12 (36.13%)	4984960.12 (47.47%)	1321618.42 (46.22%)	3590353.33 (46.99%)	1652343.74 (15.74%)	457901.51 (16.01%)	1289873.64 (16.88%)
North Africa and Middle East	1860747.26	470082.4	1390664.85	344813.97 (18.53%)	89908.26 (19.13%)	251104 (18.06%)	1212991.94 (65.19%)	299008.6 (63.61%)	926345.58 (66.61%)	302941.34 (16.28%)	81165.55 (17.27%)	213215.27 (15.33%)
Oceania	39355.05	9201.51	30153.55	4690.68 (11.92%)	1064.53 (11.57%)	3586.88 (11.9%)	31677.26 (80.49%)	7194.6 (78.19%)	24367.18 (80.81%)	2987.11 (7.59%)	942.37 (10.24%)	2199.49 (7.29%)
South Asia	4111112.18	1185534.07	2925578.11	847813.74 (20.62%)	260808.22 (22%)	530781.65 (18.14%)	2928579.44 (71.24%)	788741.18 (66.53%)	2081101.09 (71.13%)	334719 (8.14%)	135984.67 (11.47%)	313695.38 (10.72%)
Southeast Asia	3021399.98	706351.47	2315048.52	875504.16 (28.98%)	222709.85 (31.53%)	639315.63 (27.62%)	1580383.46 (52.31%)	357198.5 (50.57%)	1246065.7 (53.82%)	565512.36 (18.72%)	126443.11 (17.9%)	429667.19 (18.56%)
Southern Latin America	430093.02	95614.34	334478.68	85452.41 (19.87%)	24830.14 (25.97%)	62698.84 (18.75%)	228053.56 (53.02%)	49446.47 (51.71%)	178656.65 (53.41%)	116587.05 (27.11%)	21337.73 (22.32%)	93123.19 (27.84%)
Southern Sub-Saharan Africa	197643.81	57923.53	139720.28	44437.49 (22.48%)	13656.62 (23.58%)	27240.54 (19.5%)	131304.5 (66.43%)	35457.05 (61.21%)	97061.53 (69.47%)	21901.82 (11.08%)	8809.86 (15.21%)	15418.21 (11.04%)

Tropical Latin America	455184.64	173019.84	282164.8	132548.48 (29.12%)	50440.46 (29.15%)	80808.86 (28.64%)	226238.88 (49.7%)	83742.98 (48.4%)	141103.88 (50.01%)	96397.27 (21.18%)	38836.4 (22.45%)	60252.06 (21.35%)
Western Europe	2059261.52	440883.08	1618378.43	859161.48 (41.72%)	207848.57 (47.14%)	773656.31 (47.8%)	632537.5 (30.72%)	138130.15 (31.33%)	522231.36 (32.27%)	567562.54 (27.56%)	94904.37 (21.53%)	322490.76 (19.93%)
Western Sub-Saharan Africa	592402.55	178346.15	414056.4	-62524.49 (-10.55%)	-9758.25 (-5.47%)	-67490.82 (-16.3%)	639345.65 (107.92%)	170976.67 (95.87%)	452513.49 (109.29%)	15581.4 (2.63%)	17127.72 (9.6%)	29033.73 (7.01%)

Table S8 Gender-Based Decomposition Analysis of Gout Incidence (1990–2021)

**Incidence**	**Overll difference**			**Aging**			**Population**			**Epidemiological change**		
**Location**	**Both**	**Female**	**Male**	**Both**	**Female**	**Male**	**Both**	**Female**	**Male**	**Both**	**Female**	**Male**
Andean Latin America	24673.94	7072.65	17601.29	4583.89 (18.58%)	1393.35 (19.7%)	3042.47 (17.29%)	14581.66 (59.1%)	4193.84 (59.3%)	10465.84 (59.46%)	5508.39 (22.32%)	1485.46 (21%)	4092.98 (23.25%)
Australasia	48223.31	12870.08	35353.23	11703.86 (24.27%)	3531.7 (27.44%)	8497.48 (24.04%)	25364.28 (52.6%)	6691.48 (51.99%)	18628.32 (52.69%)	11155.17 (23.13%)	2646.9 (20.57%)	8227.42 (23.27%)
Caribbean	14495.65	4892.27	9603.38	3818.02 (26.34%)	1381.54 (28.24%)	2349.89 (24.47%)	6942.62 (47.89%)	2330.69 (47.64%)	4623.29 (48.14%)	3735.01 (25.77%)	1180.05 (24.12%)	2630.19 (27.39%)
Central Asia	38660.34	9700.87	28959.48	8032.81 (20.78%)	1701.97 (17.54%)	7038.63 (24.31%)	22863.28 (59.14%)	6225.68 (64.18%)	17022.67 (58.78%)	7764.25 (20.08%)	1773.21 (18.28%)	4898.18 (16.91%)
Central Europe	45240.12	14400.21	30839.9	28248.11 (62.44%)	9477.57 (65.82%)	20497.06 (66.46%)	2281.79 (5.04%)	766.23 (5.32%)	1399.77 (4.54%)	14710.21 (32.52%)	4156.41 (28.86%)	8943.07 (29%)
Central Latin America	64331.43	24393.04	39938.39	15458.84 (24.03%)	6144.44 (25.19%)	9012.21 (22.57%)	36411.05 (56.6%)	13838.17 (56.73%)	22372.25 (56.02%)	12461.54 (19.37%)	4410.44 (18.08%)	8553.93 (21.42%)
Central Sub-Saharan Africa	36398.19	9797.17	26601.02	-579.34 (-1.59%)	-97.53 (-1%)	-625.89 (-2.35%)	35208.42 (96.73%)	9459.05 (96.55%)	25961.13 (97.59%)	1769.11 (4.86%)	435.65 (4.45%)	1265.79 (4.76%)

East Asia	1960801.6	512880.74	1447920.86	895000.28 (45.64%)	255757.34 (49.87%)	642472.8 (44.37%)	631236.62 (32.19%)	162972.94 (31.78%)	456231.01 (31.51%)	434564.7 (22.16%)	94150.46 (18.36%)	349217.05 (24.12%)
Eastern Europe	68863.2	22171.63	46691.57	42987.19 (62.42%)	13618.5 (61.42%)	37467.52 (80.24%)	-5034.95 (-7.31%)	-1672.87 (-7.55%)	-3357.16 (-7.19%)	30910.96 (44.89%)	10226 (46.12%)	12581.2 (26.95%)
Eastern Sub-Saharan Africa	110243.29	30699.45	79543.84	-3758.41 (-3.41%)	-41.89 (-0.14%)	-5517.65 (-6.94%)	106738.64 (96.82%)	27997.56 (91.2%)	79025.59 (99.35%)	7263.06 (6.59%)	2743.78 (8.94%)	6035.9 (7.59%)
Global	5418476.13	1474168.4	3944307.73	1341427.38 (24.76%)	380668.12 (25.82%)	995920.05 (25.25%)	3072877.69 (56.71%)	815062.1 (55.29%)	2248181.02 (57%)	1004171.05 (18.53%)	278438.18 (18.89%)	700206.65 (17.75%)
High SDI	1161169.05	324961.01	836208.04	320073.36 (27.56%)	104915.23 (32.29%)	241880.48 (28.93%)	455207.83 (39.2%)	117881.61 (36.28%)	350654.7 (41.93%)	385887.87 (33.23%)	102164.18 (31.44%)	243672.85 (29.14%)
High-income Asia Pacific	179645.86	58186.71	121459.16	106072.38 (59.05%)	39595.88 (68.05%)	74419.8 (61.27%)	48987.57 (27.27%)	13210.16 (22.7%)	35833.25 (29.5%)	24585.91 (13.69%)	5380.67 (9.25%)	11206.11 (9.23%)
High-income North America	571583.52	163516.52	408067	113027.76 (19.77%)	40818.32 (24.96%)	79427.88 (19.46%)	224291.87 (39.24%)	62776.42 (38.39%)	164874.61 (40.4%)	234263.9 (40.99%)	59921.78 (36.65%)	163764.51 (40.13%)
High-middle SDI	1257134.16	329182.97	927951.18	423458.08 (33.68%)	118635.38 (36.04%)	327203.54 (35.26%)	467753.48 (37.21%)	119707.42 (36.37%)	354190.91 (38.17%)	365922.59 (29.11%)	90840.17 (27.6%)	246556.73 (26.57%)
Low SDI	294567.44	80425.28	214142.16	-11352.4 (-3.85%)	-1226.58 (-1.53%)	-13156.56 (-6.14%)	288281.73 (97.87%)	75003.59 (93.26%)	211982.86 (98.99%)	17638.11 (5.99%)	6648.27 (8.27%)	15315.86 (7.15%)
Low-middle SDI	789824.5	222485.79	567338.71	133712.14 (16.93%)	41936.15 (18.85%)	81845.64 (14.43%)	575342.91 (72.84%)	153756.41 (69.11%)	414331.18 (73.03%)	80769.45 (10.23%)	26793.23 (12.04%)	71161.9 (12.54%)
Middle SDI	1913011.77	516349.9	1396661.87	669472.17 (35%)	188845.41 (36.57%)	476757.05 (34.14%)	963610.09 (50.37%)	253623.53 (49.12%)	695693.22 (49.81%)	279929.5 (14.63%)	73880.97 (14.31%)	224211.6 (16.05%)
North Africa and Middle East	359215.26	89595.83	269619.43	63821.96 (17.77%)	16495.51 (18.41%)	46769.02 (17.35%)	239949.01 (66.8%)	58860.5 (65.7%)	183549.89 (68.08%)	55444.29 (15.43%)	14239.82 (15.89%)	39300.51 (14.58%)
Oceania	7645.49	1764.74	5880.75	843.07 (11.03%)	195.05 (11.05%)	635.89 (10.81%)	6291.17 (82.29%)	1420.18 (80.48%)	4847.82 (82.44%)	511.25 (6.69%)	149.51 (8.47%)	397.04 (6.75%)

South Asia	812890.58	231225.83	581664.75	152669.21 (18.78%)	46605.45 (20.16%)	95924.04 (16.49%)	594214.29 (73.1%)	159177.17 (68.84%)	423085.56 (72.74%)	66007.07 (8.12%)	25443.21 (11%)	62655.14 (10.77%)
Southeast Asia	563652.11	132252.19	431399.92	152855.41 (27.12%)	39825.45 (30.11%)	111226.86 (25.78%)	309408.2 (54.89%)	70267.44 (53.13%)	243609.39 (56.47%)	101388.5 (17.99%)	22159.3 (16.76%)	76563.67 (17.75%)
Southern Latin America	53608.44	14000.43	39608.01	9747.93 (18.18%)	3272.07 (23.37%)	6686.81 (16.88%)	31846.21 (59.41%)	7891.22 (56.36%)	23962.45 (60.5%)	12014.29 (22.41%)	2837.13 (20.26%)	8958.76 (22.62%)
Southern Sub-Saharan Africa	38867.51	11215.05	27652.46	8399.05 (21.61%)	2565.29 (22.87%)	5254.06 (19%)	26254.72 (67.55%)	7064.04 (62.99%)	19434.91 (70.28%)	4213.74 (10.84%)	1585.71 (14.14%)	2963.49 (10.72%)
Tropical Latin America	86938.05	32168.77	54769.28	23335.52 (26.84%)	8655.04 (26.91%)	14463.88 (26.41%)	44812.16 (51.54%)	16252.08 (50.52%)	28270.42 (51.62%)	18790.38 (21.61%)	7261.65 (22.57%)	12034.98 (21.97%)
Western Europe	210369.93	54729.55	155640.38	89378.16 (42.49%)	25463.44 (46.53%)	72972.97 (46.89%)	81861.43 (38.91%)	20252.34 (37%)	64829.93 (41.65%)	39130.34 (18.6%)	9013.77 (16.47%)	17837.48 (11.46%)
Western Sub-Saharan Africa	122128.32	36634.66	85493.66	-10662.57 (-8.73%)	-1411.07 (-3.85%)	-12183.21 (-14.25%)	130430.99 (106.8%)	34915.45 (95.31%)	92280.81 (107.94%)	2359.9 (1.93%)	3130.27 (8.54%)	5396.06 (6.31%)

Table S9 Gender-Based Decomposition Analysis of Gout Incidence (1990–2021)

DALYs	Overll difference			Aging			Population			Epidemiological change		
**Location**	**Both**	**Female**	**Male**	**Both**	**Female**	**Male**	**Both**	**Female**	**Male**	**Both**	**Female**	**Male**
Andean Latin America	4079.23	1193.12	2886.11	810.52 (19.87%)	252.7 (21.18%)	531.29 (18.41%)	2353.89 (57.7%)	687.98 (57.66%)	1678.36 (58.15%)	914.83 (22.43%)	252.44 (21.16%)	676.46 (23.44%)
Australasia	13619.3	2927.63	10691.67	3520.33 (25.85%)	819.74 (28%)	2928.88 (27.39%)	6246.42 (45.86%)	1407.93 (48.09%)	4825.35 (45.13%)	3852.55 (28.29%)	699.96 (23.91%)	2937.43 (27.47%)
Caribbean	2390.74	833.83	1556.91	667.84 (27.93%)	247.53 (29.69%)	406.57 (26.11%)	1119.86 (46.84%)	383.82 (46.03%)	737.83 (47.39%)	603.04 (25.22%)	202.48 (24.28%)	412.51 (26.5%)
Central Asia	6458.48	1585.45	4873.03	1357.94	273.01	1215.84	3693.06	1005.52	2749.22	1407.49	306.93	907.97

				(21.03%)	(17.22%)	(24.95%)	(57.18%)	(63.42%)	(56.42%)	(21.79%)	(19.36%)	(18.63%)
Central Europe	7789.75	2472.11	5317.64	4797.49 (61.59%)	1603.3 (64.86%)	3475.38 (65.36%)	365.82 (4.7%)	124 (5.02%)	223.5 (4.2%)	2626.44 (33.72%)	744.81 (30.13%)	1618.76 (30.44%)
Central Latin America	10709.97	4161.66	6548.31	2832.79 (26.45%)	1157.89 (27.82%)	1623.75 (24.8%)	5923.83 (55.31%)	2296.82 (55.19%)	3596.5 (54.92%)	1953.35 (18.24%)	706.95 (16.99%)	1328.06 (20.28%)
Central Sub-Saharan Africa	5518.25	1496.19	4022.06	-114.82 (-2.08%)	-18.71 (-1.25%)	-127.15 (-3.16%)	5345.94 (96.88%)	1445.96 (96.64%)	3932.18 (97.77%)	287.13 (5.2%)	68.94 (4.61%)	217.03 (5.4%)
East Asia	347908	90582.73	257325.26	163298.73 (46.94%)	45135.04 (49.83%)	118442.23 (46.03%)	105731.54 (30.39%)	27107.15 (29.93%)	76607.32 (29.77%)	78877.73 (22.67%)	18340.54 (20.25%)	62275.71 (24.2%)
Eastern Europe	11762.5	3737.51	8024.99	7199.81 (61.21%)	2243.99 (60.04%)	6321.89 (78.78%)	-797.12 (-6.78%)	-266.74 (-7.14%)	-529.65 (-6.6%)	5359.81 (45.57%)	1760.26 (47.1%)	2232.75 (27.82%)
Eastern Sub-Saharan Africa	16778.59	4681.61	12096.98	-680.38 (-4.06%)	-18.58 (-0.4%)	-971.6 (-8.03%)	16243.1 (96.81%)	4253.73 (90.86%)	12034.04 (99.48%)	1215.87 (7.25%)	446.45 (9.54%)	1034.54 (8.55%)
Global	1050005.54	263223.28	786782.25	271984.83 (25.9%)	70940.06 (26.95%)	209282.91 (26.6%)	556540.52 (53%)	139586.76 (53.03%)	415124.05 (52.76%)	221480.18 (21.09%)	52696.46 (20.02%)	162375.29 (20.64%)
High SDI	322603.59	69463.49	253140.1	88371.62 (27.39%)	22974.7 (33.07%)	74215.96 (29.32%)	105049.81 (32.56%)	23180.61 (33.37%)	85365.61 (33.72%)	129182.16 (40.04%)	23308.17 (33.55%)	93558.53 (36.96%)
High-income Asia Pacific	41907.26	11734.2	30173.05	24198.47 (57.74%)	7973.91 (67.95%)	18261.53 (60.52%)	9925.84 (23.69%)	2379.85 (20.28%)	7560 (25.06%)	7782.94 (18.57%)	1380.44 (11.76%)	4351.53 (14.42%)
High-income North America	186243.85	36793.35	149450.5	37291.7 (20.02%)	8669.93 (23.56%)	31760.71 (21.25%)	57885.22 (31.08%)	12964.28 (35.24%)	45903.34 (30.71%)	91066.94 (48.9%)	15159.14 (41.2%)	71786.45 (48.03%)
High-middle SDI	230992.72	58696.82	172295.89	81607.27 (35.33%)	21386.16 (36.43%)	64795.62 (37.61%)	81734.39 (35.38%)	20205.61 (34.42%)	62657.78 (36.37%)	67651.05 (29.29%)	17105.06 (29.14%)	44842.49 (26.03%)
Low SDI	44885.9	12238.6	32647.3	-1930.78 (-4.3%)	-212.63 (-1.74%)	-2233.41 (-6.84%)	43885.28 (97.77%)	11384.74 (93.02%)	32304.26 (98.95%)	2931.4 (6.53%)	1066.49 (8.71%)	2576.45 (7.89%)
Low-middle SDI	125306.74	35365.45	89941.29	22332.81	6984.24	13655.36	89412.38	23866.15	64419.58	13561.55	4515.06	11866.35

				(17.82%)	(19.75%)	(15.18%)	(71.35%)	(67.48%)	(71.62%)	(10.82%)	(12.77%)	(13.19%)
Middle SDI	325727.9	87327.64	238400.26	118300.01 (36.32%)	32575.81 (37.3%)	84927.63 (35.62%)	156988.73 (48.2%)	41043.51 (47%)	113608.55 (47.65%)	50439.15 (15.49%)	13708.32 (15.7%)	39864.08 (16.72%)
North Africa and Middle East	57816.99	14408.2	43408.79	10716.38 (18.53%)	2751.7 (19.1%)	7854.9 (18.1%)	38019.25 (65.76%)	9283.69 (64.43%)	29127.24 (67.1%)	9081.36 (15.71%)	2372.82 (16.47%)	6426.64 (14.8%)
Oceania	1239.34	286.5	952.84	146.34 (11.81%)	32.98 (11.51%)	111.72 (11.72%)	1000.72 (80.75%)	225.21 (78.61%)	771.83 (81%)	92.28 (7.45%)	28.31 (9.88%)	69.29 (7.27%)
South Asia	126734.62	36119.89	90614.73	25094.05 (19.8%)	7620 (21.1%)	15791.85 (17.43%)	90832.29 (71.67%)	24292.38 (67.25%)	64710.63 (71.41%)	10808.28 (8.53%)	4207.51 (11.65%)	10112.25 (11.16%)
Southeast Asia	94891.95	21811.56	73080.39	27072.81 (28.53%)	6784.28 (31.1%)	19892.9 (27.22%)	49969.01 (52.66%)	11175.82 (51.24%)	39523.33 (54.08%)	17850.12 (18.81%)	3851.45 (17.66%)	13664.16 (18.7%)
Southern Latin America	13131.85	2836.3	10295.56	2551.97 (19.43%)	724.85 (25.56%)	1890.98 (18.37%)	7065.81 (53.81%)	1496.89 (52.78%)	5570.52 (54.11%)	3514.07 (26.76%)	614.56 (21.67%)	2834.06 (27.53%)
Southern Sub-Saharan Africa	6016.24	1737.27	4278.97	1371.48 (22.8%)	415.54 (23.92%)	851.07 (19.89%)	4065.24 (67.57%)	1088.78 (62.67%)	3014.36 (70.45%)	579.52 (9.63%)	232.95 (13.41%)	413.55 (9.66%)
Tropical Latin America	14065.38	5360.62	8704.76	3988.75 (28.36%)	1525.56 (28.46%)	2427.56 (27.89%)	7114.54 (50.58%)	2642.55 (49.3%)	4428.71 (50.88%)	2962.08 (21.06%)	1192.51 (22.25%)	1848.49 (21.24%)
Western Europe	62030.19	12820.88	49209.31	25298.97 (40.78%)	5945.55 (46.37%)	22932.21 (46.6%)	19414.18 (31.3%)	4130.18 (32.21%)	16154.27 (32.83%)	17317.05 (27.92%)	2745.15 (21.41%)	10122.83 (20.57%)
Western Sub-Saharan Africa	18913.06	5642.68	13270.38	-1851.37 (-9.79%)	-258.6 (-4.58%)	-2076.88 (-15.65%)	20144.16 (106.51%)	5359.43 (94.98%)	14285.22 (107.65%)	620.28 (3.28%)	541.85 (9.6%)	1062.05 (8%)

Table S10 Frontier DALYs, and effective difference by country or territory in 2021.

Location	SDI	ASDR 2021	Frontier DALYs	Effective difference	Effective difference rank (Age-standardized DALYs rank)
Afghanistan	0.34	12.77(8.5 to 18.18)	4.09	8.68	64 (58)
Albania	0.71	10.94(7.33 to 15.83)	4.07	6.88	43 (35)
Algeria	0.66	16.1(10.8 to 23.42)	4.05	12.05	124 (124)
American Samoa	0.72	28.82(19.65 to 40.71)	4.05	24.78	197 (197)
Andorra	0.87	20.82(13.88 to 30.37)	4.06	16.76	163 (163)
Angola	0.45	13(8.51 to 18.75)	4.06	8.94	74 (68)
Antigua and Barbuda	0.75	7.86(5.12 to 11.31)	4.08	3.77	23 (15)
Argentina	0.72	28.2(19.23 to 41.04)	4.06	24.14	195 (195)
Armenia	0.70	14.26(9.41 to 20.85)	4.06	10.20	96 (95)
Australia	0.84	43.14(29.17 to 63.38)	4.10	39.04	200 (200)
Austria	0.85	19.73(13.12 to 28.76)	4.06	15.68	147 (147)
Azerbaijan	0.69	14.35(9.57 to 21.03)	4.07	10.28	98 (96)
Bahamas	0.81	8.01(5.06 to 11.59)	4.07	3.94	28 (19)
Bahrain	0.75	18.63(12.55 to 27.11)	4.11	14.52	136 (136)
Bangladesh	0.49	12.28(8.07 to 17.88)	4.07	8.21	58 (49)
Barbados	0.75	8.09(5.35 to 11.77)	4.11	3.98	29 (22)
Belarus	0.78	12.37(8.19 to 17.88)	4.07	8.31	59 (52)
Belgium	0.85	19.48(12.96 to 28.6)	4.08	15.40	143 (144)
Belize	0.61	8.48(5.54 to 12.31)	4.05	4.43	35 (27)
Benin	0.37	14.18(9.39 to 20.77)	4.11	10.07	93 (93)

Bermuda	0.82	8.68(5.66 to 12.74)	4.08	4.61	36 (28)
Bhutan	0.47	13.47(8.85 to 19.85)	4.08	9.40	82 (80)
Bolivia (Plurinational State of)	0.60	8.9(5.82 to 12.95)	4.06	4.84	38 (30)
Bosnia and Herzegovina	0.72	10.82(7.13 to 15.81)	4.06	6.76	42 (34)
Botswana	0.64	15.92(10.65 to 22.84)	4.05	11.87	120 (120)
Brazil	0.65	7.95(5.28 to 11.32)	4.06	3.89	25 (17)
Brunei Darussalam	0.81	23.94(15.69 to 34.98)	4.09	19.86	181 (181)
Bulgaria	0.77	10.99(7.22 to 16.14)	4.08	6.91	44 (36)
Burkina Faso	0.29	13.13(8.76 to 19.16)	11.14	1.99	13 (73)
Burundi	0.29	13.38(8.82 to 19.49)	11.12	2.26	14 (77)
Cabo Verde	0.53	14.44(9.3 to 20.8)	4.05	10.39	99 (98)
Cambodia	0.47	17.47(11.56 to 24.95)	4.08	13.39	128 (128)
Cameroon	0.48	15.58(10.22 to 22.55)	4.06	11.52	117 (115)
Canada	0.87	46.64(31.77 to 67.31)	4.06	42.58	202 (202)
Central African Republic	0.31	12.11(7.94 to 17.18)	11.12	0.99	2 (48)
Chad	0.24	13.09(8.51 to 19.18)	11.13	1.96	12 (72)
Chile	0.77	30.23(20.23 to 43.4)	4.03	26.19	198 (198)
China	0.72	25.43(17.16 to 36.31)	4.05	21.38	186 (186)
Colombia	0.66	5.51(3.59 to 8.14)	4.07	1.44	7 (3)
Comoros	0.48	13.8(8.95 to 20.14)	4.07	9.74	87 (86)
Congo	0.58	15.07(10.01 to 22.57)	4.06	11.01	108 (108)
Cook Islands	0.78	27.19(18 to 39.71)	4.06	23.12	192 (192)

Costa Rica	0.70	5.86(3.85 to 8.8)	4.07	1.78	11 (8)
Coted'Ivoire	0.43	14.58(9.62 to 20.67)	4.04	10.54	102 (101)
Croatia	0.80	11.35(7.39 to 16.4)	4.07	7.27	48 (41)
Cuba	0.67	8.06(5.36 to 11.82)	4.07	3.99	30 (21)
Cyprus	0.84	19.06(12.75 to 27.57)	4.08	14.98	137 (138)
Czechia	0.83	11.34(7.44 to 16.71)	4.06	7.29	49 (40)
Democratic People's Republic of Korea	0.57	25.14(16.75 to 36.19)	4.07	21.07	184 (184)
Democratic Republic of the Congo	0.38	12.99(8.57 to 18.73)	4.05	8.94	73 (67)
Denmark	0.90	19.57(13.06 to 28.86)	4.07	15.50	146 (146)
Djibouti	0.49	14.63(9.66 to 21.4)	4.05	10.57	103 (103)
Dominica	0.75	8.3(5.33 to 12.21)	4.11	4.19	32 (25)
Dominican Republic	0.62	7.67(5.07 to 11.17)	4.09	3.58	20 (12)
Ecuador	0.66	9.75(6.5 to 14.16)	4.10	5.65	41 (33)
Egypt	0.61	17.67(11.52 to 25.72)	4.06	13.60	130 (130)
El Salvador	0.56	5.52(3.63 to 8.09)	4.09	1.43	6 (4)
Equatorial Guinea	0.66	15.38(10.31 to 22.44)	4.08	11.30	113 (113)
Eritrea	0.40	12.28(8.22 to 17.93)	4.10	8.18	56 (51)
Estonia	0.84	13.77(9.35 to 20.54)	4.06	9.72	86 (85)
Eswatini	0.59	16.05(10.74 to 22.94)	4.09	11.96	122 (122)
Ethiopia	0.36	14.07(9.32 to 20.46)	4.12	9.95	91 (90)
Fiji	0.68	25.49(17 to 36.84)	4.03	21.46	187 (187)
Finland	0.86	19.36(12.55 to 28.02)	4.11	15.24	140 (140)

France	0.84	19.05(12.7 to 27.84)	4.05	15.00	138 (137)
Gabon	0.63	15.86(10.59 to 23.09)	4.06	11.79	119 (119)
Gambia	0.41	14.05(9.43 to 20.64)	4.11	9.94	89 (89)
Georgia	0.73	13.43(8.75 to 19.65)	4.06	9.37	81 (79)
Germany	0.90	20.04(13.48 to 29.1)	4.10	15.94	153 (154)
Ghana	0.56	14.48(9.66 to 21.45)	4.06	10.42	100 (100)
Greece	0.79	21.4(14.18 to 30.77)	4.06	17.34	165 (165)
Greenland	0.83	44.76(30.25 to 63.67)	4.05	40.71	201 (201)
Grenada	0.67	7.87(5.18 to 11.66)	4.06	3.82	24 (16)
Guam	0.80	26.22(17.61 to 38.66)	4.06	22.16	191 (191)
Guatemala	0.54	5.18(3.28 to 7.5)	4.10	1.07	4 (1)
Guinea	0.34	13.48(8.98 to 19.62)	4.06	9.41	83 (81)
Guinea-Bissau	0.35	12.65(8.32 to 18.59)	4.06	8.59	62 (54)
Guyana	0.65	7.62(5.05 to 10.72)	4.06	3.56	19 (11)
Haiti	0.45	6.54(4.27 to 9.62)	4.08	2.46	16 (9)
Honduras	0.51	5.27(3.31 to 7.88)	4.07	1.20	5 (2)
Hungary	0.79	11.27(7.42 to 16.4)	4.06	7.21	46 (38)
Iceland	0.88	20.76(13.83 to 30.39)	4.07	16.69	162 (162)
India	0.58	12.96(8.67 to 18.71)	4.11	8.85	70 (66)
Indonesia	0.66	20.71(13.9 to 29.78)	4.08	16.63	161 (161)
Iran (Islamic Republic of)	0.70	15.34(10.37 to 22.25)	4.08	11.26	112 (112)
Iraq	0.66	15.95(10.88 to 23.63)	4.01	11.94	121 (121)

Ireland	0.87	20.52(13.67 to 30.4)	4.05	16.47	158 (158)
Israel	0.81	20.49(13.82 to 29.31)	4.06	16.43	157 (156)
Italy	0.81	17.3(11.79 to 25.24)	4.11	13.19	127 (127)
Jamaica	0.68	8.26(5.32 to 11.9)	4.06	4.20	33 (24)
Japan	0.87	22.72(15.37 to 32.65)	4.09	18.63	175 (175)
Jordan	0.73	18.25(12.11 to 26.26)	4.06	14.20	133 (133)
Kazakhstan	0.73	14(9.14 to 20.45)	4.06	9.94	90 (88)
Kenya	0.52	14.72(9.84 to 21.18)	4.07	10.65	104 (104)
Kiribati	0.53	21.46(14.52 to 31.12)	4.08	17.39	166 (166)
Kuwait	0.85	19.52(13.2 to 28.08)	4.06	15.46	145 (145)
Kyrgyzstan	0.60	13.03(8.52 to 19.12)	4.08	8.95	75 (70)
Lao People's Democratic Republic	0.49	19.8(12.96 to 28.74)	4.11	15.69	148 (148)
Latvia	0.83	13.04(8.82 to 19.1)	4.07	8.98	76 (71)
Lebanon	0.74	15.58(10.49 to 22.79)	4.07	11.51	116 (116)
Lesotho	0.51	14.13(9.38 to 20.45)	4.06	10.06	92 (91)
Liberia	0.35	14.9(9.95 to 21.49)	4.08	10.82	106 (106)
Libya	0.73	16.51(10.85 to 23.94)	4.10	12.41	125 (125)
Lithuania	0.86	12.7(8.38 to 18.96)	4.11	8.59	61 (57)
Luxembourg	0.88	20.04(12.94 to 28.93)	4.05	15.99	155 (155)
Madagascar	0.40	13.38(8.83 to 19.3)	4.06	9.32	80 (78)
Malawi	0.38	13.36(8.91 to 19.68)	4.06	9.30	79 (76)
Malaysia	0.74	22.57(14.88 to 33)	4.08	18.49	172 (172)

Maldives	0.65	23.43(15.48 to 33.76)	4.06	19.38	178 (178)
Mali	0.27	13.56(9.02 to 19.89)	11.13	2.43	15 (83)
Malta	0.80	19.88(13.3 to 29.57)	4.01	15.87	152 (151)
Marshall Islands	0.57	22.64(15.01 to 33.96)	4.10	18.54	173 (173)
Mauritania	0.50	15.61(10.45 to 23.33)	4.11	11.50	115 (117)
Mauritius	0.72	22.43(14.89 to 32.55)	4.07	18.36	170 (170)
Mexico	0.66	6.56(4.3 to 9.53)	4.07	2.49	17 (10)
Micronesia (Federated States of)	0.59	24.57(16.61 to 35.41)	4.10	20.47	183 (183)
Monaco	0.91	20.68(13.79 to 30.39)	4.07	16.61	160 (160)
Mongolia	0.62	12.88(8.5 to 18.76)	4.06	8.82	69 (63)
Montenegro	0.80	11.8(7.8 to 17.34)	4.08	7.72	55 (47)
Morocco	0.56	15.09(10.13 to 21.65)	4.07	11.03	109 (109)
Mozambique	0.33	12.7(8.48 to 18.4)	5.26	7.44	53 (56)
Myanmar	0.53	18.42(12.43 to 26.43)	4.05	14.37	135 (135)
Namibia	0.62	14.83(9.67 to 21.72)	4.07	10.77	105 (105)
Nauru	0.63	23.76(15.83 to 34.37)	4.08	19.69	180 (180)
Nepal	0.43	12.28(8.17 to 17.83)	4.07	8.21	57 (50)
Netherlands	0.89	20.91(13.94 to 30.98)	4.11	16.80	164 (164)
New Zealand	0.85	46.67(31.55 to 67.23)	4.01	42.67	203 (203)
Nicaragua	0.52	5.58(3.66 to 8.16)	4.08	1.50	9 (6)
Niger	0.17	12.81(8.63 to 18.85)	11.74	1.07	3 (61)
Nigeria	0.50	12.92(8.66 to 18.68)	4.11	8.81	68 (64)

Niue	0.73	25.94(17.23 to 37.68)	4.01	21.93	190 (190)
North Macedonia	0.75	11.58(7.54 to 16.57)	4.07	7.50	54 (45)
Northern Mariana Islands	0.77	28.53(19.2 to 42.08)	4.12	24.41	196 (196)
Norway	0.92	17.56(11.81 to 25.37)	4.00	13.56	129 (129)
Oman	0.77	18.01(11.75 to 26.28)	4.08	13.93	131 (131)
Pakistan	0.50	13(8.68 to 19.01)	4.07	8.93	72 (69)
Palau	0.75	27.8(18.39 to 38.99)	4.06	23.74	194 (194)
Palestine	0.63	15.16(10.07 to 22.07)	4.05	11.11	111 (110)
Panama	0.71	5.7(3.67 to 8.23)	4.08	1.62	10 (7)
Papua New Guinea	0.42	20.58(13.73 to 30.39)	4.10	16.48	159 (159)
Paraguay	0.64	7.7(5 to 11.4)	4.08	3.62	21 (13)
Peru	0.66	8.96(5.97 to 13.03)	4.11	4.86	39 (32)
Philippines	0.65	19.47(12.92 to 28.19)	4.08	15.39	142 (143)
Poland	0.81	11.44(7.62 to 16.51)	4.11	7.33	50 (42)
Portugal	0.74	19.46(13.19 to 28.56)	4.05	15.41	144 (142)
Puerto Rico	0.83	8.92(5.97 to 13.01)	4.06	4.87	40 (31)
Qatar	0.85	21.94(14.68 to 31.39)	4.07	17.87	168 (168)
Republic of Korea	0.89	22.68(15.2 to 33.04)	4.07	18.61	174 (174)
Republic of Moldova	0.73	12.8(8.56 to 18.65)	4.07	8.73	65 (59)
Romania	0.77	11.34(7.5 to 16.2)	4.07	7.27	47 (39)
Russian Federation	0.81	13.48(9.01 to 19.43)	4.04	9.44	84 (82)
Rwanda	0.44	12.81(8.5 to 18.62)	4.05	8.76	66 (60)

Saint Kitts and Nevis	0.75	8.14(5.34 to 11.94)	4.11	4.03	31 (23)
Saint Lucia	0.67	8.03(5.32 to 11.67)	4.09	3.94	27 (20)
Saint Vincent and the Grenadines	0.64	7.99(5.28 to 11.68)	4.07	3.93	26 (18)
Samoa	0.59	25.77(17.55 to 37.29)	4.10	21.67	189 (189)
San Marino	0.89	19.9(13.25 to 29.1)	4.10	15.80	151 (152)
Sao Tome and Principe	0.51	15.16(10.2 to 22.2)	4.07	11.08	110 (111)
Saudi Arabia	0.82	19.81(13.55 to 28.49)	4.06	15.75	150 (149)
Senegal	0.41	13.61(8.94 to 20.66)	4.07	9.54	85 (84)
Serbia	0.79	11.5(7.44 to 16.64)	4.07	7.44	52 (44)
Seychelles	0.73	22.93(15.2 to 32.64)	4.04	18.89	176 (176)
Sierra Leone	0.36	13.24(8.76 to 19.23)	4.07	9.17	78 (75)
Singapore	0.86	25.43(17.11 to 37.62)	4.11	21.32	185 (185)
Slovakia	0.81	11.03(7.14 to 16.63)	4.07	6.97	45 (37)
Slovenia	0.84	11.47(7.55 to 16.93)	4.11	7.36	51 (43)
Solomon Islands	0.43	22.06(14.95 to 32.43)	4.06	18.00	169 (169)
Somalia	0.08	11.76(7.75 to 17.44)	11.74	0.02	1 (46)
South Africa	0.68	17.23(11.7 to 25.06)	4.11	13.12	126 (126)
South Sudan	0.28	14.47(9.83 to 20.98)	11.12	3.35	18 (99)
Spain	0.77	20(13.31 to 29.35)	4.05	15.96	154 (153)
Sri Lanka	0.70	19.82(13.32 to 28.72)	4.11	15.72	149 (150)
Sudan	0.54	15.05(10.11 to 21.82)	4.11	10.94	107 (107)
Suriname	0.63	7.79(5.08 to 11.2)	4.05	3.74	22 (14)

Sweden	0.89	19.1(12.69 to 28.05)	4.07	15.02	139 (139)
Switzerland	0.93	19.36(12.84 to 27.93)	4.06	15.31	141 (141)
Syrian Arab Republic	0.62	15.68(10.4 to 22.4)	4.10	11.57	118 (118)
Taiwan (Province of China)	0.87	33.16(22.77 to 47.99)	4.06	29.10	199 (199)
Tajikistan	0.54	12.39(8.28 to 18.09)	4.07	8.32	60 (53)
Thailand	0.68	21.85(14.34 to 31.55)	4.05	17.80	167 (167)
Timor-Leste	0.44	18.3(12.27 to 26.38)	4.05	14.25	134 (134)
Togo	0.41	12.94(8.66 to 18.85)	4.06	8.88	71 (65)
Tokelau	0.69	24.53(16.64 to 35.81)	4.08	20.46	182 (182)
Tonga	0.63	25.62(17.33 to 37.22)	4.09	21.54	188 (188)
Trinidad and Tobago	0.77	8.35(5.54 to 12.38)	4.09	4.26	34 (26)
Tunisia	0.68	15.42(10.11 to 22.33)	4.05	11.36	114 (114)
Turkey	0.71	16.08(10.71 to 23.32)	4.05	12.03	123 (123)
Turkmenistan	0.68	14.58(9.52 to 21.29)	4.07	10.51	101 (102)
Tuvalu	0.58	23.33(15.62 to 33.62)	4.06	19.26	177 (177)
Uganda	0.42	13.15(8.81 to 19.16)	4.11	9.04	77 (74)
Ukraine	0.76	12.87(8.69 to 18.99)	4.06	8.80	67 (62)
United Arab Emirates	0.85	23.63(16.12 to 34.41)	4.03	19.61	179 (179)
United Kingdom	0.86	20.52(13.89 to 29.58)	4.12	16.40	156 (157)
United Republic of Tanzania	0.45	14.36(9.52 to 20.7)	4.09	10.27	97 (97)
United States of America	0.86	50.95(35.36 to 71.75)	4.07	46.88	204 (204)
United States Virgin Islands	0.82	8.76(5.74 to 12.88)	4.08	4.68	37 (29)

Uruguay	0.72	27.64(18.33 to 39.9)	4.06	23.58	193 (193)
Uzbekistan	0.66	14.19(9.45 to 20.99)	4.10	10.09	94 (94)
Vanuatu	0.47	22.53(15 to 32.64)	4.05	18.48	171 (171)
Venezuela (Bolivarian Republic of)	0.60	5.53(3.62 to 8.09)	4.03	1.50	8 (5)
Viet Nam	0.63	18.21(11.93 to 26.47)	4.11	14.09	132 (132)
Yemen	0.45	12.69(8.36 to 18.53)	4.06	8.63	63 (55)
Zambia	0.51	14.17(9.43 to 20.94)	4.07	10.10	95 (92)
Zimbabwe	0.47	13.85(9.12 to 20.45)	4.06	9.79	88 (87)

Table S11 Projected Analysis of Gender-Specific ASPR, ASIR, and ASDR for Gout Through 2045

	ASPR			ASIR			ASDR		
year	Both	Female	Male	Both	Female	Male	Both	Female	Male
1992	530.50	257.98	832.33	92.56	46.75	142.25	16.50	7.94	25.89
1993	528.66	257.01	828.53	92.39	46.61	141.97	16.45	7.91	25.79
1994	527.90	256.42	826.79	92.31	46.53	141.84	16.43	7.90	25.75
1995	528.46	256.31	827.55	92.34	46.50	141.87	16.45	7.89	25.78
1996	530.08	256.52	830.36	92.45	46.49	142.07	16.50	7.90	25.87
1997	532.45	256.82	834.66	92.62	46.49	142.41	16.57	7.90	26.00
1998	535.29	257.24	839.83	92.85	46.51	142.85	16.66	7.92	26.16
1999	538.34	257.78	845.28	93.13	46.56	143.34	16.75	7.93	26.33
2000	541.36	258.49	850.52	93.43	46.65	143.83	16.84	7.95	26.49
2001	545.77	260.37	857.44	94.02	46.98	144.66	16.98	8.01	26.71
2002	552.64	263.95	867.64	95.04	47.63	146.03	17.19	8.11	27.03
2003	560.80	268.47	879.56	96.26	48.43	147.66	17.45	8.25	27.40
2004	569.14	273.17	891.70	97.48	49.23	149.29	17.70	8.39	27.77
2005	576.58	277.28	902.65	98.47	49.87	150.63	17.93	8.51	28.11
2006	584.83	281.62	915.06	99.43	50.45	151.97	18.18	8.64	28.49
2007	595.32	286.94	931.12	100.59	51.10	153.62	18.50	8.80	28.98
2008	606.11	292.25	947.86	101.77	51.74	155.35	18.83	8.96	29.51
2009	615.28	296.60	962.23	102.81	52.28	156.90	19.11	9.09	29.94
2010	621.08	299.06	971.63	103.54	52.62	158.04	19.28	9.17	30.21
2011	624.62	299.96	977.99	104.14	52.81	159.07	19.39	9.19	30.42
2012	628.53	300.58	985.31	104.84	52.98	160.34	19.51	9.21	30.65
2013	632.49	301.12	992.82	105.57	53.13	161.66	19.64	9.23	30.89

2014	636.17	301.76	999.69	106.23	53.28	162.86	19.76	9.25	31.10
2015	639.37	302.73	1005.23	106.74	53.45	163.72	19.85	9.28	31.27
2016	643.40	304.94	1011.15	107.30	53.77	164.50	19.96	9.34	31.43
2017	648.98	308.54	1018.72	108.00	54.27	165.38	20.13	9.44	31.66
2018	654.21	312.22	1025.51	108.65	54.77	166.16	20.28	9.55	31.86
2019	657.23	314.59	1029.02	109.05	55.08	166.62	20.36	9.61	31.95
2020	657.46	315.37	1028.77	109.03	55.15	166.55	20.35	9.63	31.92
2021	653.82	315.05	1021.84	109.07	55.58	166.22	20.22	9.61	31.68
2022	660.96	319.39	1038.14	109.85	55.91	168.06	20.45	9.74	32.19
2023	664.21	321.44	1042.56	110.32	56.22	168.68	20.54	9.80	32.31
2024	667.57	323.48	1046.98	110.81	56.52	169.30	20.64	9.86	32.44
2025	669.21	324.74	1048.65	111.10	56.73	169.61	20.68	9.89	32.48
2026	670.85	326.01	1050.32	111.38	56.93	169.91	20.72	9.92	32.52
2027	672.51	327.27	1051.98	111.67	57.13	170.22	20.77	9.96	32.56
2028	674.19	328.53	1053.65	111.96	57.33	170.53	20.81	9.99	32.60
2029	675.86	329.79	1055.32	112.25	57.54	170.84	20.86	10.03	32.65
2030	675.96	330.29	1054.54	112.34	57.64	170.86	20.85	10.04	32.62
2031	676.06	330.78	1053.76	112.44	57.74	170.89	20.85	10.05	32.58
2032	676.17	331.28	1052.98	112.54	57.84	170.92	20.85	10.06	32.55
2033	676.31	331.77	1052.21	112.64	57.94	170.95	20.85	10.07	32.52
2034	676.43	332.27	1051.43	112.74	58.03	170.97	20.85	10.09	32.49
2035	675.17	332.03	1048.52	112.67	58.03	170.75	20.81	10.08	32.40
2036	673.90	331.79	1045.62	112.60	58.03	170.53	20.77	10.07	32.31
2037	672.64	331.55	1042.72	112.52	58.03	170.30	20.72	10.06	32.21
2038	671.41	331.31	1039.82	112.45	58.03	170.08	20.68	10.05	32.12
2039	670.16	331.07	1036.91	112.38	58.03	169.85	20.64	10.04	32.03
2040	668.66	330.65	1033.63	112.27	58.00	169.57	20.60	10.03	31.93
2041	667.15	330.24	1030.35	112.16	57.98	169.29	20.55	10.02	31.82
2042	665.65	329.83	1027.07	112.05	57.96	169.01	20.50	10.01	31.72
2043	664.16	329.41	1023.79	111.94	57.93	168.73	20.45	9.99	31.62
2044	662.66	329.00	1020.50	111.83	57.91	168.45	20.41	9.98	31.51
2045	661.15	328.58	1017.22	111.72	57.88	168.17	20.36	9.97	31.41

Table S12 Projected Analysis of Age-Specific ASPR for Female Gout Through 2045

	female-ASPR																
**year**	**15 to 19**	**20 to 24**	**25 to 29**	**30 to 34**	**35 to 39**	**40 to 44**	**45 to 49**	**50 to 54**	**55 to 59**	**60 to 64**	**65 to 69**	**70 to 74**	**75 to 79**	**80 to 84**	**85 to 89**	**90 to 94**	**95 plus**
1992	2.3548	16.9388	49.6249	93.0574	154.5638	227.0535	325.5266	442.3448	597.5426	781.5927	1033.4059	1343.2100	1609.0961	1837.5437	2030.2230	2249.9786	2517.0389
1993	2.3508	16.8584	49.3817	92.6360	152.7179	227.0661	324.3612	443.2112	596.1839	780.3260	1028.0644	1333.5813	1604.0729	1830.1564	2021.5903	2231.8648	2495.1474
1994	2.3477	16.7980	49.1868	92.8200	150.9662	227.0788	324.9626	444.5581	594.5787	780.0666	1023.3852	1327.1525	1597.0069	1828.2670	2017.7008	2219.6513	2476.7564
1995	2.3458	16.7578	49.0892	93.4466	149.8958	228.2241	325.0622	446.4332	593.8581	779.7108	1020.5399	1321.3608	1596.2405	1829.8506	2022.1055	2215.9510	2473.5863
1996	2.3458	16.7319	49.0407	94.2622	149.6343	228.9165	326.2884	448.7306	593.8513	779.5831	1018.6884	1316.5708	1596.3287	1835.8938	2029.5279	2217.8673	2475.4821
1997	2.3479	16.7132	49.0299	94.8787	150.1077	229.0930	327.8934	450.3132	595.1767	779.1913	1018.1277	1312.1752	1596.3834	1841.0713	2038.0043	2222.8727	2475.2668
1998	2.3517	16.7003	49.0458	95.2244	151.3589	228.5072	330.1665	451.0004	597.9177	779.1466	1018.2980	1310.0981	1595.6200	1846.3692	2045.0845	2230.3321	2474.6336
1999	2.3568	16.6934	49.0495	95.3751	152.9201	227.5481	331.7176	453.1964	601.3588	779.5255	1020.1154	1308.9369	1596.7692	1848.0521	2053.7987	2237.2894	2471.3882
2000	2.3622	16.6883	49.0055	95.3937	154.4109	226.7382	333.8047	454.4085	605.0664	781.3369	1022.6853	1310.1321	1597.1144	1855.3320	2061.4939	2245.9258	2467.2800
2001	2.3712	16.7036	48.9591	95.3658	155.8478	226.6604	335.2880	457.3441	610.8264	787.9759	1031.8010	1320.5196	1607.1069	1872.7198	2083.4465	2270.3358	2490.3122
2002	2.3854	16.7552	48.9535	95.4894	157.0750	227.7861	336.7434	461.7922	619.6188	801.6104	1049.4465	1344.4107	1630.7234	1905.7278	2124.8361	2320.1563	2554.8436
2003	2.4019	16.8351	48.9867	95.7170	157.9909	230.1633	337.5476	467.7962	628.8664	820.0946	1071.6774	1375.2505	1663.8999	1945.2284	2178.9094	2380.9055	2635.5789
2004	2.4177	16.9371	49.0596	95.9564	158.6772	233.1424	337.8335	473.2087	639.9758	839.7853	1093.7912	1408.7656	1697.6569	1987.0595	2230.9325	2444.3360	2706.0936
2005	2.4305	17.0494	49.1564	96.1207	159.1916	236.1277	338.0491	478.7528	648.6872	857.0632	1113.4834	1437.3002	1728.0351	2021.2137	2280.5379	2497.8255	2751.8697
2006	2.4409	17.1825	49.3224	96.3162	159.6810	239.1358	339.1256	483.0879	658.1801	874.3613	1135.2956	1466.9516	1762.9279	2059.8405	2330.5053	2553.2035	2778.5908
2007	2.4510	17.3382	49.5898	96.5896	160.4568	241.8763	341.8784	487.2079	668.6500	895.1446	1164.2077	1501.8791	1808.6322	2108.3682	2391.0588	2618.3670	2807.2458
2008	2.4587	17.4924	49.9233	96.8681	161.3379	244.0433	346.3003	490.1062	680.1376	913.0111	1197.0357	1535.8283	1853.5751	2158.4201	2445.2833	2682.3281	2832.5233
2009	2.4623	17.6188	50.3017	97.1059	162.0664	245.6531	351.4265	491.7615	689.1895	929.5096	1225.9618	1561.7325	1890.1613	2193.1759	2486.4521	2722.3649	2852.2491
2010	2.4606	17.6901	50.6712	97.2258	162.3870	246.7017	356.2793	492.7119	696.2830	937.5677	1243.6371	1575.6207	1906.8389	2207.6784	2501.2004	2739.6881	2866.2291
2011	2.4582	17.7159	51.0661	97.3111	162.3891	247.3335	360.8224	494.2907	700.1426	941.1027	1250.0780	1579.6154	1906.1374	2204.7476	2494.4575	2735.0338	2876.0759

2012	2.4584	17.7264	51.5015	97.5172	162.2987	248.1483	364.7732	497.9146	702.6115	942.3511	1255.8772	1583.4880	1898.3954	2198.0706	2480.9815	2729.3749	2885.0905
2013	2.4601	17.7179	51.8951	97.8630	162.2087	249.0831	367.8333	503.8341	703.1144	945.0329	1255.3418	1591.2335	1889.6545	2189.8906	2470.9418	2720.8123	2897.0233
2014	2.4616	17.6885	52.1724	98.3773	162.2051	249.9051	370.2393	510.8954	702.3858	947.1187	1256.6350	1599.6715	1883.5527	2184.9162	2459.7042	2717.0894	2902.9080
2015	2.4613	17.6394	52.2836	99.0287	162.3028	250.3977	372.1602	517.8271	701.9226	950.7940	1259.6845	1609.7324	1885.6467	2184.4522	2456.0305	2715.1747	2911.3301
2016	2.4592	17.5896	52.2415	99.8482	162.6393	250.8578	374.1761	525.2652	704.6143	957.6751	1270.1075	1626.9765	1904.7263	2198.0523	2467.2855	2723.9486	2923.6908
2017	2.4560	17.5474	52.1144	100.7378	163.2819	251.4247	376.8339	532.6007	711.6324	967.4387	1284.5439	1660.7005	1941.3666	2224.3063	2493.8955	2745.2850	2950.1818
2018	2.4509	17.5043	51.9188	101.4962	164.1223	251.8614	379.3113	538.0932	721.4697	974.9488	1302.1912	1688.3544	1987.6874	2252.3279	2520.3223	2769.9541	2974.3300
2019	2.4430	17.4532	51.6615	101.9610	165.0790	251.9727	380.5561	540.8932	730.7164	976.6643	1313.9752	1707.3858	2023.1432	2268.0489	2532.9022	2774.7101	2990.4937
2020	2.4267	17.3652	51.3802	102.2065	166.2731	252.1474	380.6604	541.4141	736.3493	972.4523	1317.8110	1715.3922	2040.1914	2270.5890	2528.7525	2771.4197	3001.6929
2021	2.4060	17.2401	50.9664	101.7764	167.3891	252.2570	380.3831	542.4137	743.1112	971.4893	1318.6836	1709.4551	2033.0699	2262.4184	2502.2684	2730.3420	2947.0905
2022	2.5852	17.4638	51.5076	101.1392	167.3656	253.2481	378.5078	541.9788	747.2129	993.6735	1329.3716	1734.8910	2072.9282	2347.6924	2595.8126	2839.6000	3048.6355
2023	2.6348	17.4777	51.4726	100.9714	168.0641	253.6856	378.1569	542.9233	753.2097	1000.6936	1336.4867	1747.3440	2095.3388	2378.2442	2622.4944	2866.7298	3073.8363
2024	2.6844	17.4917	51.4375	100.8036	168.7626	254.1232	377.8059	543.8678	759.2065	1007.7136	1343.6019	1759.7970	2117.7494	2408.7959	2649.1763	2893.8596	3099.0372
2025	2.7242	17.6683	51.3137	100.5105	168.2945	254.7656	378.0335	543.1981	760.1357	1014.4591	1350.6151	1765.7691	2129.9760	2430.5269	2675.7081	2912.9440	3116.8987
2026	2.7640	17.8450	51.1899	100.2175	167.8265	255.4080	378.2611	542.5284	761.0649	1021.2046	1357.6282	1771.7413	2142.2026	2452.2578	2702.2399	2932.0284	3134.7602
2027	2.8038	18.0217	51.0661	99.9244	167.3584	256.0503	378.4887	541.8586	761.9942	1027.9501	1364.6414	1777.7135	2154.4293	2473.9888	2728.7717	2951.1128	3152.6216
2028	2.8436	18.1983	50.9424	99.6314	166.8904	256.6927	378.7162	541.1889	762.9234	1034.6957	1371.6546	1783.6857	2166.6559	2495.7198	2755.3035	2970.1972	3170.4831
2029	2.8834	18.3750	50.8186	99.3383	166.4224	257.3351	378.9438	540.5192	763.8526	1041.4412	1378.6677	1789.6579	2178.8825	2517.4507	2781.8353	2989.2816	3188.3446
2030	2.9112	18.4967	51.0925	98.9000	165.6396	256.1897	379.1478	539.9249	761.7982	1041.1170	1385.3865	1796.0931	2183.1971	2528.3492	2802.7675	3014.8340	3205.1599
2031	2.9390	18.6184	51.3664	98.4617	164.8568	255.0444	379.3518	539.3307	759.7437	1040.7929	1392.1053	1802.5283	2187.5117	2539.2477	2823.6998	3040.3864	3221.9752
2032	2.9668	18.7400	51.6404	98.0233	164.0739	253.8990	379.5558	538.7365	757.6893	1040.4688	1398.8241	1808.9635	2191.8263	2550.1462	2844.6320	3065.9387	3238.7905
2033	2.9947	18.8617	51.9143	97.5850	163.2911	252.7537	379.7598	538.1422	755.6348	1040.1447	1405.5428	1815.3987	2196.1408	2561.0446	2865.5643	3091.4911	3255.6058
2034	3.0225	18.9834	52.1882	97.1467	162.5083	251.6083	379.9638	537.5480	753.5804	1039.8206	1412.2616	1821.8339	2200.4554	2571.9431	2886.4965	3117.0435	3272.4211
2035	3.0368	19.0455	52.3274	97.3753	161.5215	250.0410	377.7344	536.9247	751.6417	1035.6940	1409.9188	1827.8147	2205.2526	2573.7243	2895.1719	3136.2158	3295.9724
2036	3.0511	19.1075	52.4665	97.6039	160.5346	248.4737	375.5050	536.3014	749.7030	1031.5674	1407.5760	1833.7956	2210.0497	2575.5054	2903.8473	3155.3880	3319.5236

2037	3.0654	19.1695	52.6057	97.8326	159.5477	246.9064	373.2756	535.6781	747.7643	1027.4409	1405.2332	1839.7765	2214.8468	2577.2865	2912.5227	3174.5602	3343.0748
2038	3.0797	19.2316	52.7449	98.0612	158.5608	245.3390	371.0463	535.0547	745.8256	1023.3143	1402.8904	1845.7573	2219.6439	2579.0676	2921.1981	3193.7324	3366.6260
2039	3.0940	19.2936	52.8840	98.2898	157.5739	243.7717	368.8169	534.4314	743.8869	1019.1877	1400.5476	1851.7382	2224.4410	2580.8487	2929.8735	3212.9046	3390.1773
2040	3.1086	19.3565	53.0247	98.5206	157.9104	242.4053	366.6866	531.5002	743.0785	1016.7186	1395.3084	1848.8278	2231.4560	2586.2978	2931.8502	3222.3552	3410.6780
2041	3.1232	19.4193	53.1653	98.7514	158.2469	241.0390	364.5563	528.5690	742.2701	1014.2495	1390.0692	1845.9175	2238.4709	2591.7468	2933.8269	3231.8057	3431.1786
2042	3.1377	19.4822	53.3059	98.9822	158.5834	239.6726	362.4261	525.6378	741.4617	1011.7804	1384.8301	1843.0072	2245.4858	2597.1959	2935.8037	3241.2562	3451.6793
2043	3.1523	19.5450	53.4466	99.2129	158.9199	238.3062	360.2958	522.7066	740.6533	1009.3113	1379.5909	1840.0968	2252.5007	2602.6449	2937.7804	3250.7067	3472.1800
2044	3.1669	19.6079	53.5872	99.4437	159.2564	236.9399	358.1656	519.7754	739.8448	1006.8422	1374.3517	1837.1865	2259.5156	2608.0940	2939.7571	3260.1572	3492.6807
2045	3.1815	19.6707	53.7279	99.6745	159.5930	235.5735	356.0353	516.8442	739.0364	1004.3731	1369.1125	1834.2761	2266.5306	2613.5430	2941.7338	3269.6077	3513.1813

Table S13 Projected Analysis of Age-Specific ASIR for Female Gout Through 2045

	female-ASIR																
**year**	**15 to 19**	**20 to 24**	**25 to 29**	**30 to 34**	**35 to 39**	**40 to 44**	**45 to 49**	**50 to 54**	**55 to 59**	**60 to 64**	**65 to 69**	**70 to 74**	**75 to 79**	**80 to 84**	**85 to 89**	**90 to 94**	**95 plus**
1992	1.6466	7.1553	14.9324	23.5329	34.4916	48.6271	67.1654	88.9277	116.0206	146.6581	183.0805	214.1852	235.7233	249.2197	259.3744	284.4881	324.7434
1993	1.6439	7.1107	14.8587	23.4810	34.1812	48.7906	67.1594	89.1858	115.8016	146.4074	182.1243	212.4811	235.2832	247.9817	257.7972	282.0198	321.1272
1994	1.6418	7.0772	14.7985	23.5633	33.8598	48.8947	67.4044	89.4857	115.5097	146.2810	181.2857	211.3311	234.4983	247.4753	256.8192	280.3213	318.4055
1995	1.6405	7.0556	14.7671	23.7352	33.6447	49.1498	67.4521	89.7874	115.3311	146.1066	180.7105	210.4498	234.0686	247.7853	256.6785	279.6096	317.3813
1996	1.6406	7.0414	14.7498	23.9372	33.5729	49.2632	67.6446	90.0936	115.2400	145.9226	180.2012	209.6850	233.3321	248.6862	257.1054	279.3153	317.4284
1997	1.6420	7.0292	14.7442	24.0812	33.6498	49.2624	67.8947	90.3050	115.3689	145.6370	179.7876	208.8538	232.3178	249.4995	257.4967	279.6007	317.1914
1998	1.6447	7.0197	14.7473	24.1536	33.8926	49.1128	68.2706	90.3745	115.7312	145.3899	179.4589	208.2450	231.2681	250.3586	257.9573	280.1064	317.2953
1999	1.6482	7.0139	14.7468	24.1787	34.2070	48.9011	68.5305	90.7189	116.2319	145.2325	179.4079	207.8340	230.7430	250.7572	258.9137	280.8803	317.3806
2000	1.6519	7.0105	14.7313	24.1770	34.5187	48.7395	68.9062	90.9389	116.7930	145.3859	179.5918	207.8794	230.6998	251.2751	260.6292	282.1410	317.7565
2001	1.6582	7.0155	14.7129	24.1693	34.8324	48.7493	69.2188	91.6060	117.9511	146.5876	181.1115	209.4240	232.2981	252.9167	264.8360	286.7071	322.6934

2002	1.6681	7.0344	14.7041	24.2006	35.1079	49.0132	69.5718	92.7118	120.0378	149.2642	184.2356	212.9889	235.7383	256.5550	271.8653	295.1290	334.1486
2003	1.6796	7.0653	14.7056	24.2590	35.3176	49.5280	69.8244	94.1942	122.3841	152.8708	188.1406	217.5417	240.2357	261.0719	280.0974	304.7109	347.3250
2004	1.6907	7.1074	14.7189	24.3200	35.4737	50.1548	69.9711	95.5786	125.0492	156.6840	191.9016	222.2995	244.6124	265.7625	286.7856	313.0715	357.6276
2005	1.6996	7.1573	14.7403	24.3605	35.5827	50.7614	70.0685	96.8940	127.0763	159.8875	195.0333	226.0937	248.1556	269.2995	290.6604	317.9175	361.9235
2006	1.7070	7.2211	14.7873	24.4037	35.6753	51.3519	70.2976	97.9215	129.0516	162.8494	198.1348	229.5684	251.4162	272.1183	291.6543	320.0519	360.2439
2007	1.7141	7.2983	14.8707	24.4630	35.8261	51.8870	70.8314	98.8814	131.1263	166.2520	202.1483	233.3249	255.3229	274.9268	292.1379	321.7045	356.3392
2008	1.7197	7.3754	14.9780	24.5252	36.0027	52.3090	71.6651	99.5489	133.3433	169.1043	206.5987	236.7850	259.0819	277.7030	292.2247	322.9695	351.6596
2009	1.7224	7.4377	15.1008	24.5829	36.1540	52.6290	72.6211	99.8902	134.9912	171.8576	210.5424	239.2889	262.2194	279.7843	292.7585	322.7664	348.1580
2010	1.7213	7.4711	15.2205	24.6199	36.2298	52.8524	73.5157	99.9970	136.1500	173.2038	213.0007	240.7176	263.8513	281.3102	293.8284	322.2610	347.9717
2011	1.7197	7.4795	15.3466	24.6524	36.2502	53.0154	74.3665	100.1517	136.5477	173.7346	213.9050	241.1104	264.2906	282.3535	296.2537	323.3127	352.4045
2012	1.7199	7.4779	15.4844	24.7146	36.2583	53.2278	75.1434	100.6556	136.5984	173.6399	214.3896	241.5086	264.1577	283.5172	299.6517	327.5397	359.9048
2013	1.7211	7.4668	15.6079	24.8121	36.2691	53.4710	75.7722	101.5545	136.2629	173.6867	213.9393	242.2362	263.8443	284.6752	303.4440	332.7171	368.4192
2014	1.7222	7.4470	15.6931	24.9535	36.2962	53.6909	76.2807	102.6373	135.7381	173.6245	214.0303	243.1362	263.5788	285.8510	306.0839	337.4730	374.5507
2015	1.7221	7.4211	15.7253	25.1324	36.3391	53.8374	76.6808	103.6796	135.3336	174.0938	214.4018	244.3262	263.9991	286.8572	307.3231	339.7745	377.2918
2016	1.7207	7.3992	15.7129	25.3651	36.4516	53.9846	77.0941	104.8216	135.4918	175.2079	216.3333	247.0469	266.2620	288.4884	306.9913	337.9531	374.0824
2017	1.7185	7.3852	15.6798	25.6286	36.6561	54.1669	77.6389	106.0127	136.3876	176.7443	219.1927	251.9840	270.8124	290.6659	306.2648	333.4020	368.0916
2018	1.7149	7.3741	15.6283	25.8621	36.9020	54.3183	78.1642	106.9489	137.8508	177.8851	222.5508	256.1595	276.0816	292.6101	305.2783	329.0176	362.2937
2019	1.7093	7.3614	15.5569	26.0136	37.1454	54.3854	78.4723	107.4771	139.3585	178.0967	224.6459	258.9977	279.6032	293.3222	304.0446	326.2771	360.2256
2020	1.6979	7.3400	15.4960	26.1128	37.4517	54.5005	78.7109	107.9423	140.8650	177.8551	225.3909	258.1285	276.9707	290.0860	304.0347	330.0634	367.8528
2021	1.6832	7.2981	15.3829	26.0523	37.7878	54.6341	78.7052	108.2156	142.2810	178.5284	227.5522	261.7082	282.0377	294.9019	308.4441	333.6871	370.1017
2022	1.7644	7.3676	15.5388	25.8317	37.7720	54.7903	78.3003	108.3706	143.1759	181.1714	226.7472	263.3118	285.7322	301.6383	313.7083	339.9148	373.5736
2023	1.7844	7.3729	15.5351	25.7968	37.9627	54.9195	78.2865	108.7162	144.4158	182.2849	227.6626	265.2101	288.5881	304.7365	316.4009	343.0566	376.1606
2024	1.8043	7.3782	15.5314	25.7620	38.1535	55.0487	78.2727	109.0618	145.6556	183.3984	228.5780	267.1084	291.4440	307.8346	319.0935	346.1984	378.7477
2025	1.8199	7.4260	15.5041	25.7095	38.0766	55.2637	78.4081	109.0419	145.9961	184.6598	229.5949	267.8735	293.2551	310.5045	321.7470	348.0439	380.5949
2026	1.8354	7.4738	15.4767	25.6570	37.9997	55.4786	78.5435	109.0220	146.3366	185.9213	230.6119	268.6386	295.0661	313.1745	324.4004	349.8894	382.4421

2027	1.8510	7.5216	15.4493	25.6045	37.9228	55.6935	78.6789	109.0021	146.6771	187.1827	231.6288	269.4037	296.8772	315.8445	327.0539	351.7349	384.2894
2028	1.8665	7.5694	15.4219	25.5520	37.8459	55.9085	78.8143	108.9822	147.0176	188.4442	232.6457	270.1687	298.6883	318.5144	329.7074	353.5804	386.1366
2029	1.8821	7.6172	15.3946	25.4995	37.7689	56.1234	78.9498	108.9623	147.3581	189.7056	233.6626	270.9338	300.4993	321.1844	332.3609	355.4260	387.9839
2030	1.8928	7.6497	15.4516	25.4168	37.6402	55.9397	79.1304	109.0026	147.1607	189.9135	234.9373	271.8133	301.0319	322.8123	334.8575	357.9578	389.6235
2031	1.9034	7.6823	15.5087	25.3341	37.5114	55.7560	79.3110	109.0429	146.9633	190.1214	236.2119	272.6929	301.5644	324.4402	337.3542	360.4896	391.2632
2032	1.9141	7.7148	15.5658	25.2514	37.3826	55.5724	79.4917	109.0832	146.7659	190.3293	237.4865	273.5724	302.0970	326.0681	339.8508	363.0214	392.9028
2033	1.9247	7.7474	15.6229	25.1686	37.2539	55.3887	79.6723	109.1235	146.5685	190.5371	238.7611	274.4519	302.6295	327.6960	342.3475	365.5532	394.5425
2034	1.9354	7.7799	15.6800	25.0859	37.1251	55.2050	79.8529	109.1637	146.3710	190.7450	240.0357	275.3314	303.1620	329.3239	344.8441	368.0850	396.1821
2035	1.9408	7.7964	15.7088	25.1279	36.9569	54.9530	79.5051	109.2612	146.2505	190.2917	240.0311	276.4944	303.8135	329.5668	346.2201	370.4244	398.5522
2036	1.9463	7.8129	15.7377	25.1699	36.7887	54.7010	79.1573	109.3586	146.1300	189.8383	240.0265	277.6573	304.4650	329.8097	347.5961	372.7639	400.9223
2037	1.9517	7.8294	15.7665	25.2120	36.6205	54.4489	78.8095	109.4560	146.0095	189.3849	240.0218	278.8202	305.1164	330.0526	348.9721	375.1034	403.2923
2038	1.9571	7.8458	15.7954	25.2540	36.4523	54.1969	78.4617	109.5534	145.8890	188.9316	240.0172	279.9831	305.7679	330.2956	350.3481	377.4428	405.6624
2039	1.9625	7.8623	15.8242	25.2960	36.2841	53.9449	78.1139	109.6509	145.7685	188.4782	240.0125	281.1460	306.4193	330.5385	351.7241	379.7823	408.0324
2040	1.9680	7.8789	15.8533	25.3383	36.3405	53.7179	77.7810	109.2024	145.8913	188.3307	239.4676	281.1408	307.6859	331.2365	351.9802	381.2685	410.5722
2041	1.9735	7.8956	15.8824	25.3806	36.3970	53.4910	77.4481	108.7539	146.0141	188.1831	238.9226	281.1355	308.9525	331.9346	352.2362	382.7548	413.1121
2042	1.9790	7.9122	15.9115	25.4229	36.4534	53.2640	77.1152	108.3054	146.1368	188.0356	238.3777	281.1302	310.2192	332.6327	352.4923	384.2410	415.6519
2043	1.9845	7.9288	15.9405	25.4651	36.5098	53.0371	76.7823	107.8569	146.2596	187.8881	237.8327	281.1250	311.4858	333.3307	352.7483	385.7272	418.1917
2044	1.9899	7.9454	15.9696	25.5074	36.5662	52.8101	76.4494	107.4084	146.3824	187.7405	237.2878	281.1197	312.7524	334.0288	353.0044	387.2134	420.7315
2045	1.9954	7.9620	15.9987	25.5497	36.6227	52.5832	76.1165	106.9599	146.5051	187.5930	236.7428	281.1144	314.0190	334.7268	353.2604	388.6997	423.2713

Table S14 Projected Analysis of Age-Specific ASDR for Female Gout Through 2045

year	female-ASDR																
	**15 to 19**	**20 to 24**	**25 to 29**	**30 to 34**	**35 to 39**	**40 to 44**	**45 to 49**	**50 to 54**	**55 to 59**	**60 to 64**	**65 to 69**	**70 to 74**	**75 to 79**	**80 to 84**	**85 to 89**	**90 to 94**	**95 plus**
1992	0.0826	0.5941	1.7420	3.2165	5.1288	7.4691	10.6339	14.3383	19.0299	24.3790	31.2479	39.8532	46.7078	52.2802	56.2144	60.6839	66.1953

1993	0.0825	0.5913	1.7334	3.2022	5.0816	7.4494	10.5942	14.3983	18.9476	24.3525	31.0307	39.5432	46.6167	52.0655	55.9606	60.1451	65.6224
1994	0.0823	0.5891	1.7265	3.1973	5.0134	7.4911	10.6040	14.4272	18.9450	24.3025	30.9293	39.3613	46.4336	52.0346	55.8736	59.8207	65.0979
1995	0.0823	0.5877	1.7230	3.2241	4.9689	7.4809	10.6001	14.5072	18.8819	24.2726	30.8484	39.2414	46.4408	52.0968	55.9769	59.7173	65.0280
1996	0.0823	0.5868	1.7213	3.2378	4.9680	7.5048	10.6531	14.5942	18.9002	24.3094	30.7931	39.0309	46.3985	52.2209	56.2040	59.6842	65.0514
1997	0.0824	0.5862	1.7210	3.2652	4.9812	7.5267	10.7084	14.6163	18.9368	24.2581	30.7611	38.8685	46.3382	52.3478	56.3647	59.8136	64.9307
1998	0.0825	0.5857	1.7211	3.2817	5.0310	7.5164	10.7890	14.6171	19.0179	24.2815	30.7597	38.8161	46.3477	52.4618	56.5655	60.0404	64.8391
1999	0.0827	0.5855	1.7217	3.2878	5.0847	7.4759	10.7909	14.6964	19.1079	24.2471	30.7882	38.7689	46.3494	52.4054	56.6933	60.1721	64.8104
2000	0.0829	0.5853	1.7199	3.2909	5.1268	7.4446	10.8840	14.7215	19.2204	24.3364	30.8793	38.7625	46.3776	52.5941	56.8947	60.3900	64.6104
2001	0.0832	0.5859	1.7178	3.2900	5.1662	7.4543	10.9376	14.8441	19.4135	24.5284	31.1387	39.1012	46.6515	53.0835	57.5417	60.9669	65.1468
2002	0.0837	0.5877	1.7177	3.3036	5.2199	7.4918	10.9739	15.0081	19.6572	24.9140	31.7275	39.7693	47.3211	54.0316	58.6003	62.3678	66.8286
2003	0.0843	0.5905	1.7190	3.3012	5.2652	7.5553	11.0245	15.1732	19.9739	25.5149	32.3856	40.7345	48.2527	55.1628	60.0838	63.9304	68.8563
2004	0.0848	0.5941	1.7221	3.3137	5.2715	7.6533	11.0528	15.3141	20.2935	26.1351	33.0128	41.7361	49.2365	56.3416	61.5325	65.6621	70.6065
2005	0.0853	0.5980	1.7250	3.3251	5.2969	7.7607	11.0167	15.5459	20.5445	26.6845	33.6206	42.5482	50.0925	57.3061	62.8472	66.9734	71.8077
2006	0.0856	0.6027	1.7306	3.3256	5.3168	7.8529	11.0777	15.6841	20.8909	27.1908	34.2646	43.4876	51.1092	58.3798	64.2248	68.4574	72.6413
2007	0.0860	0.6081	1.7400	3.3343	5.3340	7.9495	11.1500	15.8177	21.2077	27.8214	35.1144	44.4256	52.4238	59.7038	65.9493	70.2278	73.2581
2008	0.0862	0.6135	1.7519	3.3555	5.3718	8.0488	11.3023	15.8847	21.5834	28.3285	36.1672	45.4870	53.7649	61.1191	67.4047	71.9973	74.0075
2009	0.0864	0.6180	1.7654	3.3591	5.3947	8.0874	11.4717	15.9590	21.8606	28.8405	37.0189	46.2482	54.7745	62.1009	68.4704	73.0045	74.4469
2010	0.0863	0.6205	1.7781	3.3639	5.4072	8.1284	11.6417	15.9709	22.1288	29.1038	37.5239	46.6298	55.2356	62.4875	68.9001	73.4387	74.8545
2011	0.0862	0.6214	1.7927	3.3619	5.4087	8.1474	11.7846	16.0184	22.2411	29.2066	37.7362	46.7361	55.2005	62.3872	68.7197	73.2832	75.0583
2012	0.0862	0.6217	1.8080	3.3682	5.3956	8.1679	11.9326	16.1446	22.2766	29.2779	37.8866	46.8847	54.9630	62.2289	68.3431	73.1475	75.3070
2013	0.0863	0.6215	1.8218	3.3797	5.3908	8.2027	12.0412	16.3354	22.2749	29.3237	37.9131	47.1009	54.7415	61.9224	68.0385	72.9031	75.6861
2014	0.0863	0.6204	1.8315	3.3875	5.3904	8.2286	12.1295	16.5919	22.2782	29.3811	37.9372	47.3535	54.6053	61.7909	67.7136	72.7966	75.8725
2015	0.0863	0.6187	1.8353	3.4102	5.3921	8.2358	12.1761	16.7916	22.2937	29.4907	38.0332	47.5927	54.5444	61.7900	67.6279	72.7962	76.0371
2016	0.0863	0.6169	1.8338	3.4396	5.4127	8.2641	12.2245	17.0000	22.3356	29.5753	38.2816	48.0894	55.1171	62.1779	67.8868	73.0268	76.4505
2017	0.0861	0.6154	1.8294	3.4715	5.4228	8.2711	12.3287	17.2248	22.5931	29.8822	38.7325	49.0341	56.1165	62.8485	68.6129	73.5888	77.0197

2018	0.0860	0.6139	1.8225	3.4943	5.4450	8.2809	12.3997	17.3837	22.8474	30.1419	39.2477	49.8387	57.4419	63.6065	69.2775	74.2489	77.5626
2019	0.0857	0.6121	1.8135	3.5141	5.4719	8.2920	12.4361	17.3950	23.1235	30.2156	39.5989	50.4194	58.3913	63.9454	69.5871	74.2934	77.9896
2020	0.0851	0.6090	1.8035	3.5175	5.5128	8.2833	12.4345	17.4126	23.2868	30.0429	39.6794	50.6396	58.8708	63.9949	69.4399	74.1232	78.2032
2021	0.0844	0.6047	1.7892	3.5057	5.5346	8.2901	12.4132	17.3889	23.4356	29.9268	39.6768	50.3480	58.5720	63.6746	68.6320	72.9096	76.6662
2022	0.0901	0.6115	1.8041	3.4800	5.5429	8.3042	12.3426	17.4738	23.6018	30.6697	39.9587	51.1406	59.7781	66.1641	71.2107	75.8854	79.3621
2023	0.0916	0.6117	1.8016	3.4730	5.5643	8.3111	12.3225	17.5111	23.7781	30.8790	40.1438	51.4905	60.4059	67.0105	71.9106	76.5725	79.9865
2024	0.0932	0.6118	1.7990	3.4660	5.5858	8.3180	12.3025	17.5483	23.9545	31.0883	40.3289	51.8404	61.0338	67.8569	72.6105	77.2596	80.6110
2025	0.0944	0.6173	1.7946	3.4552	5.5698	8.3382	12.3035	17.5205	23.9847	31.2869	40.5306	51.9901	61.3740	68.4466	73.3247	77.7423	81.0492
2026	0.0956	0.6228	1.7902	3.4443	5.5538	8.3584	12.3045	17.4927	24.0149	31.4855	40.7322	52.1399	61.7142	69.0364	74.0388	78.2250	81.4875
2027	0.0968	0.6283	1.7858	3.4335	5.5378	8.3787	12.3055	17.4648	24.0451	31.6841	40.9339	52.2896	62.0544	69.6261	74.7530	78.7077	81.9257
2028	0.0981	0.6338	1.7814	3.4226	5.5218	8.3989	12.3065	17.4370	24.0753	31.8827	41.1355	52.4393	62.3946	70.2159	75.4671	79.1905	82.3639
2029	0.0993	0.6393	1.7770	3.4118	5.5058	8.4191	12.3075	17.4092	24.1055	32.0814	41.3371	52.5891	62.7348	70.8056	76.1813	79.6732	82.8022
2030	0.1001	0.6430	1.7856	3.3974	5.4798	8.3825	12.3152	17.3844	24.0359	32.0767	41.5330	52.7739	62.8355	71.1067	76.7400	80.3505	83.2192
2031	0.1010	0.6468	1.7941	3.3831	5.4538	8.3458	12.3229	17.3596	23.9664	32.0721	41.7289	52.9587	62.9363	71.4077	77.2988	81.0279	83.6362
2032	0.1019	0.6506	1.8026	3.3687	5.4278	8.3092	12.3307	17.3349	23.8968	32.0675	41.9248	53.1435	63.0371	71.7088	77.8576	81.7053	84.0531
2033	0.1027	0.6543	1.8111	3.3544	5.4018	8.2725	12.3384	17.3101	23.8273	32.0629	42.1207	53.3283	63.1378	72.0098	78.4164	82.3827	84.4701
2034	0.1036	0.6581	1.8196	3.3400	5.3759	8.2359	12.3461	17.2853	23.7578	32.0582	42.3166	53.5131	63.2386	72.3109	78.9751	83.0601	84.8871
2035	0.1040	0.6600	1.8239	3.3470	5.3452	8.1859	12.2768	17.2695	23.6927	31.9288	42.2578	53.6886	63.3785	72.3417	79.2146	83.5649	85.5037
2036	0.1044	0.6619	1.8282	3.3541	5.3145	8.1359	12.2075	17.2536	23.6277	31.7993	42.1989	53.8640	63.5183	72.3725	79.4541	84.0698	86.1203
2037	0.1049	0.6638	1.8326	3.3611	5.2838	8.0860	12.1382	17.2377	23.5626	31.6699	42.1400	54.0395	63.6582	72.4033	79.6936	84.5747	86.7370
2038	0.1053	0.6658	1.8369	3.3681	5.2531	8.0360	12.0689	17.2218	23.4975	31.5404	42.0812	54.2149	63.7980	72.4341	79.9330	85.0795	87.3536
2039	0.1057	0.6677	1.8412	3.3751	5.2224	7.9860	11.9995	17.2060	23.4325	31.4110	42.0223	54.3904	63.9379	72.4649	80.1725	85.5844	87.9702
2040	0.1062	0.6696	1.8456	3.3822	5.2324	7.9439	11.9320	17.1155	23.4122	31.3296	41.8615	54.3184	64.1402	72.6208	80.2060	85.8398	88.4987
2041	0.1066	0.6716	1.8499	3.3892	5.2425	7.9017	11.8644	17.0251	23.3920	31.2482	41.7007	54.2465	64.3424	72.7768	80.2394	86.0951	89.0273
2042	0.1071	0.6735	1.8543	3.3963	5.2525	7.8596	11.7968	16.9346	23.3717	31.1669	41.5398	54.1745	64.5447	72.9327	80.2729	86.3505	89.5558

2043	0.1075	0.6754	1.8586	3.4034	5.2625	7.8174	11.7292	16.8441	23.3515	31.0855	41.3790	54.1026	64.7470	73.0886	80.3063	86.6058	90.0843
2044	0.1080	0.6774	1.8630	3.4105	5.2725	7.7753	11.6616	16.7537	23.3312	31.0041	41.2182	54.0306	64.9492	73.2446	80.3398	86.8612	90.6129
2045	0.1084	0.6793	1.8673	3.4175	5.2826	7.7331	11.5941	16.6632	23.3110	30.9228	41.0574	53.9586	65.1515	73.4005	80.3732	87.1165	91.1414

Table S15 Projected Analysis of Age-Specific ASPR for Male Gout Through 2045

year	male-ASPR																
	15 to 19	20 to 24	25 to 29	30 to 34	35 to 39	40 to 44	45 to 49	50 to 54	55 to 59	60 to 64	65 to 69	70 to 74	75 to 79	80 to 84	85 to 89	90 to 94	95 plus
1992	4.7342	47.8747	182.3330	379.0527	656.2363	979.6946	1359.3598	1751.9153	2165.3112	2585.3232	3096.8359	3627.0381	4049.2633	4372.7590	4587.0115	4887.8227	5378.0479
1993	4.7286	47.8127	182.8395	381.7504	655.0824	980.4984	1363.4880	1759.7314	2159.8410	2570.9396	3067.6521	3587.8840	3993.0958	4322.8000	4526.7426	4803.8118	5279.8549
1994	4.7247	47.6910	183.0459	385.4186	653.2052	981.6629	1370.1645	1770.2685	2157.3486	2564.5303	3047.7154	3565.0323	3952.8171	4292.9234	4492.2979	4743.9480	5215.5692
1995	4.7198	47.4773	183.0519	388.7303	651.9628	985.6165	1377.5049	1780.2760	2160.0982	2564.8185	3040.8257	3557.3167	3944.8600	4286.5454	4491.7231	4717.8253	5198.0884
1996	4.7176	47.2619	183.3235	391.9357	652.7431	991.4137	1384.9554	1790.6121	2169.3067	2572.1374	3042.9402	3559.6719	3957.4880	4296.4605	4513.6021	4720.7894	5207.6275
1997	4.7219	47.1548	183.8952	395.4555	656.7063	997.7864	1390.3482	1812.2438	2184.5980	2581.9324	3049.5956	3564.5780	3975.6896	4308.4937	4541.9839	4740.8990	5219.6180
1998	4.7309	47.1310	184.5687	398.4918	663.9929	1003.4648	1399.5444	1830.2660	2207.2173	2594.5272	3058.4598	3573.6170	3994.5893	4322.9803	4573.7781	4771.6728	5236.7134
1999	4.7430	47.1744	184.9742	401.1514	673.3328	1007.6688	1409.7643	1848.8349	2231.2052	2607.6429	3070.1277	3582.3923	4014.6348	4339.3261	4605.3326	4803.3500	5247.8906
2000	4.7559	47.2485	184.9089	403.3775	682.4410	1011.6446	1421.6523	1866.9259	2250.7168	2621.7032	3082.1795	3590.8212	4030.5961	4363.0049	4631.4791	4834.2186	5251.3259
2001	4.7856	47.4475	184.9076	406.4851	692.1984	1019.0491	1436.3614	1882.8757	2269.1768	2642.5358	3104.3115	3611.2259	4058.2836	4406.3652	4669.7846	4876.1403	5262.3787
2002	4.8384	47.8225	185.4618	410.5142	703.4116	1032.5915	1453.0406	1895.5656	2302.5928	2673.7093	3139.1218	3651.9147	4108.0979	4477.2716	4731.5336	4939.2612	5294.2401
2003	4.8995	48.2913	186.2955	414.4588	713.5610	1051.2067	1468.6685	1912.0054	2333.4081	2715.6658	3182.7441	3704.4559	4172.6608	4560.1203	4811.6143	5012.0825	5341.1196
2004	4.9549	48.7757	187.1840	416.9727	721.7396	1071.7221	1480.8120	1929.0931	2364.3840	2761.4379	3229.1647	3763.3975	4238.5163	4647.6207	4898.6678	5088.7019	5391.5966
2005	4.9902	49.1797	187.7839	417.1243	726.9467	1088.7960	1490.1696	1946.3773	2396.3579	2802.5707	3275.0988	3819.3766	4298.4886	4724.0093	4986.9289	5159.8225	5439.6574
2006	5.0030	49.5444	188.1842	415.7541	731.0812	1102.7376	1501.8074	1967.6725	2429.3494	2849.5764	3334.8697	3889.7226	4373.2834	4812.3779	5094.7623	5250.5369	5495.1625
2007	5.0050	49.9160	188.6894	414.6808	734.9678	1115.7080	1519.5704	1991.7247	2461.3261	2926.8104	3417.1356	3982.0189	4473.4461	4925.8123	5236.9779	5375.3775	5569.6066
2008	4.9978	50.2554	189.3330	414.1038	737.9802	1125.1652	1540.7159	2014.8963	2496.5963	3001.0937	3514.7850	4081.3130	4577.3171	5042.1932	5376.9409	5514.2432	5643.0809

2009	4.9838	50.5383	190.1322	414.0666	738.7212	1131.4915	1561.2951	2032.7371	2529.8006	3063.6359	3605.0005	4169.0949	4666.3930	5129.6822	5492.0159	5630.8959	5705.1044
2010	4.9658	50.7194	190.9927	414.2199	736.4985	1134.7929	1575.2989	2043.9944	2553.2664	3111.3468	3665.7045	4228.2286	4722.2964	5176.7008	5553.1869	5709.7984	5749.1669
2011	4.9528	50.8019	192.2585	414.6343	732.7111	1138.0005	1585.4469	2053.9015	2578.0523	3141.3716	3703.2582	4270.4783	4756.2304	5195.1567	5569.9188	5752.7152	5780.5423
2012	4.9466	50.8474	193.9169	415.5142	730.0279	1141.0274	1596.4937	2069.1713	2605.8325	3161.9035	3764.1809	4319.9603	4789.9209	5210.6300	5575.1587	5791.6545	5814.3726
2013	4.9438	50.8054	195.6149	416.7654	728.3858	1143.0733	1605.5914	2089.4610	2631.4734	3188.0911	3810.2015	4380.1239	4826.4542	5228.9735	5586.1120	5823.1496	5860.9824
2014	4.9412	50.6438	197.0072	418.2648	727.6004	1142.2691	1612.7089	2112.8288	2649.3969	3215.3373	3849.2860	4433.8567	4863.5387	5253.1692	5598.1560	5861.7967	5914.7359
2015	4.9353	50.3549	197.6829	419.7591	727.1886	1137.8693	1617.3256	2131.5440	2660.3856	3237.8278	3885.1551	4475.7610	4899.9297	5283.4715	5621.3191	5901.6140	5979.0582
2016	4.9201	50.0240	197.5324	421.8328	727.4646	1132.3581	1623.7640	2147.6190	2673.0076	3264.3215	3913.9377	4514.1173	4946.9046	5330.1923	5663.7514	5956.5839	6064.3612
2017	4.8962	49.6988	196.9056	424.3043	728.4977	1129.2781	1630.6615	2163.7769	2692.8324	3291.3898	3933.3543	4585.5473	5012.0531	5391.7194	5724.6088	6029.3609	6183.3135
2018	4.8680	49.3913	195.8020	426.6061	729.8536	1127.4364	1634.6398	2173.2944	2714.4138	3311.0165	3956.3568	4638.0148	5089.7450	5453.7863	5782.4215	6098.6716	6292.1822
2019	4.8400	49.1154	194.2778	428.3064	730.9471	1125.2151	1631.0808	2174.2517	2730.7018	3315.1857	3972.5263	4671.3569	5145.5417	5495.4167	5811.8272	6123.0728	6365.1943
2020	4.8171	48.8786	192.5538	429.1375	731.8618	1122.2714	1620.6008	2170.1479	2735.2222	3305.5484	3973.9254	4685.4385	5165.9003	5507.7308	5815.5648	6127.7715	6412.6422
2021	4.8023	48.6310	190.3840	426.7188	730.8622	1115.6397	1604.3228	2165.1848	2727.2828	3277.2877	3944.0037	4638.9743	5125.2436	5480.1085	5766.4135	6025.0510	6264.1066
2022	5.1208	48.6955	190.4695	421.1937	732.8121	1118.1030	1601.0431	2166.9185	2751.2835	3349.5701	4033.9207	4765.0108	5313.5811	5686.3568	5978.8350	6239.8617	6457.0430
2023	5.2129	48.5466	189.2913	419.2453	733.6058	1116.1658	1593.3198	2166.1125	2761.4669	3366.0892	4059.7908	4804.8846	5381.7976	5759.4445	6044.7835	6292.7004	6507.0739
2024	5.3051	48.3976	188.1131	417.2969	734.3996	1114.2286	1585.5965	2165.3065	2771.6504	3382.6084	4085.6608	4844.7585	5450.0142	5832.5322	6110.7320	6345.5392	6557.1048
2025	5.3787	48.8250	187.2863	414.6005	730.8370	1114.3490	1581.9268	2154.2450	2768.0522	3392.5450	4100.6671	4865.5407	5482.6248	5894.2554	6173.4973	6398.3800	6598.9488
2026	5.4523	49.2524	186.4595	411.9041	727.2743	1114.4694	1578.2571	2143.1835	2764.4541	3402.4815	4115.6735	4886.3228	5515.2355	5955.9786	6236.2627	6451.2208	6640.7927
2027	5.5259	49.6798	185.6327	409.2077	723.7117	1114.5897	1574.5874	2132.1220	2760.8559	3412.4181	4130.6798	4907.1049	5547.8462	6017.7018	6299.0280	6504.0617	6682.6366
2028	5.5995	50.1071	184.8059	406.5113	720.1490	1114.7101	1570.9177	2121.0605	2757.2578	3422.3547	4145.6862	4927.8870	5580.4569	6079.4250	6361.7933	6556.9025	6724.4805
2029	5.6731	50.5345	183.9791	403.8149	716.5863	1114.8305	1567.2480	2109.9990	2753.6596	3432.2912	4160.6925	4948.6692	5613.0676	6141.1483	6424.5586	6609.7434	6766.3244
2030	5.7244	50.8277	184.8013	401.5398	711.2480	1108.1655	1565.1502	2102.5496	2736.7747	3423.8189	4167.4336	4960.4162	5630.0754	6170.1317	6483.9540	6669.1316	6814.4287
2031	5.7757	51.1209	185.6235	399.2647	705.9097	1101.5006	1563.0524	2095.1002	2719.8897	3415.3465	4174.1747	4972.1632	5647.0833	6199.1150	6543.3495	6728.5199	6862.5330
2032	5.8270	51.4142	186.4458	396.9895	700.5714	1094.8356	1560.9546	2087.6508	2703.0048	3406.8741	4180.9157	4983.9102	5664.0912	6228.0984	6602.7449	6787.9081	6910.6372
2033	5.8783	51.7074	187.2680	394.7144	695.2331	1088.1706	1558.8568	2080.2013	2686.1198	3398.4017	4187.6568	4995.6572	5681.0991	6257.0818	6662.1404	6847.2964	6958.7415

2034	5.9296	52.0006	188.0902	392.4392	689.8947	1081.5057	1556.7590	2072.7519	2669.2349	3389.9294	4194.3979	5007.4042	5698.1069	6286.0652	6721.5358	6906.6847	7006.8458
2035	5.9559	52.1497	188.5068	393.1891	685.2533	1072.4473	1545.8539	2067.3217	2656.8340	3365.8962	4179.5374	5009.4708	5704.8592	6297.7465	6745.3183	6961.9430	7061.3164
2036	5.9822	52.2988	188.9234	393.9389	680.6118	1063.3889	1534.9488	2061.8914	2644.4332	3341.8631	4164.6770	5011.5373	5711.6115	6309.4278	6769.1008	7017.2012	7115.7870
2037	6.0086	52.4480	189.3399	394.6887	675.9703	1054.3305	1524.0436	2056.4611	2632.0323	3317.8300	4149.8165	5013.6039	5718.3638	6321.1091	6792.8833	7072.4595	7170.2576
2038	6.0349	52.5971	189.7565	395.4386	671.3289	1045.2721	1513.1385	2051.0309	2619.6314	3293.7969	4134.9561	5015.6705	5725.1160	6332.7904	6816.6657	7127.7178	7224.7282
2039	6.0612	52.7462	190.1731	396.1884	666.6874	1036.2138	1502.2334	2045.6006	2607.2305	3269.7637	4120.0956	5017.7370	5731.8683	6344.4716	6840.4482	7182.9761	7279.1988
2040	6.0880	52.8971	190.5934	396.9440	667.8327	1029.7802	1490.4386	2032.0225	2600.7048	3255.1716	4091.9881	5000.5812	5734.1708	6351.8297	6852.9458	7208.0537	7336.8193
2041	6.1148	53.0479	191.0136	397.6995	668.9780	1023.3466	1478.6439	2018.4444	2594.1791	3240.5795	4063.8805	4983.4254	5736.4732	6359.1877	6865.4435	7233.1313	7394.4398
2042	6.1416	53.1988	191.4339	398.4551	670.1233	1016.9131	1466.8491	2004.8664	2587.6534	3225.9874	4035.7730	4966.2696	5738.7756	6366.5457	6877.9411	7258.2088	7452.0603
2043	6.1684	53.3496	191.8542	399.2106	671.2686	1010.4795	1455.0544	1991.2883	2581.1276	3211.3953	4007.6655	4949.1137	5741.0781	6373.9037	6890.4388	7283.2864	7509.6808
2044	6.1952	53.5004	192.2744	399.9662	672.4139	1004.0460	1443.2597	1977.7102	2574.6019	3196.8032	3979.5580	4931.9579	5743.3805	6381.2617	6902.9364	7308.3640	7567.3013
2045	6.2220	53.6513	192.6947	400.7217	673.5592	997.6124	1431.4649	1964.1321	2568.0762	3182.2111	3951.4504	4914.8021	5745.6830	6388.6197	6915.4340	7333.4415	7624.9218

Table S16 Projected Analysis of Age-Specific ASIR for Male Gout Through 2045

year	male-ASIR																
	15 to 19	20 to 24	25 to 29	30 to 34	35 to 39	40 to 44	45 to 49	50 to 54	55 to 59	60 to 64	65 to 69	70 to 74	75 to 79	80 to 84	85 to 89	90 to 94	95 plus
1992	3.2996	23.7126	58.7363	95.9481	141.8425	192.1961	249.4118	303.0363	357.9694	415.1386	476.8147	526.4008	563.8905	591.1385	616.0690	680.7258	774.2566
1993	3.2957	23.6671	58.9213	96.5236	141.2006	192.1531	249.4408	303.0673	356.7461	413.6562	474.8476	522.9942	562.6699	589.7284	614.5726	679.1703	774.1996
1994	3.2931	23.5924	58.9959	97.3668	140.4447	192.0112	250.1733	303.4294	355.7777	412.7610	473.4888	521.0385	561.5468	589.3109	613.9554	677.8530	774.1760
1995	3.2896	23.4711	58.9933	98.1794	139.8570	192.4535	250.6798	303.8952	355.3950	412.4190	472.9466	520.4516	561.0431	590.3840	614.7264	677.0537	775.1465
1996	3.2880	23.3510	59.0686	98.9541	139.7251	193.0644	251.3633	304.6481	355.3866	412.6851	472.6779	520.6482	560.9372	592.8524	616.7042	677.3740	776.6479
1997	3.2909	23.2910	59.2416	99.7534	140.2853	193.7028	252.0586	306.2968	355.7718	412.7378	472.4550	521.0890	560.8827	595.9557	619.2124	679.3596	777.1521
1998	3.2969	23.2779	59.4481	100.4019	141.5896	194.1264	253.4258	307.6607	356.6200	412.7675	472.3226	521.7502	561.0219	599.4446	621.9241	681.6716	777.5706

1999	3.3052	23.3035	59.5658	100.9501	143.3290	194.2867	254.6606	309.7905	357.6656	412.8895	472.4366	522.5552	561.8313	602.3109	624.8705	683.6849	777.0604
2000	3.3140	23.3483	59.5248	101.4079	145.0993	194.4128	256.2567	311.3931	358.7518	413.3889	472.8849	523.4719	563.0723	603.9312	628.0325	685.4123	775.7047
2001	3.3342	23.4467	59.4801	102.0924	147.0914	195.2488	257.9435	313.3826	360.9505	415.1617	475.4857	525.9524	566.3219	606.0418	632.5210	687.2736	774.2789
2002	3.3701	23.6186	59.5841	103.0116	149.4753	197.3362	259.8580	315.8219	364.9804	418.9998	480.2667	531.0305	572.2906	610.1678	639.0259	690.2498	774.5394
2003	3.4116	23.8342	59.7640	103.9343	151.6784	200.4841	261.4848	319.0308	369.5234	424.2172	486.1670	537.5510	579.5380	615.3330	646.5353	693.2619	776.1041
2004	3.4495	24.0667	59.9652	104.5421	153.4488	203.9969	262.5957	321.9047	375.1113	429.9157	492.2176	544.3907	586.5815	620.9293	652.5119	695.9967	777.7006
2005	3.4740	24.2785	60.0939	104.6024	154.5082	207.0143	263.2734	324.9389	379.7607	435.1869	497.6051	550.2828	592.2100	625.4782	655.8208	699.0369	778.9561
2006	3.4837	24.4960	60.1943	104.3202	155.3212	209.4817	264.2319	327.9483	385.0817	441.6605	503.4134	556.5565	596.9870	629.8684	657.4187	703.3539	779.2471
2007	3.4863	24.7320	60.3613	104.1009	156.1818	211.7247	266.1649	331.1443	391.3762	450.7716	511.3542	563.7790	602.6020	634.8678	659.4630	709.2178	779.1975
2008	3.4826	24.9528	60.5923	103.9767	156.9167	213.3321	268.9106	333.9248	398.4554	459.9192	520.3861	571.0678	608.5154	640.0489	661.7753	715.9471	778.5278
2009	3.4741	25.1346	60.8800	103.9527	157.1907	214.3950	271.8547	335.8895	404.5743	469.1100	528.7049	577.7475	614.3023	644.8822	664.8015	721.3325	778.5504
2010	3.4622	25.2442	61.1782	103.9267	156.7825	214.9367	274.2977	336.9809	409.5148	475.1876	535.0188	583.1339	619.5826	649.5677	668.2083	724.9871	780.6705
2011	3.4533	25.2835	61.6009	103.9397	156.0120	215.5886	276.7433	338.3677	413.5117	479.8252	540.5074	587.8060	625.4437	654.4431	673.5066	728.0212	785.9657
2012	3.4492	25.2945	62.1575	104.0962	155.4257	216.4694	279.8001	341.2759	417.4464	484.2513	547.0922	593.6645	631.7772	660.6447	679.0532	732.4475	794.2486
2013	3.4472	25.2545	62.7244	104.3802	155.0100	217.3289	282.6782	345.6320	420.2891	489.6395	552.5939	600.2309	637.9981	667.2912	684.9689	737.5022	803.8113
2014	3.4454	25.1506	63.1799	104.7731	154.7501	217.7206	285.0353	350.6045	421.5835	494.0285	559.4207	606.1046	643.7403	673.3975	690.2183	742.7116	811.4137
2015	3.4413	24.9839	63.3876	105.2226	154.5534	217.3489	286.4625	354.8438	421.6819	498.3596	563.5949	611.4085	648.4277	678.2360	694.6580	747.1435	817.1143
2016	3.4304	24.8094	63.3261	105.9065	154.6423	216.7242	287.7968	358.4753	422.3971	501.7386	567.7666	616.5761	651.8403	682.1733	697.5259	750.9713	820.3917
2017	3.4131	24.6534	63.1199	106.7721	155.1117	216.4425	289.3114	361.9083	425.0602	504.6036	572.1084	621.7340	655.5745	685.2690	700.2597	753.1100	823.3063
2018	3.3926	24.5143	62.7627	107.5825	155.7120	216.2252	290.3873	364.0802	429.0428	505.6918	577.3597	625.3867	659.5265	687.6186	702.8426	754.7491	825.9641
2019	3.3724	24.3904	62.2615	108.1367	156.1721	215.7640	290.1831	364.7158	432.7234	504.6044	580.7912	631.0929	662.5440	689.6447	705.0480	756.1211	829.1836
2020	3.3558	24.2767	61.6719	108.3016	156.4179	214.7608	288.0898	362.8943	432.6854	500.0165	584.1936	634.9376	667.3471	694.5414	712.4635	765.7740	842.4345
2021	3.3467	24.1902	61.1597	108.2809	157.5581	215.1962	287.8052	363.2874	432.7302	493.9024	578.8630	630.9767	668.7582	696.9872	717.3221	768.0139	841.2666
2022	3.4721	24.2641	61.3005	106.6015	157.5436	215.1634	285.7434	364.0644	438.7614	511.1764	589.2460	648.4136	684.0259	708.3769	722.9031	772.1959	839.8950
2023	3.5041	24.2172	61.0003	106.1937	157.9856	214.9939	284.6073	364.2915	441.4874	514.3344	592.7277	654.8509	691.0723	714.1781	727.9157	776.2859	842.1888

2024	3.5362	24.1703	60.7002	105.7858	158.4277	214.8244	283.4712	364.5186	444.2133	517.4925	596.2095	661.2883	698.1188	719.9792	732.9283	780.3759	844.4825
2025	3.5610	24.2854	60.5157	105.2208	157.8191	215.2909	283.1816	363.1147	444.3990	520.2449	598.7657	663.9745	703.7236	726.2856	737.9438	784.9212	848.5368
2026	3.5859	24.4005	60.3312	104.6559	157.2105	215.7573	282.8919	361.7107	444.5846	522.9974	601.3219	666.6607	709.3284	732.5920	742.9593	789.4665	852.5910
2027	3.6107	24.5156	60.1467	104.0909	156.6019	216.2238	282.6022	360.3067	444.7703	525.7498	603.8781	669.3468	714.9332	738.8984	747.9747	794.0118	856.6452
2028	3.6356	24.6307	59.9622	103.5259	155.9933	216.6903	282.3126	358.9028	444.9559	528.5022	606.4343	672.0330	720.5380	745.2048	752.9902	798.5571	860.6995
2029	3.6604	24.7458	59.7777	102.9609	155.3847	217.1568	282.0229	357.4988	445.1416	531.2547	608.9905	674.7192	726.1428	751.5112	758.0057	803.1024	864.7537
2030	3.6774	24.8237	59.9354	102.5597	154.4509	216.1567	282.3298	356.8240	443.1094	531.0187	611.6131	676.9874	728.4510	756.8430	763.9580	807.8548	869.0081
2031	3.6943	24.9017	60.0930	102.1584	153.5172	215.1567	282.6366	356.1491	441.0773	530.7828	614.2357	679.2556	730.7591	762.1749	769.9103	812.6071	873.2624
2032	3.7112	24.9796	60.2507	101.7572	152.5834	214.1566	282.9434	355.4743	439.0451	530.5468	616.8583	681.5238	733.0673	767.5067	775.8627	817.3595	877.5168
2033	3.7282	25.0575	60.4084	101.3559	151.6497	213.1565	283.2503	354.7995	437.0129	530.3108	619.4809	683.7920	735.3754	772.8386	781.8150	822.1119	881.7711
2034	3.7451	25.1355	60.5660	100.9546	150.7160	212.1565	283.5571	354.1246	434.9807	530.0749	622.1035	686.0602	737.6836	778.1704	787.7673	826.8642	886.0255
2035	3.7537	25.1748	60.6455	101.0742	150.0098	210.7470	282.0513	354.1665	433.8109	527.2966	621.3267	688.3838	739.5271	779.9921	792.6699	832.5835	890.4728
2036	3.7623	25.2141	60.7249	101.1937	149.3036	209.3375	280.5456	354.2084	432.6411	524.5183	620.5498	690.7073	741.3706	781.8137	797.5725	838.3028	894.9202
2037	3.7709	25.2535	60.8044	101.3133	148.5974	207.9280	279.0398	354.2503	431.4712	521.7399	619.7730	693.0309	743.2141	783.6353	802.4752	844.0221	899.3676
2038	3.7795	25.2928	60.8838	101.4328	147.8912	206.5185	277.5341	354.2921	430.3014	518.9616	618.9961	695.3544	745.0576	785.4570	807.3778	849.7414	903.8149
2039	3.7881	25.3321	60.9633	101.5523	147.1850	205.1090	276.0283	354.3340	429.1316	516.1833	618.2193	697.6780	746.9011	787.2786	812.2804	855.4607	908.2623
2040	3.7967	25.3717	61.0431	101.6724	147.3466	204.2049	274.2873	352.5336	429.1804	514.8416	615.0761	696.8264	749.3878	789.2205	814.1655	860.6965	914.4262
2041	3.8054	25.4113	61.1230	101.7925	147.5082	203.3008	272.5463	350.7331	429.2292	513.4998	611.9330	695.9749	751.8745	791.1624	816.0507	865.9323	920.5901
2042	3.8141	25.4509	61.2029	101.9127	147.6698	202.3967	270.8052	348.9327	429.2781	512.1581	608.7898	695.1234	754.3612	793.1043	817.9359	871.1681	926.7540
2043	3.8227	25.4905	61.2828	102.0328	147.8314	201.4925	269.0642	347.1323	429.3269	510.8163	605.6467	694.2719	756.8479	795.0461	819.8210	876.4039	932.9178
2044	3.8314	25.5300	61.3626	102.1529	147.9930	200.5884	267.3232	345.3318	429.3757	509.4746	602.5035	693.4204	759.3346	796.9880	821.7062	881.6397	939.0817
2045	3.8401	25.5696	61.4425	102.2730	148.1546	199.6843	265.5822	343.5314	429.4246	508.1328	599.3604	692.5689	761.8213	798.9299	823.5913	886.8755	945.2456

Table S17 Projected Analysis of Age-Specific ASDR for Male Gout Through 2045

year	male-ASDR																
	15 to 19	20 to 24	25 to 29	30 to 34	35 to 39	40 to 44	45 to 49	50 to 54	55 to 59	60 to 64	65 to 69	70 to 74	75 to 79	80 to 84	85 to 89	90 to 94	95 plus
1992	0.1662	1.6794	6.2025	12.7356	21.7757	32.2276	44.2034	56.2655	68.5140	80.4231	94.3868	108.2808	118.1532	124.6938	127.6985	132.8661	142.6950
1993	0.1660	1.6772	6.2204	12.8314	21.8198	32.2647	44.2816	56.5404	68.3944	79.9314	93.4831	107.0468	116.5902	123.4107	126.0185	130.7582	140.1445
1994	0.1659	1.6720	6.2269	12.9528	21.7403	32.3701	44.5613	56.8722	68.2759	79.7773	92.9125	106.3742	115.4149	122.6843	125.0661	129.1453	138.4657
1995	0.1657	1.6640	6.2424	13.0785	21.7010	32.5528	44.7486	57.2248	68.3434	79.7974	92.7858	106.0652	115.1848	122.3912	125.0196	128.4213	138.0170
1996	0.1656	1.6552	6.2393	13.1694	21.7142	32.7200	45.1166	57.5089	68.6469	80.0596	92.7934	106.2050	115.4247	122.5571	125.6812	128.4988	138.3048
1997	0.1658	1.6518	6.2684	13.3149	21.8487	32.8873	45.2153	58.2811	69.1576	80.3878	92.9389	106.3588	115.9710	122.8564	126.4614	128.9557	138.5277
1998	0.1661	1.6508	6.2873	13.3880	22.0960	33.0820	45.5013	58.7970	69.8509	80.7640	93.3067	106.4774	116.4786	123.1954	127.2470	129.7816	138.8998
1999	0.1665	1.6529	6.2903	13.4770	22.4102	33.2152	45.8664	59.3625	70.6515	81.1514	93.5969	106.7156	117.0796	123.6192	128.0824	130.5839	139.2041
2000	0.1670	1.6540	6.2775	13.5631	22.7232	33.3272	46.2508	59.9828	71.2733	81.5495	93.9127	107.0143	117.5319	124.2658	128.7054	131.3444	139.2077
2001	0.1680	1.6591	6.2693	13.6832	23.0488	33.6184	46.6728	60.5450	71.8789	82.1192	94.5892	107.6643	118.2809	125.6156	129.8794	132.3941	139.3497
2002	0.1699	1.6707	6.3213	13.8198	23.4207	34.0494	47.2265	60.9576	72.9153	83.0640	95.7171	108.8595	119.7846	127.4950	131.4638	134.1096	140.2729
2003	0.1720	1.6876	6.3482	13.9430	23.7841	34.7205	47.7964	61.4561	73.8608	84.5114	97.0253	110.3982	121.6609	129.8434	133.6775	135.9412	141.4548
2004	0.1740	1.7051	6.3652	14.0309	24.0404	35.3415	48.2264	61.9725	74.8628	85.8750	98.4085	112.2332	123.4752	132.3238	135.9311	138.0632	142.7129
2005	0.1752	1.7180	6.3977	14.0493	24.2131	35.8833	48.4605	62.5931	75.8853	87.1665	99.8567	113.9024	125.2290	134.5516	138.2789	139.9369	143.8046
2006	0.1756	1.7311	6.4144	14.0019	24.3497	36.3852	48.8416	63.2702	76.9710	88.7088	101.6874	115.9948	127.4409	136.9817	141.3147	142.3109	145.4737
2007	0.1757	1.7444	6.4310	13.9833	24.4551	36.7919	49.4165	64.0763	77.9700	91.0955	104.1620	118.7877	130.3381	140.1783	145.2591	145.7376	147.3498
2008	0.1755	1.7567	6.4549	13.9480	24.5878	37.1715	50.1527	64.8689	79.1831	93.3879	107.2022	121.7305	133.2375	143.5102	149.2349	149.4988	149.3963
2009	0.1750	1.7652	6.4695	13.9257	24.5961	37.3642	50.8417	65.4369	80.2527	95.4255	109.8711	124.4004	135.8657	145.8424	152.3376	152.6316	151.0295
2010	0.1743	1.7706	6.4865	13.9257	24.5390	37.4725	51.2989	65.6856	80.9317	96.7544	111.6630	126.0619	137.6241	147.2183	154.0470	154.7963	152.2845
2011	0.1739	1.7726	6.5407	13.9509	24.4074	37.6122	51.6369	65.9993	81.7365	97.8247	112.8655	127.2855	138.5755	147.7668	154.5048	156.0058	153.0908
2012	0.1737	1.7755	6.5942	13.9947	24.3323	37.6897	51.9765	66.6184	82.5917	98.3568	114.7038	128.7289	139.5694	148.2152	154.7701	156.9975	154.0345

2013	0.1736	1.7745	6.6661	14.0265	24.2936	37.7215	52.3580	67.2593	83.4554	99.2712	116.1057	130.6316	140.5916	148.8862	155.1163	157.9355	155.3380
2014	0.1735	1.7665	6.7175	14.0562	24.2472	37.7077	52.5699	68.0866	83.9284	100.0353	117.3835	132.2733	141.7289	149.5551	155.4405	158.9329	156.8377
2015	0.1733	1.7571	6.7344	14.1321	24.2352	37.5525	52.6709	68.6101	84.3210	100.7335	118.4605	133.5215	142.8723	150.4274	156.1010	160.0789	158.5810
2016	0.1727	1.7449	6.7343	14.1916	24.2255	37.3656	52.8571	69.1175	84.6997	101.5691	119.3016	134.5456	144.1221	151.7170	157.2837	161.7143	160.8071
2017	0.1719	1.7343	6.7050	14.2847	24.2363	37.2298	53.0760	69.6306	85.3164	102.4288	119.8952	136.6184	146.0616	153.4512	158.9248	163.5753	163.9837
2018	0.1709	1.7229	6.6581	14.3764	24.2718	37.2236	53.1961	69.9787	86.0021	103.0363	120.4810	138.1241	148.2248	155.1151	160.4500	165.4712	166.8684
2019	0.1699	1.7132	6.6096	14.4178	24.3170	37.1349	53.1318	69.9655	86.5752	103.0271	120.9549	139.0348	149.7417	156.2527	161.2814	166.0832	168.6917
2020	0.1691	1.7056	6.5416	14.4511	24.3093	36.9953	52.6966	69.8269	86.5528	102.6196	120.9704	139.4111	150.3250	156.4749	161.3652	166.1261	169.8189
2021	0.1686	1.6973	6.4747	14.3569	24.2686	36.7065	52.1042	69.5646	86.1923	101.6408	119.9258	138.0395	148.9864	155.7492	159.8691	163.2342	166.1186
2022	0.1790	1.6986	6.4742	14.1603	24.3648	36.8289	52.0563	69.6616	87.1185	104.0841	122.7668	141.8416	154.6098	161.6195	165.8116	169.0980	171.0283
2023	0.1820	1.6933	6.4328	14.0876	24.3928	36.7533	51.7953	69.6179	87.4432	104.5968	123.5391	143.0267	156.5796	163.6781	167.6160	170.4946	172.3094
2024	0.1850	1.6880	6.3914	14.0150	24.4207	36.6776	51.5344	69.5742	87.7679	105.1095	124.3115	144.2118	158.5494	165.7368	169.4205	171.8913	173.5906
2025	0.1874	1.7018	6.3638	13.9240	24.2959	36.6857	51.4027	69.2111	87.6382	105.4225	124.7705	144.8169	159.4931	167.4872	171.1481	173.3178	174.6822
2026	0.1897	1.7155	6.3363	13.8329	24.1712	36.6937	51.2711	68.8481	87.5084	105.7356	125.2294	145.4220	160.4369	169.2375	172.8758	174.7443	175.7738
2027	0.1921	1.7293	6.3088	13.7419	24.0464	36.7018	51.1395	68.4850	87.3787	106.0486	125.6884	146.0271	161.3806	170.9878	174.6035	176.1709	176.8654
2028	0.1945	1.7431	6.2813	13.6509	23.9216	36.7098	51.0079	68.1220	87.2489	106.3617	126.1474	146.6322	162.3244	172.7382	176.3312	177.5974	177.9570
2029	0.1969	1.7569	6.2538	13.5598	23.7968	36.7179	50.8762	67.7589	87.1192	106.6748	126.6064	147.2374	163.2681	174.4885	178.0588	179.0239	179.0486
2030	0.1985	1.7663	6.2798	13.4860	23.6210	36.4928	50.8179	67.5094	86.5807	106.3994	126.8253	147.5995	163.7585	175.3185	179.7168	180.6333	180.3278
2031	0.2002	1.7757	6.3058	13.4123	23.4452	36.2678	50.7595	67.2599	86.0423	106.1241	127.0443	147.9616	164.2490	176.1484	181.3747	182.2427	181.6071
2032	0.2018	1.7852	6.3318	13.3385	23.2695	36.0427	50.7012	67.0103	85.5038	105.8487	127.2632	148.3238	164.7394	176.9784	183.0327	183.8521	182.8863
2033	0.2035	1.7946	6.3578	13.2647	23.0937	35.8177	50.6428	66.7608	84.9653	105.5733	127.4822	148.6859	165.2298	177.8084	184.6906	185.4615	184.1655
2034	0.2051	1.8041	6.3838	13.1909	22.9179	35.5927	50.5844	66.5112	84.4269	105.2980	127.7011	149.0480	165.7202	178.6383	186.3486	187.0709	185.4447
2035	0.2059	1.8088	6.3969	13.2144	22.7699	35.2996	50.2266	66.3540	84.0275	104.5509	127.2408	149.1338	165.9396	178.9752	187.0252	188.5931	186.8994
2036	0.2068	1.8136	6.4101	13.2379	22.6219	35.0065	49.8687	66.1969	83.6282	103.8039	126.7806	149.2196	166.1590	179.3120	187.7019	190.1154	188.3541
2037	0.2076	1.8184	6.4232	13.2614	22.4739	34.7134	49.5108	66.0397	83.2289	103.0568	126.3203	149.3054	166.3783	179.6488	188.3785	191.6377	189.8087

2038	0.2085	1.8232	6.4364	13.2849	22.3259	34.4204	49.1529	65.8825	82.8296	102.3098	125.8601	149.3912	166.5977	179.9857	189.0552	193.1600	191.2634
2039	0.2093	1.8280	6.4495	13.3084	22.1780	34.1273	48.7951	65.7254	82.4302	101.5627	125.3998	149.4770	166.8170	180.3225	189.7319	194.6823	192.7181
2040	0.2102	1.8329	6.4628	13.3321	22.2135	33.9236	48.4175	65.2838	82.2436	101.0996	124.5403	148.9548	166.9109	180.5572	190.0853	195.3830	194.2769
2041	0.2110	1.8377	6.4761	13.3558	22.2491	33.7198	48.0399	64.8422	82.0570	100.6365	123.6808	148.4327	167.0048	180.7919	190.4388	196.0837	195.8357
2042	0.2119	1.8426	6.4893	13.3794	22.2847	33.5161	47.6622	64.4006	81.8703	100.1734	122.8212	147.9105	167.0987	181.0265	190.7922	196.7844	197.3945
2043	0.2128	1.8474	6.5026	13.4031	22.3203	33.3124	47.2846	63.9590	81.6837	99.7103	121.9617	147.3884	167.1925	181.2612	191.1457	197.4851	198.9532
2044	0.2136	1.8522	6.5159	13.4268	22.3559	33.1087	46.9070	63.5174	81.4970	99.2472	121.1021	146.8662	167.2864	181.4959	191.4991	198.1859	200.5120
2045	0.2145	1.8571	6.5291	13.4504	22.3915	32.9050	46.5294	63.0758	81.3104	98.7841	120.2426	146.3441	167.3803	181.7306	191.8525	198.8866	202.0708

Table S18 Cross-national inequalities in the burden of gout in 2021

	Health inequality metrics	Year	Value	95% CI
**Prevalence**	Slope index of inequality	1990	378	304 to 452
		2021	799	698 to 908
	Health concentration index	1990	0.25	0.22 to 0.29
		2021	0.36	0.32 to 0.40
**Incidence**	Slope index of inequality	1990	57	47 to 67
		2021	102	88 to 116
	Health concentration index	1990	0.18	0.15 to 0.21
		2021	0.28	0.25 to 0.31
**DALY**	Slope index of inequality	1990	12	10 to 14
		2021	24	21 to 28
	Health concentration index	1990	0.25	0.21 to 0.28
		2021	0.35	0.32 to 0.39

Table S19 GBD database risk factors at all levels in 2021

Level 1 risks	Level 2 risks	Level 3 risks	Level 4 risks
Environmental/occupational risks	Unsafe water, sanitation, and handwashing	Unsafe water source	
Environmental/occupational risks	Unsafe water, sanitation, and handwashing	Unsafe sanitation	
Environmental/occupational risks	Unsafe water, sanitation, and handwashing	No access to handwashing facility	
Environmental/occupational risks	Air pollution	Particulate matter pollution	Ambient particulate matter pollution
Environmental/occupational risks	Air pollution	Particulate matter pollution	Household air pollution from solid fuels
Environmental/occupational risks	Air pollution	Ambient ozone pollution	
Environmental/occupational risks	Air pollution	Nitrogen dioxide pollution	
Environmental/occupational risks	Non-optimal temperature	High temperature	
Environmental/occupational risks	Non-optimal temperature	Low temperature	
Environmental/occupational risks	Other environmental risks	Residential radon	
Environmental/occupational risks	Other environmental risks	Lead exposure	
Environmental/occupational risks	Occupational risks	Occupational carcinogens	Occupational exposure to asbestos
Environmental/occupational risks	Occupational risks	Occupational carcinogens	Occupational exposure to arsenic

Environmental/occupational risks	Occupational risks	Occupational carcinogens	Occupational exposure to benzene
Environmental/occupational risks	Occupational risks	Occupational carcinogens	Occupational exposure to beryllium
Environmental/occupational risks	Occupational risks	Occupational carcinogens	Occupational exposure to cadmium
Environmental/occupational risks	Occupational risks	Occupational carcinogens	Occupational exposure to chromium
Environmental/occupational risks	Occupational risks	Occupational carcinogens	Occupational exposure to diesel engine exhaust
Environmental/occupational risks	Occupational risks	Occupational carcinogens	Occupational exposure to formaldehyde
Environmental/occupational risks	Occupational risks	Occupational carcinogens	Occupational exposure to nickel
Environmental/occupational risks	Occupational risks	Occupational carcinogens	Occupational exposure to polycyclic aromatic hydrocarbons
Environmental/occupational risks	Occupational risks	Occupational carcinogens	Occupational exposure to silica
Environmental/occupational risks	Occupational risks	Occupational carcinogens	Occupational exposure to sulfuric acid
Environmental/occupational risks	Occupational risks	Occupational carcinogens	Occupational exposure to trichloroethylene
Environmental/occupational risks	Occupational risks	Occupational asthmagens	
Environmental/occupational risks	Occupational risks	Occupational particulate matter, gases, and fumes	
Environmental/occupational risks	Occupational risks	Occupational noise	
Environmental/occupational risks	Occupational risks	Occupational injuries	
Environmental/occupational risks	Occupational risks	Occupational ergonomic factors	
Behavioral risks	Child and maternal malnutrition	Suboptimal breastfeeding	Non-exclusive breastfeeding
Behavioral risks	Child and maternal malnutrition	Suboptimal breastfeeding	Discontinued breastfeeding
Behavioral risks	Child and maternal malnutrition	Child growth failure	Child underweight
Behavioral risks	Child and maternal malnutrition	Child growth failure	Child wasting
Behavioral risks	Child and maternal malnutrition	Child growth failure	Child stunting
Behavioral risks	Child and maternal malnutrition	Low birth weight and short gestation	Short gestation

Behavioral risks	Child and maternal malnutrition	Low birth weight and short gestation	Low birth weight
Behavioral risks	Child and maternal malnutrition	Iron deficiency	
Behavioral risks	Child and maternal malnutrition	Vitamin A deficiency	
Behavioral risks	Child and maternal malnutrition	Zinc deficiency	
Behavioral risks	Tobacco	Smoking	
Behavioral risks	Tobacco	Chewing tobacco	
Behavioral risks	Tobacco	Secondhand smoke	
Behavioral risks	High alcohol use		
Behavioral risks	Drug use		
Behavioral risks	Dietary risks	Diet low in fruits	
Behavioral risks	Dietary risks	Diet low in vegetables	
Behavioral risks	Dietary risks	Diet low in legumes	
Behavioral risks	Dietary risks	Diet low in whole grains	
Behavioral risks	Dietary risks	Diet low in nuts and seeds	
Behavioral risks	Dietary risks	Diet low in milk	
Behavioral risks	Dietary risks	Diet high in red meat	
Behavioral risks	Dietary risks	Diet high in processed meat	
Behavioral risks	Dietary risks	Diet high in sugar-sweetened beverages	
Behavioral risks	Dietary risks	Diet low in fiber	
Behavioral risks	Dietary risks	Diet low in calcium	
Behavioral risks	Dietary risks	Diet low in seafood omega-3 fatty acids	
Behavioral risks	Dietary risks	Diet low in polyunsaturated fatty acids	
Behavioral risks	Dietary risks	Diet high in trans fatty acids	
Behavioral risks	Dietary risks	Diet high in sodium	
Behavioral risks	Intimate partner violence		
Behavioral risks	Childhood sexual abuse and bullying	Childhood sexual abuse	
Behavioral risks	Childhood sexual abuse and bullying	Bullying victimization	
Behavioral risks	Unsafe sex		
Behavioral risks	Low physical activity		
Metabolic risks	High fasting plasma glucose		
Metabolic risks	High LDL cholesterol		
Metabolic risks	High systolic blood pressure		
Metabolic risks	High body-mass index		
Metabolic risks	Low bone mineral density		

Metabolic risks	Kidney dysfunction		
